# Transamidation-Driven Molecular Pumps

**DOI:** 10.1021/jacs.2c06807

**Published:** 2022-08-18

**Authors:** Lorna Binks, Chong Tian, Stephen D. P. Fielden, Iñigo J. Vitorica-Yrezabal, David A. Leigh

**Affiliations:** Department of Chemistry, University of Manchester, Oxford Road, Manchester M13 9PL, United Kingdom

## Abstract

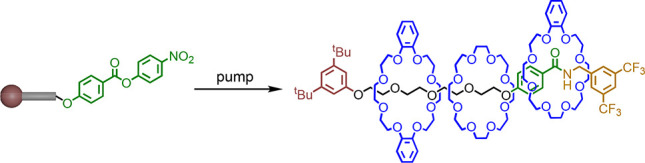

We report a new class of synthetic molecular pumps that use a stepwise information ratchet mechanism to achieve the kinetic gating required to sequester their macrocyclic substrates from bulk solution. Threading occurs as a result of active template reactions between the pump terminus amine and an acyl electrophile, whereby the bond-forming reaction is accelerated through the cavity of a crown ether. Carboxylation of the resulting amide results in displacement of the ring to the collection region of the thread. Conversion of the carbamate to a phenolic ester provides an intermediate rotaxane suitable for further pumping cycles. In this way rings can be ratcheted onto a thread from one or both ends of appropriately designed molecular pumps. Each pumping cycle results in one additional ring being added to the thread per terminus acyl group. The absence of pseudorotaxane states ensures that no dethreading of intermediates occurs during the pump operation. This facilitates the loading of different macrocycles in any chosen sequence, illustrated by the pump-mediated synthesis of a [4]rotaxane containing three different macrocycles as a single sequence isomer. A [5]rotaxane synthesized using a dual-opening transamidation pump was structurally characterized by single-crystal X-ray diffraction, revealing a series of stabilizing CH···O interactions between the crown ethers and the polyethylene glycol catchment region of the thread.

## Introduction

Protein pumps actively transport substrates away from equilibrium.^1−4^ These biomolecular machines are generally extremely structurally complex, assembled from multiple protein subunits and having molecular masses in excess of 500 kDa. A number of much smaller artificial molecular pumps have been designed.^5−24^ These minimalist systems can provide insights into the basic mechanisms required to drive chemical systems away from equilibrium^25,26^ and also illustrate well how different structural modules can be combined to generate function that goes far beyond that of the sum of the individual parts.^7,27^

Synthetic molecular pumps based on pseudorotaxane architectures have been used to drive systems away from equilibrium by progressively sequestering macrocycles from bulk solution to thermodynamically less favorable sites on collection threads.^12−21^ Accordingly, the macrocycles are trapped in a high energy state on the axle compared to unthreaded rings in solution. This constitutes active transport of the rings from bulk solution to the collection thread.^20,21^ Accordingly, the pumping needs to be powered and to occur under kinetic control. The chemical structure of the pump is designed to promote macrocycle threading and inhibit dethreading. Each pumping cycle builds on the last by increasing the concentration of macrocycles held on the collection thread. In this way, molecular pumping also enables the synthesis of well-defined higher order oligo- and polyrotaxanes and catenanes that would be inaccessible through conventional “passive” template synthesis.^13,18,28−32^

Most of the rotaxane-based pumps reported to date employ energy ratchet^5^ mechanisms, which rely on periodic variations in the binding affinities and kinetic barriers between the macrocycle and various sites on the pump. The different conditions that occur over the operation cycle define the energy surface accessible to the macrocycle, inhibiting dethreading and driving the ring onto the collection thread. A range of stimuli have been employed to drive such systems, including transition metal coordination,^29,30^ acid/base cycling,^13,21,31^ radical pairing,^12,14−18,20^ and photoisomerizations.^22−24,33,34^ Pumping by information ratchet mechanisms^35−38^ has also been demonstrated with artificial molecular pumps.^19^ Such systems rely on kinetic asymmetry,^36−39^ arising from transition state energy differences that depend on the mechanical state of the pump. Information ratchets can operate autonomously in a chemostated environment^40^ and likely form the mechanism for most or all biomolecular pumps.^36^

Here we report a new type of synthetic information ratchet pump, **1**, which operates through iterative transamidation. Pump **1** operates in a stepwise manner with no dethreadable intermediates, enabling sequence-controlled pumping of different macrocycles onto collection threads.

## Results and Discussion

### Design and Operation of Single-Opening Transamidation Pump **1**

**Scheme 1:**
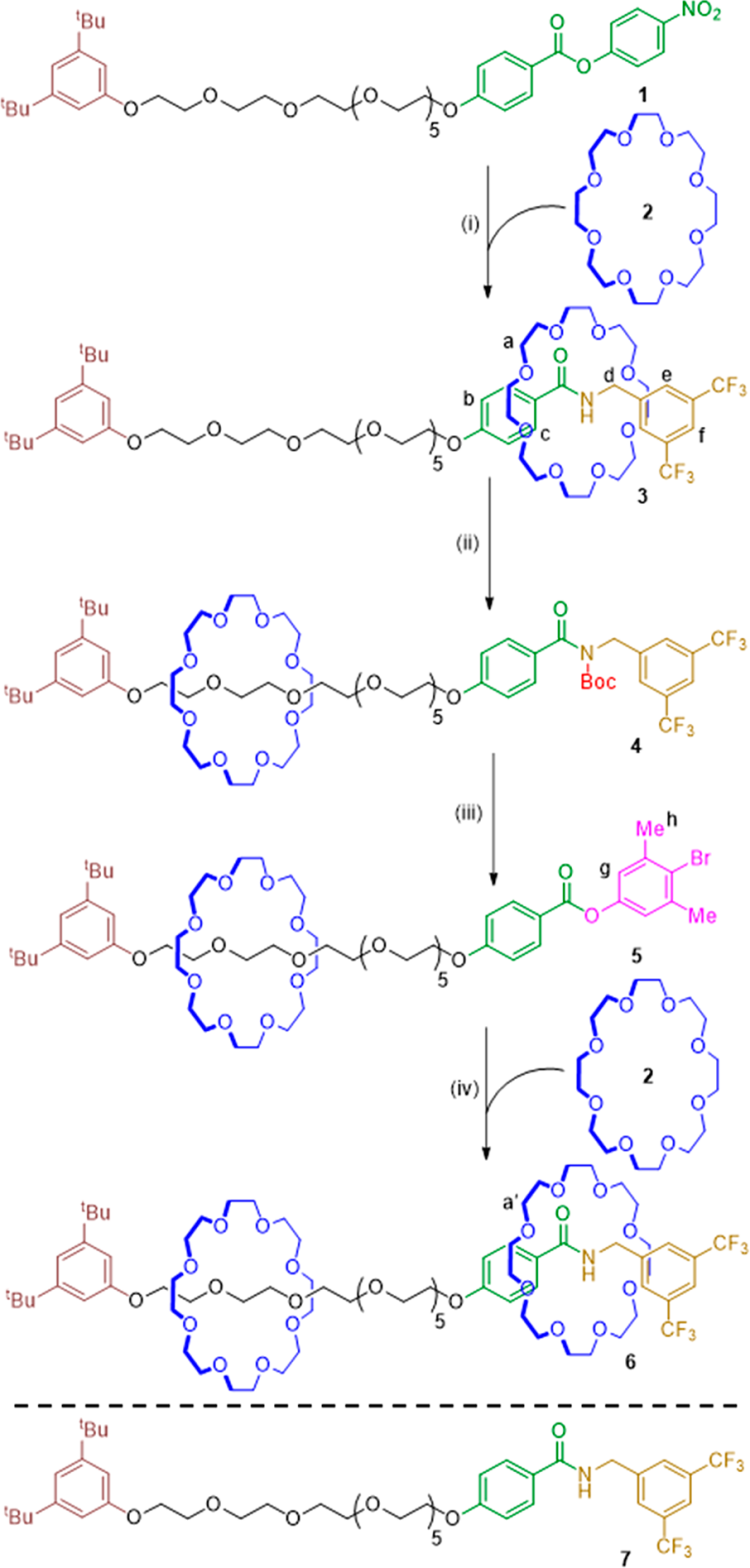
Operation of Single-Opening Transamidation Molecular Pump **1** Reagents and conditions: (i) 3,5-bis-trifluoromethylbenzylamine (1.0 equiv), **2** (1.0 equiv), toluene, rt, 16 h, 65%; (ii) Boc_2_O (6.0 equiv), DMAP (0.2 equiv), THF, 90 °C, 10 h, microwave irradiation, 77%; (iii) 4-bromo-3,5-dimethylphenol (1.0 equiv), K_3_PO_4_ (1.5 equiv), THF, 60 °C, 16 h, microwave irradiation, 68%; (iv) 3,5-bis-trifluoromethylbenzylamine (2.0 equiv), **2** (2.0 equiv), toluene, rt, 10 days, 50%.

**Figure 1:**
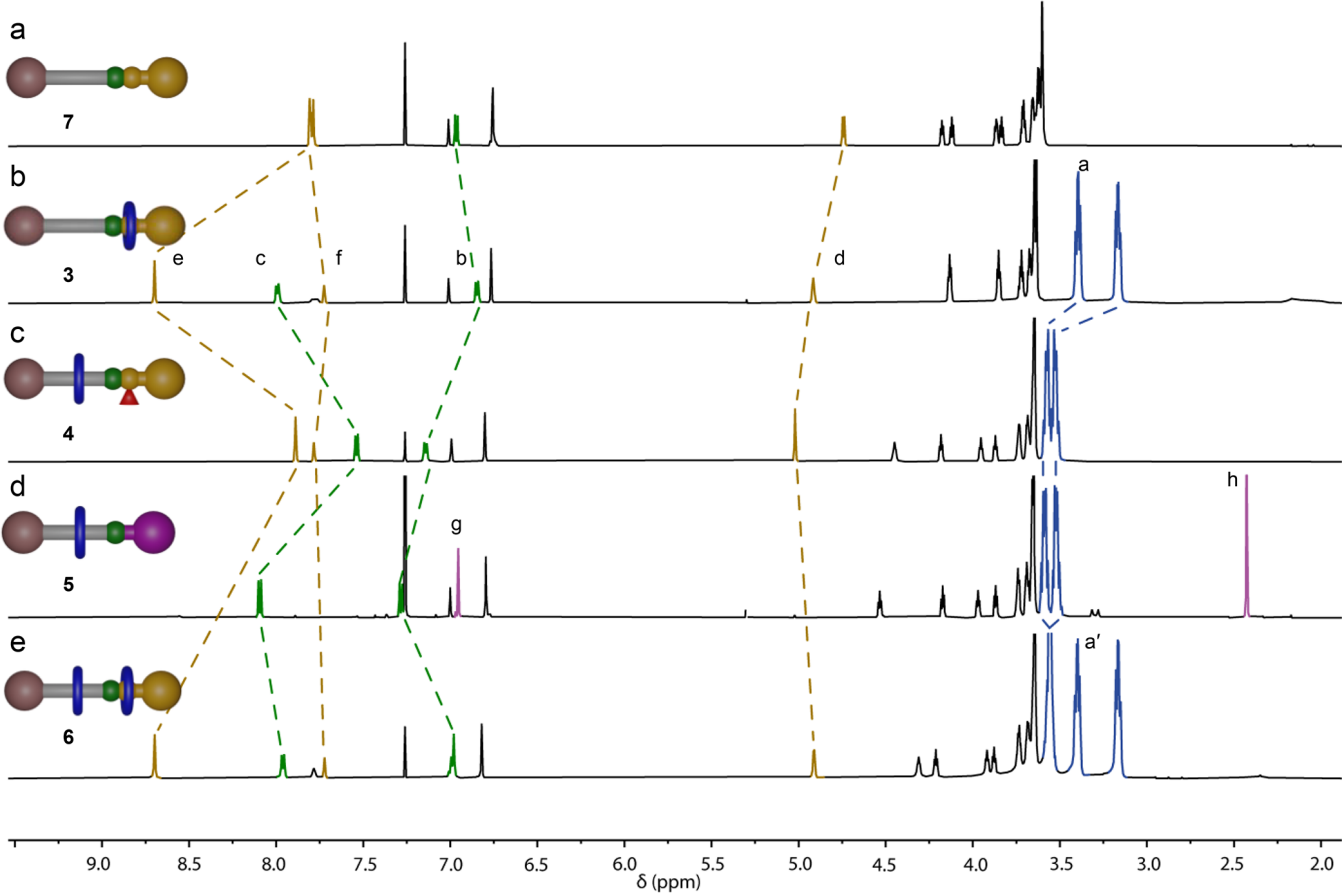
Partial ^1^H NMR spectra (600 Hz, 298 K, CDCl_3_) of the pumping cycle of **1**: (a) non-interlocked thread **7**; (b) amide [2]rotaxane **3**; (c) Boc-activated [2]rotaxane **4**; (d) ester [2]rotaxane **5**; (e) amide [3]rotaxane **6**. For proton labeling, see Scheme 1.

Pump **1**, with a single opening for ring-threading, was synthesized as outlined in the Supporting Information (Scheme S1). Its mechanism exploits metal-free active template rotaxane synthesis,^41−44^ in which the transition state of a thread-forming reaction between a primary amine and an electrophile is stabilized through the cavity of a crown ether. This results in kinetically controlled trapping of the threaded components.^19,41−45^ We chose to focus on *N*-acylation for the active template reaction, as this had previously been found^43^ to be particularly selective toward rotaxane formation over the background reaction that generates the non-interlocked thread. Treatment of **1** with 3,5-bis-trifluoromethylbenzylamine and 24-crown-8 **2** for 16 h in toluene afforded [2]rotaxane **3** in 65% yield (Scheme 1, step i). The threaded structure of **3** was confirmed by ^1^H NMR, where characteristic diastereotopic splitting of the protons on the different faces of the macrocycle (H_a_, see Scheme 1 for proton labeling) results from threading onto an unsymmetric axle (Figure 1b). Downfield shifts of the benzylic and aromatic protons (H_d_ and H_e_, from 4.74 to 4.91 ppm and 7.79 to 8.69 ppm, respectively) in **3** compared to those in the non-interlocked thread, **7**, indicate that the macrocycle is sited over the amide in the [2]rotaxane.

We envisaged that converting the amide in [2]rotaxane **3** to a reactive electrophile would allow further macrocycles to be pumped onto the thread via transamidation.^46,47^ We were inspired by recent methodology reported by Szostak and co-workers,^48,49^ in which *N*-carboxylated amides were shown to undergo transamidation reactions. We reasoned that derivatizing the amide of **3** should also remove its ability to donate hydrogen bonds and thus weaken intercomponent binding and promote shuttling of the macrocycle to the oligo(ethylene glycol) region of the collection thread. Reaction of **3** with di-*tert*-butyl decarbonate (Boc_2_O) (see Supporting Information, Table S1, for optimization studies on the amide activation step) gave [2]rotaxane **4** in 77% yield (Scheme 1, step ii).

Shuttling of the macrocycle to the collection thread upon conversion of **3** to **4** was confirmed by ^1^H NMR (Figure 1c). Signals for H_a_ shifted downfield from 3.42 to 3.60 and 3.18 to 3.55 ppm, together with more modest shifts to the other thread protons proximal to the amide (H_b_, H_c_, H_d_, H_e_, and H_f_). The chemical shifts of H_e_ and H_f_ in **4** are similar to those in non-interlocked thread **7** (Figure 1a), consistent with the displacement of the macrocycle away from the amide.

However, no reaction occurred when [2]rotaxane **4** was subsequently treated with 3,5-bis-trifluoromethylbenzylamine and crown ether **2** in toluene. The Boc-amide was not sufficiently electrophilic and/or too sterically hindered to bring about [3]rotaxane formation in the nonpolar solvents required for the active template reaction. To overcome this issue, we reasoned that a nucleophilic bulky phenol might be able to generate a more electrophilic rotaxane intermediate containing a phenolic ester.^42−45^ Active template aminolysis of this ester would then give the [3]rotaxane and regenerate the phenol.

Reaction of [2]rotaxane **4** with 4-bromo-3,5-dimethylphenol and potassium phosphate in THF (for reaction optimization see Table S2, Supporting Information) smoothly generated ester [2]rotaxane **5** in 68% yield (Scheme 1, step iii). The chemical shifts of macrocyclic protons H_a_ in **5** are almost unchanged from **4**, indicating that the macrocycle remains located on the glycol region of the collection thread.

Pleasingly, the phenolic ester [2]rotaxane **5** enabled [3]rotaxane formation as envisaged: treatment of **5** with 3,5-bis-trifluoromethylbenzylamine and 24-crown-8 **2** resulted in [3]rotaxane **6** in 50% yield (Scheme 1, step iv) to complete a second pumping cycle. The ^1^H NMR spectrum of [3]rotaxane **6** (Figure 1e) shows two sets of macrocyclic signals, one set at chemical shifts similar to those in **3** (Figure 1b) and the other similar to those in **4** (Figure 1c) and **5** (Figure 1d). This is consistent with one macrocycle in **6** residing on the collection chain, while the other binds to the newly formed amide.

### Synthesis of a Single-Sequence [4]Rotaxane (**13**) Using a Single-Opening Transamidation Molecular Pump

In principle, the pumping cycle shown in Scheme 1, steps ii–iv, can be repeated over and over again, pumping on additional rings (one per cycle) until the catchment region of the thread is full. A distinctive feature of the mechanism is that at no point in the pumping cycle are captured macrocycles able to dethread, as the intermediate pump states are all rotaxanes (dethreading is prevented by bulky stoppers on both ends of the axle), rather than pseudorotaxanes, where dethreading is only slowed by “speed bumps”. This should enable the pump to be used to synthesize oligo- or polyrotaxanes with a single sequence of structurally distinct macrocycles pumped in a specific order.^21,29,30,50^

**Scheme 2:**
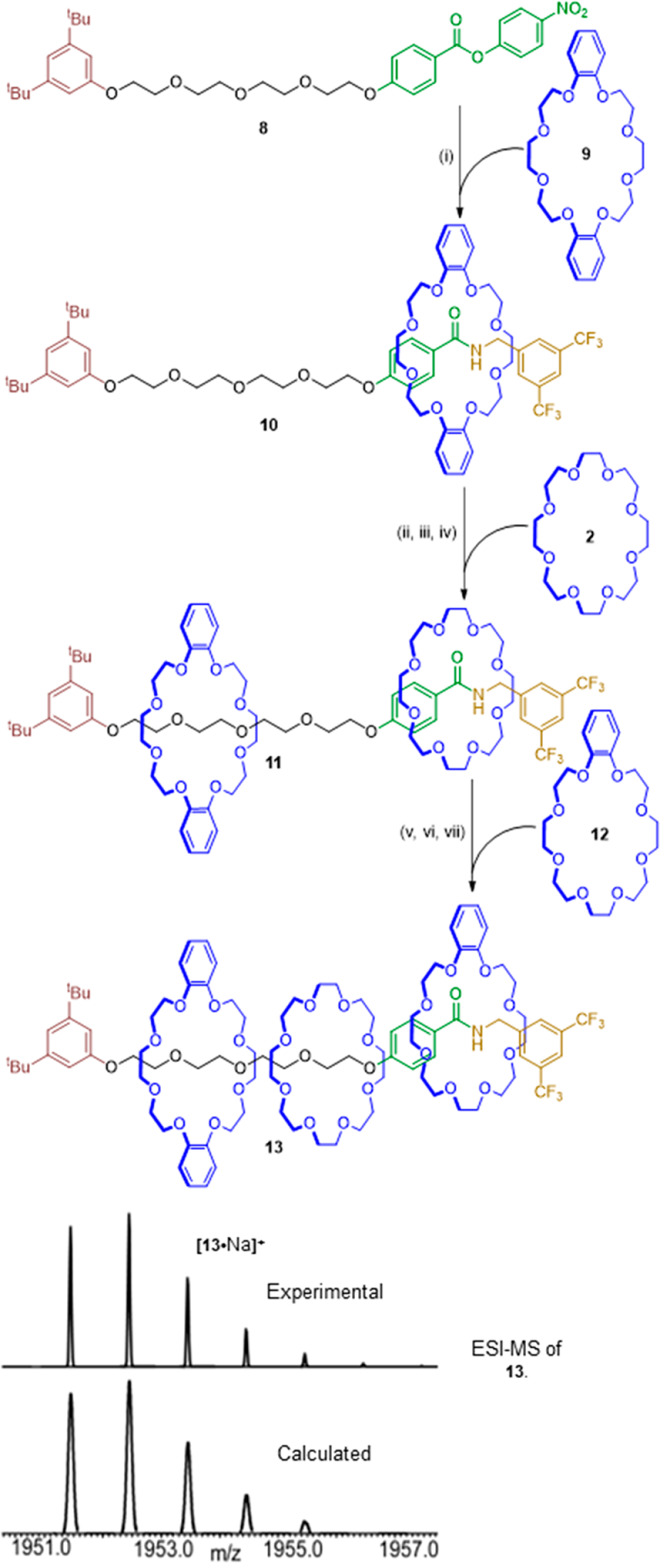
Synthesis of Single-Sequence [4]Rotaxane **13** Reagents and conditions: (i) 3,5-bis-trifluoromethylbenzylamine (1.5 equiv), **9** (1.5 equiv), toluene, rt, 16 h, 61% ([2]rotaxane:free thread ratio 5:1, determined by ^1^H NMR, in the reaction mixture prior to workup); (ii) Boc_2_O (6.0 equiv), DMAP (1.2 equiv), THF, 90 °C, 10 h, microwave irradiation, 81%; (iii) 4-bromo-3,5-dimethylphenol (3.0 equiv), K_3_PO_4_ (4.5 equiv), THF, 70 °C, 8 h, microwave irradiation, 90%; (iv) 3,5-bis-trifluoromethylbenzylamine (2.0 equiv), **2** (2.0 equiv), toluene, rt, 7 days, 54%. (v) Boc_2_O (6.0 equiv), DMAP (1.2 equiv), THF, 80 °C, 4 h, microwave irradiation, 75%; (vi) 4-bromo-3,5-dimethylphenol (3.0 equiv), K_3_PO_4_ (4.5 equiv), THF, 60 °C, 16 h, microwave irradiation, 54%; (vii) 3,5-bis-trifluoromethylbenzylamine (2.0 equiv), **12** (2.0 equiv), toluene, rt, 21 days, 20% (also isolated [3]rotaxane **11**, 10%).

We demonstrated this by synthesizing [4]rotaxane **13** (Supporting Information, Scheme S2), which contains three different 24-crown-8 derivatives threaded in a single sequence and mechanically maintained in that order, on the thread (Scheme 2). Nitrophenol ester pump **8** was subjected to three pumping cycles, first using dibenzo-24-crown-8 **9** as the macrocycle to give [2]rotaxane **10** (see Supporting Information for synthesis of **13** and intermediates). A pumping cycle on [2]rotaxane **10** with 24-crown-8 (**2**) as the macrocycle then generated [3]rotaxane **11**, and then a third with benzo-24-crown-8 (**12**) afforded [4]rotaxane **13**. Rotaxane **13** was characterized by high-resolution electrospray mass spectrometry (Scheme 2) and ^1^H and ^13^C NMR spectroscopy (Supporting Information, Spectra S47 and S48). [4]Rotaxane **13** was isolated in 2% overall yield (three pumping cycles; an average of 60% per synthetic step) as the only isomer detected out of six possible arrangement of three different macrocycles.

### Synthesis of [5]Rotaxane **16** with Dual-Opening Transamidation Molecular Pump **14**

**Scheme 3:**
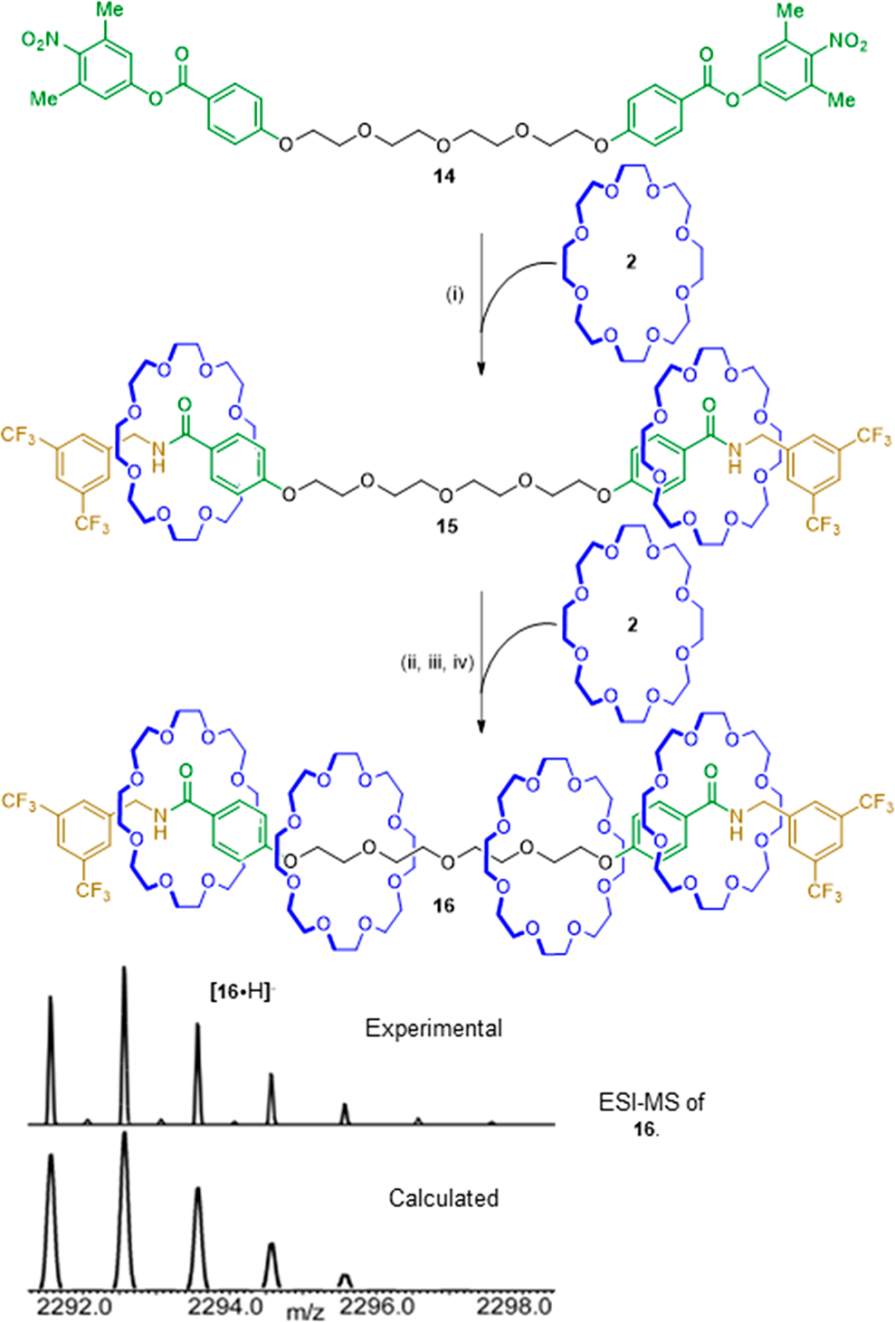
Synthesis of [5]Rotaxane **16** Using a Dual-Opening Molecular Pump Reagents and conditions: (i) 3,5-bis-trifluoromethylbenzylamine (1.0 equiv), **2** (1.0 equiv), toluene, 50 °C, 16 h, 60%; (ii) Boc_2_O (12.0 equiv), DMAP (0.4 equiv), THF, 80 °C, 10 h, microwave irradiation, 80%; (iii) 4-bromo-3,5-dimethylphenol (3.0 equiv), K_3_PO_4_ (4.5 equiv), THF, 60 °C, 16 h, microwave irradiation, 53%; (iv) 3,5-bis-trifluoromethylbenzylamine (2.8 equiv), **2** (5.5 equiv), toluene, rt, 21 days, 35%.

As the “active” end of the thread features a bulky group that inherently prevents dethreading, the transamidation pumping strategy is particularly well suited for operating with pumping motifs at both ends of a thread. We prepared pump **14**, with active esters at either terminus of the catchment region. The design means pump **14** is capable of pumping two macrocycles per transamidation cycle. A bulkier 3,5-dimethyl-4-nitrophenol leaving group was used in **14** to ensure dethreading did not occur en route to [3]rotaxane formation (unsubstituted 4-nitrophenol, the leaving group in **1** and **8**, is not sufficiently bulky to prevent dethreading of **2**). A single pumping cycle on **14** resulted in [3]rotaxane **15** in 60% yield (Scheme 3, step i); a second pumping cycle (Scheme 3, steps ii–iv) gave [5]rotaxane **16** in 9% overall yield from **14**.

**Figure 2:**
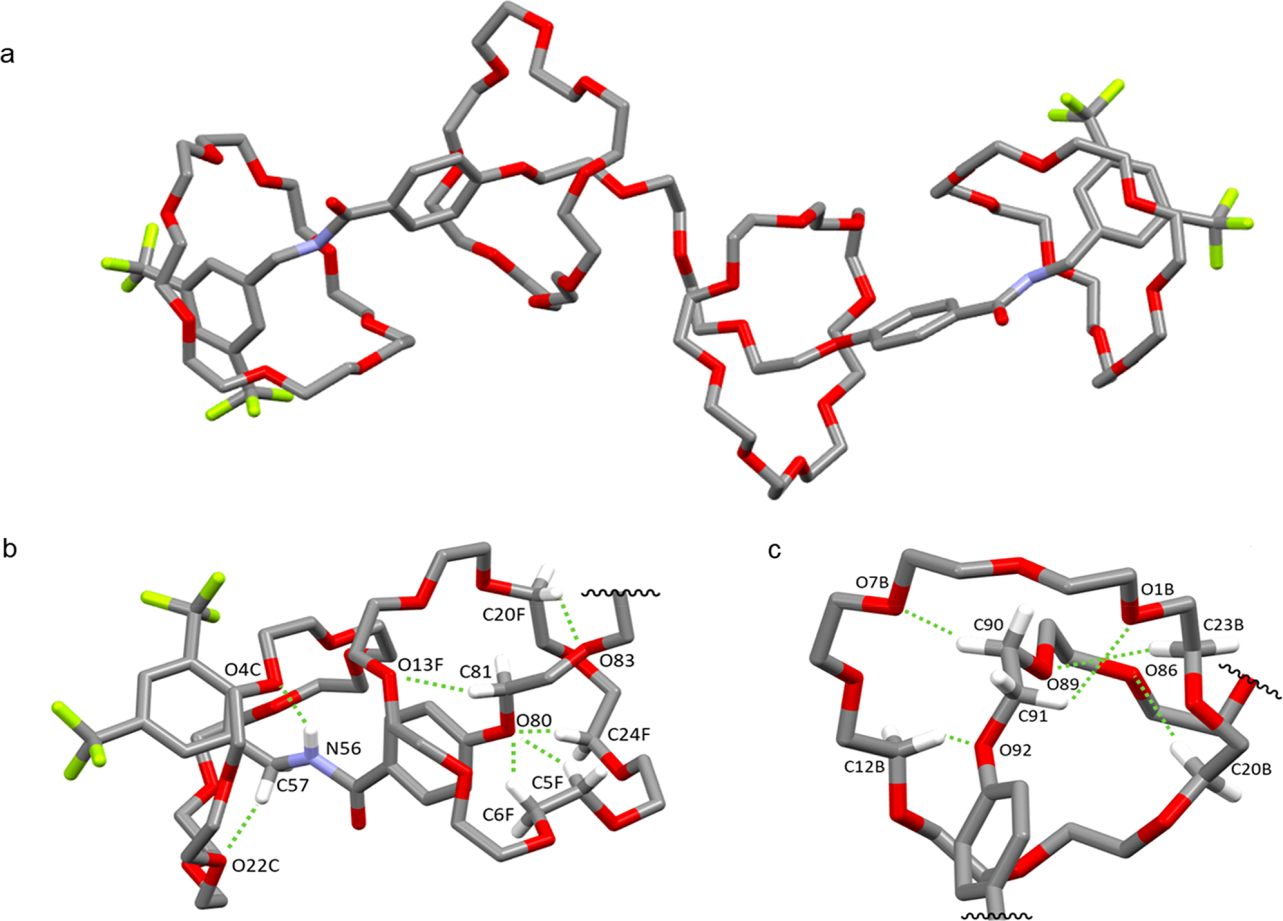
(a) X-ray crystal structure of [5]rotaxane **16**. (b) Expanded view of two macrocycles bound to the amide and on the polyethylene glycol region of the thread, showing hydrogen bond intercomponent CH···O interactions. Hydrogen bond lengths: O4C···HN56, 2.40 Å; O22C···HC57, 2.67 Å; O13F···HC81, 2.51 Å; O80···HC24F, 2.71 Å; O80···HC5F, 2.58 Å; O80···HC6F, 1.99 Å; O83···HC20F, 2.79 Å. Hydrogen bond angles: O4C···H–N56, 151.9°; O22C···H–C57, 148.6°; O80F···H–C24F, 154.8°; O80···H–C5F, 139.3°; O80···H–C6F, 104.9°; O13F···H–C81, 162.3°. (c) View showing CH···O hydrogen bonding of macrocycle on the polyethylene glycol region of the thread. Hydrogen bond lengths: O1B···HC91, 2.56 Å; O7B···HC90, 2.60 Å; O86···HC20B, 2.87 Å; O89···HC23B, 2.57 Å; O92···HC12B, 2.51 Å. Hydrogen bond angles: O1B···H–C91, 117.7°; O7B···H–C90, 114.3°; O86···H–C20B, 161.9°; O89···H–C23B, 152.8°; O92···H–C12B, 131.1°. Carbon, gray; oxygen, red; hydrogen, white; nitrogen, blue; fluorine, yellow. Hydrogen bonds shown in light green. Additional hydrogen atoms and solvent molecules are omitted for clarity.

[5]Rotaxane **16** was characterized by high-resolution electrospray ionization spectrometry (Scheme 3) and ^1^H and ^13^C NMR spectroscopy (Supporting Information, Spectra S61 and S62). Single crystals of **16** suitable for X-ray diffraction were obtained from slow evaporation of a diethyl ether/hexane solution of the rotaxane. The X-ray crystal structure of **16** is shown in Figure 2.

Despite extensive research on crown ethers over the last 50 years,^51^ solid state characterization of complexes between crown ethers and linear oligo(ethylene glycol) chains remains rare.^52^ This is likely a reflection of the lack of driving force for such associations and, perhaps, the tendency of such complexes not to form well-defined single crystals. However, the synthesis of [5]rotaxane **16** does not depend on the thermodynamically favored assembly of a host–guest complex, but rather the crown ethers are driven onto the thread by the information ratchet mechanism and kinetically trapped in the out-of-equilibrium state. The X-ray crystal structure of **16** reveals the weak favorable interactions that the components adopt to achieve a relatively low energy coconformation given their forced association.^53^

The solid state structure of **16** is reminiscent of the coconformation NMR indicates is adopted in CDCl_3_ solution: the two outer macrocycles each bind to a thread amide group through NH···O hydrogen bonding of the amide hydrogen to the crown ether and CH···O=C hydrogen bonding from the crown ether to the amide carbonyl.^28,43,44^ The internal macrocycles do not interact with each other; the system is better stabilized by each forming an extensive array of CH···O interactions with the polyethylene glycol thread, including somewhat unexpectedly the relatively electron poor phenolic oxygens.^54,55^

### The Effectiveness of the Transamidation Pumping Mechanism

The selectivity of crown-ether-stabilized *N*-acylation toward threading over non-interlocked axle formation in [2]rotaxane synthesis (i.e., active template synthesis) was previously found to be >100:1 using 24-crown-8 and nitrophenol ester electrophiles.^43^ In the case of single-opening pumping of **1** to **3** (Scheme 1, step i) or dual-opening pumping of **14** to **15** (Scheme 3, step i), the high selectivity appears to be maintained, and we were not able to isolate any non-interlocked thread (nor [2]rotaxane in the case of Scheme 3, step i) from the crude reaction mixtures. In the pumping to form **6** (Scheme 1, step iv), **11** (Scheme 2, step iv), and **16** (Scheme 3, step iv), when the electrophile is a 4-bromo-3,5-dimethylphenol ester, the active template transamidation is also highly selective with no signals of [2]rotaxane **3**, **10**, or [3]rotaxane **15** observed in the ^1^H NMR of the crude reaction mixtures. The pumping yields are limited by the reactivity of the ester intermediates (**5**, **S11**, and **S17**). In pumping to form [5]rotaxane **16** (Scheme 3, step iv), the potential [4]rotaxane side-product containing two amides (i.e., a product where both esters have reacted but only one macrocycle has threaded) is not observed. In the active template synthesis of **10** from **8** (Scheme 2, step i), where dibenzo-24-crown-8 is the macrocycle rather than 24-crown-8, the selectivity toward [2]rotaxane formation over free thread falls to ∼5:1 (determined by ^1^H NMR of the crude reaction mixture). In the final pumping step to form [4]rotaxane **13**, which uses benzo-24-crown-8 as the macrocycle, the selectivity toward threading decreases further: [3]rotaxane **11** was isolated in 10% yield alongside the [4]rotaxane product (20%). Steric congestion from the rings already trapped on the thread likely contributes to the lower selectivity of threading observed in this pumping step.

## Conclusions

The combination of transamidation active template synthesis and the activation of amides by carboxylation forms a simple and effective stepwise information ratchet mechanism for iteratively pumping multiple crown ethers from bulk solution onto a collection thread. Phenolic esters provide stable rotaxane intermediates in the pumping cycle. Pumps with a single transamidation module sequester one crown ether from bulk solution onto the collection thread per cycle; molecules with transamidation modules at both ends of the thread add two crown ethers per cycle. Pumping does not require the formation of thermodynamically favorable host–guest complexes on regions of the thread nor macrocycle binding sites in the collection region. The X-ray crystal structure of a [5]rotaxane, synthesized using a dual-opening molecular pump, reveals a coconformation stabilized by arrays of weak CH···O interactions. The stepwise operation of transamidation pumps makes it straightforward to synthesize monodispersed oligorotaxanes with a specific number and sequence of different macrocycles. Until recently, the synthesis of rotaxanes required one thread binding site per macrocycle and sequence isomerism in rotaxanes was virtually unknown.^56^ The ability to drive molecular systems directionally away from equilibrium with ratchet mechanisms has ramifications not only for synthesis but for many other aspects of molecular nanotechnology.^7,27,56,57^

## Supporting Information Available

Experimental procedures, synthesis and characterization data, NMR, MS, and X-ray crystallography data (PDF)

## References

[ref1] Skou J. C. The identification of the sodium–potassium pump (Nobel Lecture). Angew. Chem., Int. Ed. 1998, 37, 2320–2328. 10.1002/(SICI)1521-3773(19980918)37:17<2320::AID-ANIE2320>3.0.CO;2-2. 29710959

[ref2] Du D.; Wang-Kan X.; Neuberger A.; van Veen H. W.; Pos K. M.; Piddock L. J. V.; Luisi B. F. Multidrug efflux pumps: structure, function and regulation. Nat. Rev. Microbiol. 2018, 16, 523–539. 10.1038/s41579-018-0048-6. 30002505

[ref3] Oesterhelt D.; Tittor J. Two pumps, one principle: light-driven ion transport in halobacteria. Trends Biochem. Sci. 1989, 14, 57–61. 10.1016/0968-0004(89)90044-3. 2468194

[ref4] Neupane P.; Bhuju S.; Thapa N.; Bhattarai H. K. ATP synthase: structure, function and inhibition. Biomol. Concepts 2019, 10, 1–10. 10.1515/bmc-2019-0001. 30888962

[ref5] Erbas-Cakmak S.; Leigh D. A.; McTernan C. T.; Nussbaumer A. L. Artifical molecular machines. Chem. Rev. 2015, 115, 10081–10206. 10.1021/acs.chemrev.5b00146. 26346838 PMC4585175

[ref6] Stoddart J. F. Mechanically interlocked molecules (MIMs)— molecular shuttles, switches, and machines (Nobel Lecture). Angew. Chem., Int. Ed. 2017, 56, 11094–11125. 10.1002/anie.201703216. 28815900

[ref7] Zhang L.; Marcos V.; Leigh D. A. Molecular machines with bio-inspired mechanisms. Proc. Natl. Acad. Sci. U. S. A. 2018, 115, 9397–9404. 10.1073/pnas.1712788115. 29483259 PMC6156679

[ref8] Qiu Y.; Feng Y.; Guo Q.-H.; Astumian R. D.; Stoddart J. F. Pumps through the ages. Chem. 2020, 6, 1952–1977. 10.1016/j.chempr.2020.07.009.

[ref9] Feng Y.; Ovalle M.; Seale J. S. W.; Lee C. K.; Kim D. J.; Astumian R. D.; Stoddart J. F. Molecular pumps and motors. J. Am. Chem. Soc. 2021, 143, 5569–5591. 10.1021/jacs.0c13388. 33830744

[ref10] Zhou H.-Y.; Zong Q.-S.; Han Y.; Chen C.-F. Recent advances in higher order rotaxane architectures. Chem. Commun. 2020, 56, 9916–9936. 10.1039/D0CC03057K. 32638726

[ref11] Corra S.; Casimiro L.; Baroncini M.; Groppi J.; La Rosa M.; Bakic M. T.; Silvi S.; Credi A. Artificial supramolecular pumps powered by light. Chem.—Eur. J. 2021, 27, 11076–11083. 10.1002/chem.202101163. 33951231 PMC8453702

[ref12] Cheng C.; McGonigal P. R.; Schneebeli S. T.; Li H.; Vermeulen N. A.; Ke C.; Stoddart J. F. An artificial molecular pump. Nat. Nanotechnol. 2015, 10, 547–553. 10.1038/nnano.2015.96. 25984834

[ref13] Erbas-Cakmak S.; Fielden S. D. P.; Karaca U.; Leigh D. A.; McTernan C. T.; Tetlow D. J.; Wilson M. R. Rotary and linear molecular motors driven by pulses of a chemical fuel. Science 2017, 358, 340–343. 10.1126/science.aao1377. 29051374

[ref14] Pezzato C.; Nguyen M. T.; Cheng C.; Kim D. J.; Otley M. T.; Stoddart J. F. An efficient artificial molecular pump. Tetrahedron. 2017, 73, 4849–4857. 10.1016/j.tet.2017.05.087.

[ref15] Pezzato C.; Nguyen M. T.; Kim D. J.; Anamimoghadam O.; Mosca L.; Stoddart J. F. Controlling dual molecular pumps electrochemically. Angew. Chem., Int. Ed. 2018, 57, 9325–9329. 10.1002/anie.201803848. 29774639

[ref16] Qiu Y.; Zhang L.; Pezzato C.; Feng Y.; Li W.; Nguyen M. T.; Cheng C.; Shen D.; Guo Q. H.; Shi Y.; Cai K.; Alsubaie F. M.; Astumian R. D.; Stoddart J. F. A molecular dual pump. J. Am. Chem. Soc. 2019, 141, 17472–17476. 10.1021/jacs.9b08927. 31622089

[ref17] Guo Q.-H.; Qiu Y.; Kuang X.; Liang J.; Feng Y.; Zhang L.; Jiao Y.; Shen D.; Astumian R. D.; Stoddart J. F. Artificial molecular pump operating in response to electricity and light. J. Am. Chem. Soc. 2020, 142, 14443–14449. 10.1021/jacs.0c06663. 32787240

[ref18] Qiu Y.; Song B.; Pezzato C.; Shen D.; Liu W.; Zhang L.; Feng Y.; Guo Q. H.; Cai K.; Li W.; Chen H.; Nguyen M. T.; Shi Y.; Cheng C.; Astumian R. D.; Li X.; Stoddart J. F. A precise polyrotaxane synthesizer. Science 2020, 368, 1247–1253. 10.1126/science.abb3962. 32527831 PMC7375334

[ref19] Amano S.; Fielden S. D. P.; Leigh D. A. A catalysis-driven artificial molecular pump. Nature 2021, 594, 529–534. 10.1038/s41586-021-03575-3. 34163057

[ref20] Feng L.; Qiu Y.; Guo Q.-H.; Chen Z.; Seale J. S.; He K.; Wu H.; Feng Y.; Farha O. K.; Astumian R. D.; Stoddart J. F. Active mechanisorption driven by pumping cassettes. Science 2021, 374, 1215–1221. 10.1126/science.abk1391. 34672694

[ref21] Thomas D.; Tetlow D. J.; Ren Y.; Kassem S.; Karaca U.; Leigh D. A. Pumping between phases with a pulsed-fuel molecular ratchet. Nat. Nanotechnol. 2022, 17, 701–707. 10.1038/s41565-022-01097-1. 35379944

[ref22] Ragazzon G.; Baroncini M.; Silvi S.; Venturi M.; Credi A. Light-powered, artificial molecular pumps: a minimalistic approach. Beilstein J. Nanotechnol. 2015, 6, 2096–2104. 10.3762/bjnano.6.214. 26665081 PMC4660919

[ref23] Ragazzon G.; Baroncini M.; Silvi S.; Venturi M.; Credi A. Light-powered autonomous and directional molecular motion of a dissipative self-assembling system. Nat. Nanotechnol. 2015, 10, 70–75. 10.1038/nnano.2014.260. 25420035

[ref24] Berná J.; Alajarín M.; Orenes R.-A. Azodicarboxamides as template binding motifs for the building of hydrogen-bonded molecular shuttles. J. Am. Chem. Soc. 2010, 132, 10741–10747. 10.1021/ja101151t. 20681706

[ref25] Kay E. R.; Leigh D. A.; Zerbetto F. Synthetic molecular motors and molecular machines. Angew. Chem., Int. Ed. 2007, 46, 72–191. 10.1002/anie.200504313. 17133632

[ref26] Astumian R. D. Irrelevance of the power stroke for the directionality, stopping force, and optimal efficiency of chemically driven molecular machines. Biophys. J. 2015, 108, 291–303. 10.1016/j.bpj.2014.11.3459. 25606678 PMC4302201

[ref27] Aprahamian I. The future of molecular machines. ACS Cent. Sci. 2020, 6, 347–358. 10.1021/acscentsci.0c00064. 32232135 PMC7099591

[ref28] Hannam J. S.; Lacy S. M.; Leigh D. A.; Saiz C. G.; Slawin A. M. Z.; Stitchell S. G. Controlled submolecular translational motion in synthesis: A mechanically interlocking auxiliary. Angew. Chem., Int. Ed. 2004, 43, 3260–3264. 10.1002/anie.200353606. 15213949

[ref29] Fuller A. M. L.; Leigh D. A.; Lusby P. J. One template, multiple rings: controlled iterative addition of macrocycles onto a single binding site rotaxane thread. Angew. Chem., Int. Ed. 2007, 46, 5015–5019. 10.1002/anie.200700933. 17526041

[ref30] Fuller A. M. L.; Leigh D. A.; Lusby P. J. Sequence isomerism in [3]rotaxanes. J. Am. Chem. Soc. 2010, 132, 4954–4959. 10.1021/ja1006838. 20230033

[ref31] Li A.; Tan Z.; Hu Y.; Lu Z.; Yuan J.; Li X.; Xie J.; Zhang J.; Zhu K. Precise control of radial catenane synthesis via clipping and pumping. J. Am. Chem. Soc. 2022, 144, 2085–2089. 10.1021/jacs.1c12303. 35073480

[ref32] Masai H.; Ork Y.; Terao J. Precision synthesis of linear oligorotaxanes and polyrotaxanes achieving well-defined positions and numbers of cyclic components on the axle. Chem. Commun. 2022, 58, 1644–1660. 10.1039/D1CC03507J. 34927653

[ref33] Leigh D. A.; Wong J. K. Y.; Dehez F.; Zerbetto F. Unidirectional rotation in a mechanically interlocked molecular rotor. Nature 2003, 424, 174–179. 10.1038/nature01758. 12853952

[ref34] Hernandez J. V.; Kay E. R.; Leigh D. A. A reversible synthetic rotary molecular motor. Science 2004, 306, 1532–1537. 10.1126/science.1103949. 15567858

[ref35] Serreli V.; Lee C.-F.; Kay E. R.; Leigh D. A. A molecular information ratchet. Nature 2007, 445, 523–527. 10.1038/nature05452. 17268466

[ref36] Astumian R. D. Kinetic asymmetry allows macromolecular catalysts to drive an information ratchet. Nat. Commun. 2019, 10, 3837 10.1038/s41467-019-11402-7. 31444340 PMC6707331

[ref37] Borsley S.; Leigh D. A.; Roberts B. M. W. A doubly kinetically-gated information ratchet autonomously driven by carbodiimide hydration. J. Am. Chem. Soc. 2021, 143, 4414–4420. 10.1021/jacs.1c01172. 33705123

[ref38] Borsley S.; Kreidt E.; Leigh D. A.; Roberts B. M. W. Autonomous fuelled directional rotation about a covalent single bond. Nature 2022, 604, 80–85. 10.1038/s41586-022-04450-5. 35388198

[ref39] Ragazzon G.; Prins L. Energy consumption in chemical fuel-driven self-assembly. Nat. Nanotechnol. 2018, 13, 882–889. 10.1038/s41565-018-0250-8. 30224796

[ref40] Amano S.; Esposito M.; Kreidt E.; Leigh D. A.; Penocchio E.; Roberts B. M. W. Insights from an information thermodynamics analysis of a synthetic molecular motor. Nat. Chem. 2022, 14, 530–537. 10.1038/s41557-022-00899-z. 35301472

[ref41] De Bo G.; Dolphijn G.; McTernan C. T.; Leigh D. A. [2]Rotaxane formation by transition state stabilization. J. Am. Chem. Soc. 2017, 139, 8455–8457. 10.1021/jacs.7b05640. 28621939

[ref42] Fielden S. D. P.; Leigh D. A.; McTernan C. T.; Pérez-Saavedra B.; Vitorica-Yrezabal I. J. Spontaneous assembly of rotaxanes from a primary amine, crown ether and electrophile. J. Am. Chem. Soc. 2018, 140, 6049–6052. 10.1021/jacs.8b03394. 29717609

[ref43] Tian C.; Fielden S. D. P.; Leigh D. A.; Whitehead G. F. S.; Vitorica-Yrezabal I. J. Weak functional group interactions revealed through metal-free active template rotaxane synthesis. Nat. Commun. 2020, 11, 744 10.1038/s41467-020-14576-7. 32029725 PMC7005292

[ref44] Tian C.; Fielden S. D. P.; Pérez-Saavedra B.; Vitorica-Yrezabal I. J.; Leigh D. A. Single-step enantioselective synthesis of mechanically planar chiral [2]rotaxanes using a chiral leaving group strategy. J. Am. Chem. Soc. 2020, 142, 9803–9808. 10.1021/jacs.0c03447. 32356978 PMC7266371

[ref45] Hirose K.; Nishihara K.; Harada N.; Nakamura Y.; Masuda D.; Araki M.; Tobe Y. Highly selective and high-yielding rotaxane synthesis via aminolysis of prerotaxanes consisting of a ring component and a stopper unit. Org. Lett. 2007, 9, 2969–2972. 10.1021/ol070999w. 17616199

[ref46] Lanigan R. M.; Sheppard T. D. Recent developments in amide synthesis: direct amidation of carboxylic acids and transamidation reactions. Eur. J. Org. Chem. 2013, 33, 7453–7465. 10.1002/ejoc.201300573.

[ref47] Acosta-Guzmán P.; Mateus-Gómez A.; Gamba-Sánchez D. Direct transamidation reactions: mechanism and recent advances. Molecules 2018, 23, 2382 10.3390/molecules23092382. PMC6225162 30231486

[ref48] Liu Y.; Shi S.; Achtenhagen M.; Liu R.; Szostak M. Metal-free transamidation of secondary amides via selective N–C cleavage under mild conditions. Org. Lett. 2017, 19, 1614–1617. 10.1021/acs.orglett.7b00429. 28290204

[ref49] Li G.; Szostak M. Highly selective transition-metal-free transamidation of amides and amidation of esters at room temperature. Nat. Commun. 2018, 9, 4165 10.1038/s41467-018-06623-1. 30302003 PMC6178361

[ref50] Lewis J. E. M.; Winn J.; Cera F.; Goldup S. M. Iterative synthesis of oligo[*n*]rotaxanes in excellent yield. J. Am. Chem. Soc. 2016, 138, 16329–16336. 10.1021/jacs.6b08958. 27700073

[ref51] Pedersen C. J. Cyclic polyethers and their complexes with metal salts. J. Am. Chem. Soc. 1967, 89, 2495–2496. 10.1021/ja00986a052.

[ref52] Wu K.-D.; Lin Y.-H.; Chen Lai C.-C.; Chiu S.-H. Na^+^ Ion templated threading of oligo(ethylene glycol) chains through BPX26C6 allows synthesis of [2]rotaxanes under solvent free conditions. Org. Lett. 2014, 16, 1068–1071. 10.1021/ol403602j. 24499390

[ref53] Leigh D. A.; Lusby P. J.; Slawin A. M. Z.; Walker D. B. Rare and diverse binding modes introduced through mechanical bonding. Angew. Chem., Int. Ed. 2005, 44, 4557–4564. 10.1002/anie.200500004. 15973751

[ref54] Steiner T. The hydrogen bond in the solid state. Angew. Chem., Int. Ed. 2002, 41, 48–76. 10.1002/1521-3773(20020104)41:1<48::AID-ANIE48>3.0.CO;2-U. 12491444

[ref55] Steiner T. C–H···O hydrogen bonding in crystals. Crystallogr. Rev. 2010, 9, 177–228. 10.1080/08893110310001621772.

[ref56] Bruns C. J.; Stoddart J. F. The Nature of the Mechanical Bond: From Molecules to Machines; John Wiley & Sons: Hoboken, NJ, 2017.

[ref57] Heard A. W.; Goldup S. M. Simplicity in the design, operation, and applications of mechanically interlocked molecular machines. ACS Cent. Sci. 2020, 6, 117–128. 10.1021/acscentsci.9b01185. 32123730 PMC7047278

