# Supplementary material for: Transamidation-Driven Molecular Pumps
Source: J Am Chem Soc. 2022 Aug 18;144(34):15838–44. doi: 10.1021/jacs.2c06807 (PMC9446885; doi:10.1021/jacs.2c06807)
Supplement: Supplementary file 1 — ja2c06807_si_001.pdf [file ja2c06807_si_001.pdf]

# Transamidation Driven Molecular Pumps

Lorna Binks[‡], Chong Tian[‡], Stephen D. P. Fielden, Iñigo J. Vitorica-Yrezabal and David A. Leigh*

Department of Chemistry, University of Manchester, Oxford Road, Manchester, M13 9PL, UK

*E-mail: david.leigh@manchester.ac.uk

**- Supporting Information -**

# 1 Table of Contents


1 Table of Contents .................... 2

2 General Methods and Abbreviations .................... 4

2 Transamidation screening .................... 5

3 Synthesis of [3]rotaxane **6** .................... 7

3.1 Synthesis of **S1**.................... 8

3.2 Synthesis of **S2**.................... 9

3.3 Synthesis of **S3**.................... 10

3.4 Synthesis of **S4**.................... 11

3.5 Synthesis of **S5**.................... 12

3.6 Synthesis of **1**.................... 13

3.7 Synthesis of **3**.................... 14

3.8 Synthesis of **4**.................... 15

3.9 Synthesis of **5**.................... 16

3.10 Synthesis of **6**.................... 17

3.11 Synthesis of non-interlocked thread **7**.................... 18

4 Synthesis of Benzo-24-crown-8 ether **12**.................... 19

5 Synthesis of [4]rotaxane **13**.................... 20

5.1 Synthesis of **S6**.................... 21

5.2 Synthesis of **S7**.................... 22

5.3 Synthesis of **S8**.................... 23

5.4 Synthesis of **S9**.................... 24

5.5 Synthesis of **8**.................... 25

5.6 Synthesis of **10**.................... 26

5.7 Synthesis of **S10**.................... 27

5.8 Synthesis of **S11**.................... 28

5.9 Synthesis of **11**.................... 29

5.10 Synthesis of **S12**.................... 30

5.11 Synthesis of **S13**.................... 31

5.12 Synthesis of **13**.................... 32

6 Synthesis of [5]rotaxane **16**.................... 33

6.1 Synthesis of **S14**.................... 34

6.2 Synthesis of **S15**.................... 35

6.3 Synthesis of **14**.................... 36

6.4 Synthesis of **15**…… 37

6.5 Synthesis of **S16**…… 38

6.6 Synthesis of **S17**…… 39

6.7 Synthesis of **16**…… 40

8 Single Crystal X-Ray Diffraction…… 41

9 NMR Spectra of New Compounds…… 44

10 References…… 76

# 2 General Methods and Abbreviations

Unless stated otherwise, reagents were obtained from commercial sources and used without purification. Reactions were carried out in anhydrous solvents and under an $N_2$ atmosphere. Anhydrous solvents were obtained by passing the solvent through an activated alumina column on a Phoenix SDS (solvent drying system; JC Meyer Solvent Systems, CA, USA). $^1H$ NMR spectra were recorded on a Bruker Avance III instrument with an Oxford AS600 magnet equipped with a cryoprobe [5mm CPDCH 13C-1H/D] (600 MHz). Chemical shifts are reported in parts per million (ppm) from high to low frequency using the residual solvent peak as the internal reference ($CDCl_3$ = 7.26 ppm, $CD_3OD$ = 3.31 ppm, $(CD_3)_2SO$ = 2.50 ppm and $CD_3CN$ = 1.94 ppm). All $^1H$ resonances are reported to the nearest 0.01 ppm. The multiplicity of $^1H$ signals are indicated as: s = singlet; d = doublet; t = triplet; q = quartet; p = quintet; m = multiplet; br = broad; app = apparent; or combinations of thereof. Coupling constants (*J*) are quoted in Hz and reported to the nearest 0.1 Hz. Where appropriate, averages of the signals from peaks displaying multiplicity were used to calculate the value of the coupling constant. $^{13}C$ NMR spectra were recorded on the same spectrometer with the central resonance of the solvent peak as the internal reference ($CD_3CN$ = 118.26 ppm, $CDCl_3$ = 77.16 ppm, $CD_3OD$ = 49.00 ppm and $(CD_3)_2SO$ = 39.52 ppm). All $^{13}C$ resonances are reported to the nearest 0.01 ppm. DEPT, COSY, HSQC and HMBC experiments were used to aid structural determination and spectral assignment. Fully characterized compounds were chromatographically homogeneous. Flash column chromatography was carried out using Silica 60 Å (particle size 40–63 µm, Sigma Aldrich, UK) as the stationary phase. TLC was performed on precoated silica gel plates (0.25 mm thick, 60 $F_{254}$, Merck, Germany) and visualized using both short and long wave ultraviolet light in combination with standard laboratory stains (basic potassium permanganate, acidic ammonium molybdate and ninhydrin). Microwave irradiation reactions were carried out using a Biotage Initiator$^+$ instrument.

**Abbreviations**: 24-C-8: 24-crown-8 ether; B-24-C-8: benzo-24-crown-8-ether; Boc: *tert*-butoxycarbonyl; COSY: correlated spectroscopy; DB-24-C-8: dibenzyl-24-crown-8-ether; DCM: dichloromethane; DEPT: distortionless enhancement by polarization transfer; *i*-$Pr_2EtN$: *N*,*N*-di-*iso*-propylethylamine; DMAP: 4-dimethylaminopyridine; DMF: dimethylformamide; EDCI: 1-ethyl-3-(3-dimethylaminopropyl)carbodiimide hydrochloride; ESI: electrospray ionization; Et: ethyl; EtOAc: ethyl acetate; h: hour; HMBC: heteronuclear multiple bond correlation; HRMS: high resolution mass spectrometry; HSQC: heteronuclear single quantum coherence; *i*-Pr; isopropyl, LiHMDS: lithium bis(trimethylsilyl)amide; Me: methyl; MW: microwave irradiation; NMR: nuclear magnetic resonance spectroscopy; ppm: parts per million; rt: room temperature; *t*-Bu: tertiary butyl; tert: tertiary; TEA: triethylamine; Tf: trifluoromethanesulfonyl; THF: tetrahydrofuran; TLC: thin layer chromatography; Ts: 4-toluenesulfonyl.

## 2 Transamidation screening

**Table S1**. Screening of conditions to activate amide group of rotaxane **10** (used as a model compound).

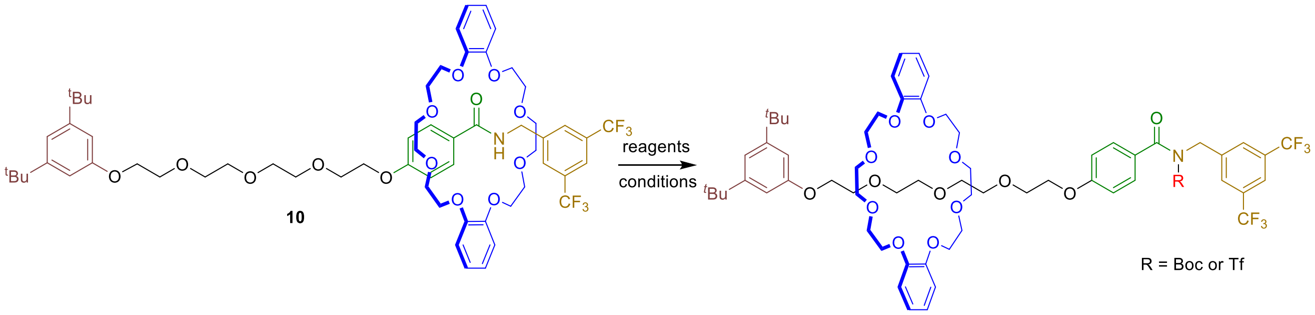


| entry | reagent | conditions | % conversion of **10** to activated amide after 16 h |
|---|---|---|---|
| 1 | $Tf_2O$ | *i*-$Pr_2EtN$, $Et_2O$, −78 °C to rt | 0 |
| 2 | $Tf_2O$ | NaH, toluene, 0 °C to rt | 0 |
| 3 | $Boc_2O$ | DMAP, $CH_2Cl_2$, rt | 0 |
| 4 | $Boc_2O$ | DMAP, THF, 70 °C | 0 |
| 5 | $Boc_2O$ | LiHMDS, DMAP, THF, 0 °C to rt | trace |
| 6 | $Boc_2O$ | DMAP, THF, MW, 90 °C | 77 |

**Table S2**: Screening of phenol nucleophilic catalysts

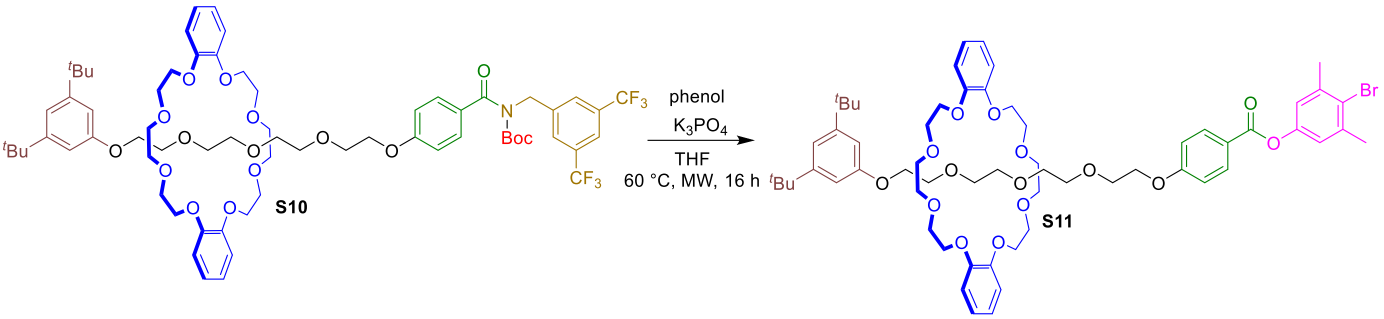


| entry | phenol | % conversion of **S10** to ester after 16 h |
|---|---|---|
| 1 | 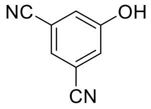 | 0 |
| 2 | 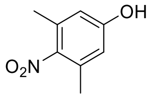 | 0 |
| 3 | 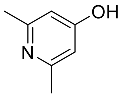 | trace |
| 4 | 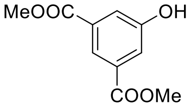 | 15 |
| 6 | 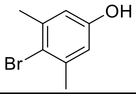 | 68 |

## 3 Synthesis of [3]rotaxane **6**

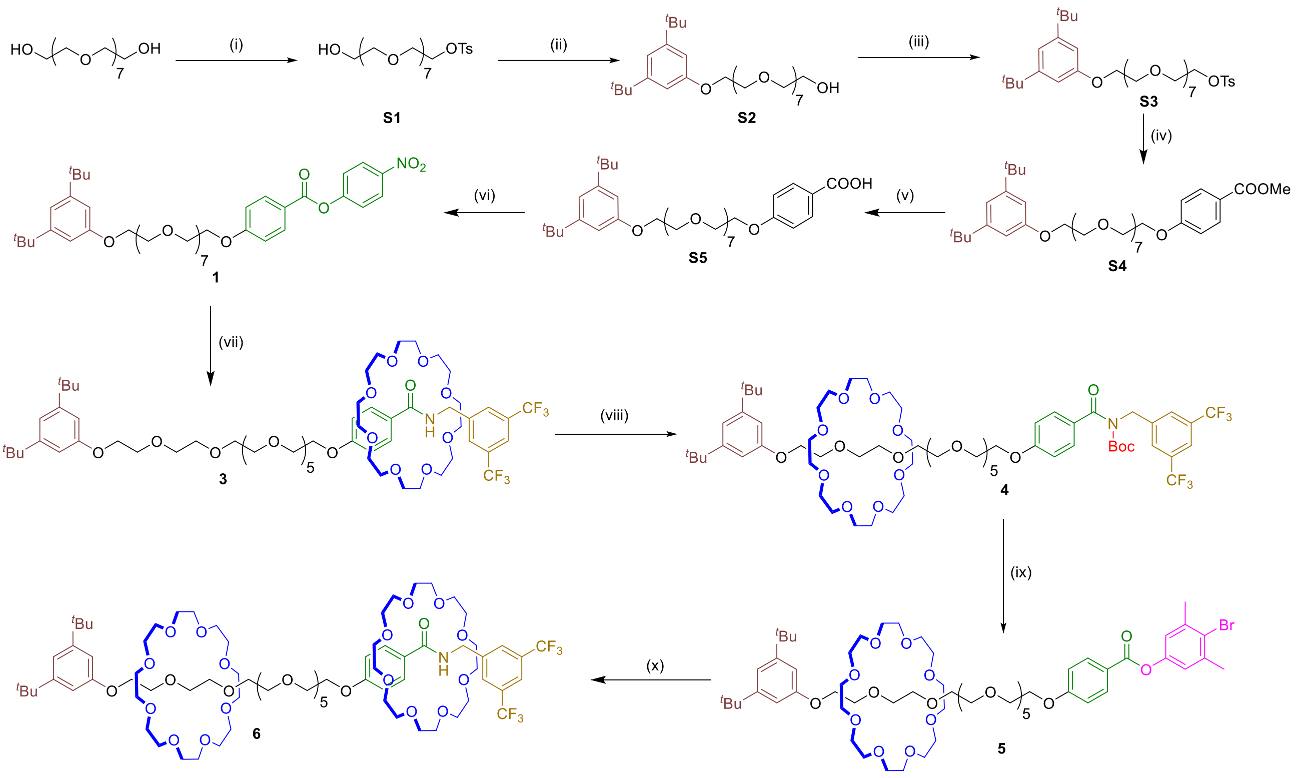

**Scheme S1**. Conditions: (i) TsCl, $Et_3N$, $CH_2Cl_2$, rt, 16 h, 76%. (ii) 3,5-di-*tert*-butyl-phenol, $K_2CO_3$, DMF, 80 °C, 16 h, 61%. (iii) TsCl, $Et_3N$, $CH_2Cl_2$, rt, 16 h, 90%. (iv) methyl paraben, $K_2CO_3$, DMF, 80 °C, 16 h, 83%. (v) NaOH (5 % in $H_2O$), MeOH, 60 °C, 16 h, 90% (vi) 4-nitro-phenol, EDCI, DMAP, $CH_2Cl_2$, rt, 16 h, 85%. (vii) 3,5-bis-trifluoromethylbenzylamine, 24-C-8 **2**, toluene, rt, 16 h, 65%. (viii) $Boc_2O$, DMAP, THF, 90 °C, 10 h, MW, 77%. (ix) 4-bromo-3,5-dimethyl phenol, $K_3PO_4$, THF, 60 °C, 16 h, MW, 68%. (x) 3,5-bis-trifluoromethylbenzylamine, 24-C-8 **2**, toluene, rt, 10 days, 50%.

## 3.1 Synthesis of **S1**

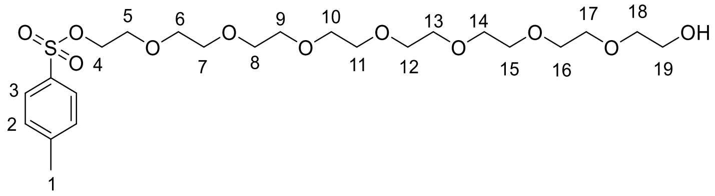


To a stirring solution of octaethylene glycol (5.00 g, 14 mmol, 2.0 eq.) in $CH_2Cl_2$ (20 ml) were added $Et_3N$ (1.25 ml, 9.0 mmol, 1.3 eq.) and *p*-toluenesulfonyl chloride (1.28 g, 6.8 mmol, 1.0 eq.). The reaction mixture was stirred at room temperature for 16 hours after which the solution was concentrated and the residue purified by flash chromatography ($SiO_2$ $CH_2Cl_2$/ MeOH 30:1) to afford **S1** as a colorless oil (2.80 g, 5.1 mmol, 76%).

**$^1$H NMR** (600 MHz, $CDCl_3$) δ 7.73 (d, *J* = 8.44 Hz, 2H, $H_3$), 7.27 (d, *J* = 8.18 Hz, 2H, $H_2$), 4.08 (t, *J* = 4.60 Hz, 2H, $H_4$), 3.72 (t, *J* = 4.66 Hz, 2H, $H_{18}$), 3.61 (t, *J* = 4.88 Hz, 2H, $H_5$), 3.60 – 3.58 (m, 8H, $H_{6, 7, 16, 17}$), 3.58 (s, 4H, $H_{8, 15}$), 3.57 (s, 4H, $H_{9, 14}$), 3.55 (s, 4H, $H_{10, 13}$), 3.53 (br, 2H, $H_{19}$), 3.59 (s, 4H, $H_{11, 12}$), 2.38 (s, 3H, $H_1$).

**$^{13}$C NMR** (151 MHz, $CDCl_3$) δ 144.9, 133.1, 130.0, 128.1, 72.7, 70.9, 70.73, 70.72, 70.67 (5C), 70.65, 70.64, 70.63, 70.4, 69.27, 68.70, 61.73, 21.67.

**HRMS** (ESI$^+$) Calculated for $C_{23}H_{40}O_{11}NaS$ $[M+Na]^+$ 547.2184 found 547.2176.

## 3.2 Synthesis of **S2**

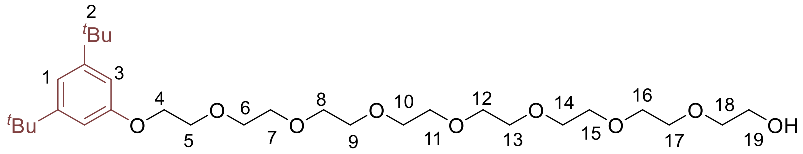

**S1** (2.80 g, 5.1 mmol, 1.0 eq.) and 3,5-di-*tert*-butyl-phenol (1.05 g, 5.1 mmol, 1.0 eq.) were dissolved in DMF (10 ml). To this mixture was added $K_2CO_3$ (0.71 g, 5.1 mmol, 1.0 eq.). The reaction was stirred at 80 °C for 16 hours. The solvent was removed under reduced pressure and the residue purified by flash column chromatography ($SiO_2$, EtOAc/Hexane 3:1) to afford **S2** as a colorless oil (1.80 g, 3.1 mmol, 61%).

**$^1$H NMR** (600 MHz, $CDCl_3$) δ 7.04 (s, 1H, $H_1$), 6.79 (s, 2H, $H_3$), 4.18 (t, $J$ = 4.99 Hz, 2H, $H_4$), 3.89 (t, $J$ = 5.16 Hz, 2H, $H_5$), 3.76 – 3.73 (m, 4H, $H_{18, 17}$), 3.71 – 3.66 (m, 22H, $H_{6, 7, 8, 9, 10, 11, 12, 13, 14, 15, 16}$), 3.63 (t, $J$ = 4.66 Hz, 2H, $H_{19}$), 1.32 (s, 18H, $H_2$).

**$^{13}$C NMR** (151 MHz, $CDCl_3$) δ 158.4, 152.3, 115.2, 109.1, 72.6, 70.9, 70.77, 70.75, 70.74, 70.72-70.67 (6C), 70.66, 70.5, 70.0, 67.3, 61.9, 35.1, 31.6.

**HRMS** (ESI$^+$) Calculated for $C_{30}H_{54}O_9Na$ $[M+Na]^+$ 581.3660 found 581.3664.

## 3.3 Synthesis of **S3**

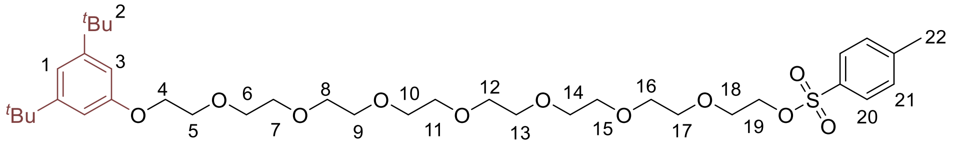


To a stirring solution of **S2** (1.80 g, 2.5 mmol, 1.0 eq.) in $CH_2Cl_2$ (20 ml) were added $Et_3N$ (0.37 ml, 2.6 mmol, 1.1 eq.) and *p*-toluenesulfonyl chloride (0.47 g, 2.5 mmol, 1.0 eq.). The reaction mixture was stirred at room temperature for 16 hours after which the solution was concentrated and the residue purified by flash chromatography ($SiO_2$, $CH_2Cl_2$/ MeOH 30:1) to afford **S3** as a colorless oil (1.62 g, 2.2 mmol, 90%).

**$^1$H NMR** (500 MHz, $CDCl_3$) δ 7.72 (d, *J* = 8.18 Hz, 2H, $H_{20}$), 7.27 (d, *J* = 8.08 Hz, 2H, $H_{21}$), 6.95 (s, 1H, $H_1$), 6.70 (s, 2H, $H_3$), 4.14 – 4.06 (m, 4H, $H_{4, 19}$), 3.79 (t, *J* = 5.04 Hz, 2H, $H_{18}$), 3.66 (t, *J* = 4.45 Hz, 2H, $H_{15}$), 3.61 (t, *J* = 5.20 Hz, 4H, $H_{6, 17}$), 3.58 (br, 4H, $H_{7, 16}$), 3.57 (br, 4H, $H_{8, 15}$), 3.56 (br, 4H, $H_{9, 14}$), 3.54 (br, 4H, $H_{10, 13}$), 3.51 (br, 4H, $H_{11, 12}$), 2.37 (s, 3H, $H_{22}$), 1.24 (s, 18H, $H_2$).

**$^{13}$C NMR** (126 MHz, $CDCl_3$) δ 158.4, 152.3, 144.9, 133.1, 130.0, 128.1, 115.2, 109.1, 70.90, 70.87, 70.8 (2C), 70.73, 70.69 (6C), 70.6, 70.0, 69.4, 68.8, 67.3, 35.1, 31.6, 21.8.

**HRMS** (ESI$^+$) Calculated for $C_{37}H_{60}O_{11}NaS$ $[M+Na]^+$ 735.3749 found 735.3733.

## 3.4 Synthesis of **S4**

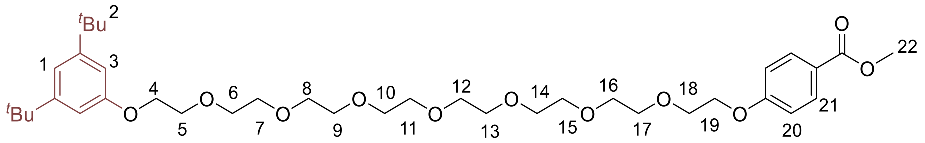


**S3** (1.62 g, 2.2 mmol, 1.0 eq.) and methyl paraben (0.33 g, 2.2 mmol, 1.0 eq.) were dissolved in DMF (10 ml). To this mixture was added $K_2CO_3$ (0.30 g, 2.2 mmol, 1.0 eq.). The reaction was stirred at 80 °C for 16 hours. The solvent was removed under reduced pressure and the residue purified by flash column chromatography ($SiO_2$, EtOAc/Hexane 3:1) to afford **S4** as a colorless oil (1.31 g, 1.8 mmol, 83%).

**$^1$H NMR** (600 MHz, $CDCl_3$) δ 8.00 (d, *J* = 8.07 Hz, 2H, $H_{21}$), 7.03 (s, 1H, $H_1$), 6.95 (d, *J* = 8.76 Hz, 2H, $H_{20}$), 6.79 (d, *J* = 1.44 Hz, 2H, $H_3$), 4.20 (t, *J* = 4.71 Hz, 2H, $H_{19}$), 4.16 (t, *J* = 4.91 Hz, 2H, $H_4$), 3.90 (s, 3H, $H_{22}$), 3.89 – 3.86 (m, 4H, $H_{5,18}$), 3.76 – 3.73 (m, 4H, $H_{6, 17}$), 3.71 – 3.68 (m, 4H, $H_{7, 16}$), 3.68 – 3.66 (m, 8H, $H_{8, 9, 14, 15}$), 3.52 (s, 8H, $H_{10, 11, 12, 13}$), 1.32 (s, 18H, $H_2$).

**$^{13}$C NMR** (151 MHz, $CDCl_3$) δ $^{13}$C NMR 167.0, 162.7, 158.5, 152.3, 131.7, 122.8, 115.2, 114.3, 109.1, 71.0, 70.9, 70.8 (4C), 70.7 (6C), 70.0, 69.7, 67.70, 67.3, 52.0, 35.1, 31.6.

**HRMS** (ESI$^+$) Calculated for $C_{38}H_{60}O_{11}Na$ $[M+Na]^+$ 715.4028 found 715.4025.

## 3.5 Synthesis of **S5**

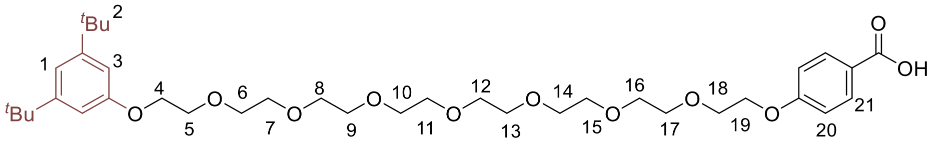


To a solution of **S4** (1.31 g, 1.8 mmol, 1.0 eq.) in MeOH (4.30 mL) was added NaOH aqueous solution (5% w/v, 3.40 mL). The reaction mixture was stirred at 60 °C for 16 hours. The solvent was removed under reduced pressure and the residue was purified by flash column chromatography ($SiO_2$, $CH_2Cl_2$/MeOH 25:1) to afford **S5** as a colorless oil (1.15 g, 1.6 mmol, 90%).

**$^1$H NMR** (500 MHz, $CDCl_3$) δ 8.06 (d, *J* = 8.80 Hz, 2H, $H_{21}$), 7.04 (s, 1H, $H_1$), 6.98 (d, *J* = 8.79 Hz, 2H, $H_{20}$), 6.80 (s, 2H, $H_3$), 4.22 (t, *J* = 4.46 Hz, 2H, $H_{19}$), 4.16 (t, *J* = 4.80 Hz, 2H, $H_4$), 3.91 – 3.86 (m, 4H, $H_{5, 18}$), 3.77 – 3.73 (m, 4H, $H_{6, 17}$), 3.72 – 3.62 (m, 4H, $H_{7, 16}$), 3.68 – 3.62 (m, 16H, $H_{8, 9, 10, 11, 12, 13, 14, 15}$), 1.32 (s, 18H, $H_2$).

**$^{13}$C NMR** (126 MHz, $CDCl_3$) δ $^{13}$C NMR (126 MHz, $CDCl_3$) δ 170.7, 163.3, 158.4, 152.3, 132.4, 122.0, 115.2, 114.5, 109.1, 71.0, 70.9, 70.78, 70.77 (2C), 70.74, 70.69 (6C), 70.0, 69.7, 67.8, 67.3, 35.1, 31.6.

**HRMS** (ESI$^+$) Calculated for $C_{37}H_{58}O_{11}Na$ $[M+Na]^+$ 701.3871 found 701.3852.

## 3.6 Synthesis of **1**

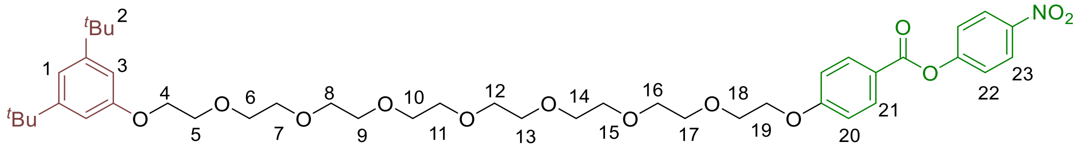

To a solution of **S5** (1.15 g, 2.1 mmol, 1.0 eq.) in $CH_2Cl_2$(15 mL) were added 4-nitrophenol (0.35 g, 2.6 mmol, 1.2 eq.), DMAP (0.26 g, 2.1 mmol, 1.0 eq) and EDCI (0.40 g, 2.6 mmol, 1.2 eq.). The reaction mixture was stirred for 16 hours at room temperature. The solvent was removed under reduced pressure and the residue purified by flash column chromatography ($SiO_2$, Hexane/ EtOAc 2:1) to afford **1** as a colorless oil (1.48 g, 1.8 mmol, 85%).

**$^1$H NMR** (500 MHz, $CDCl_3$) δ 8.35 (d, *J* = 9.08 Hz, 2H, $H_{23}$), 8.16 (d, *J* = 8.56 Hz, 2H, $H_{21}$), 7.42 (d, *J* = 9.16 Hz, 2H, $H_{22}$), 7.05 (d, *J* = 7.55 Hz, 2H, $H_{20}$), 7.03 (s, 1H, $H_1$), 6.79 (s, 2H, $H_3$), 4.24 (t, *J* = 4.05 Hz, 2H, $H_{19}$), 4.16 (t, *J* = 4.55 Hz, 2H, $H_4$), 3.93 (t, *J* = 5.05 Hz, 2H, $H_{18}$), 3.88 (t, *J* = 4.67 Hz, 2H, $H_5$), 3.77 – 3.74 (m, 4H, $H_{6, 17}$), 3.72 – 3.69 (m, 4H, $H_{7, 16}$), 3.68 (s, 8H, $H_{8, 9, 14, 15}$), 3.67 (s, 8H, $H_{10, 11, 12, 13}$), 1.32 (s, 18H, $H_2$).

**$^{13}$C NMR** (126 MHz, $CDCl_3$) δ 164.1, 163.8, 158.5, 156.1, 152.3, 145.4, 132.7, 125.4, 122.8, 121.0, 115.3, 114.8, 109.1, 71.1, 70.9, 70.8 (4C), 70.7 (6C), 70.0, 69.6, 67.9, 67.3, 35.1, 31.6.

**HRMS** (ESI$^+$) Calculated for $C_{43}H_{61}O_{13}NNa$ $[M+Na]^+$ 822.4035 found 822.4009.

## 3.7 Synthesis of **3**

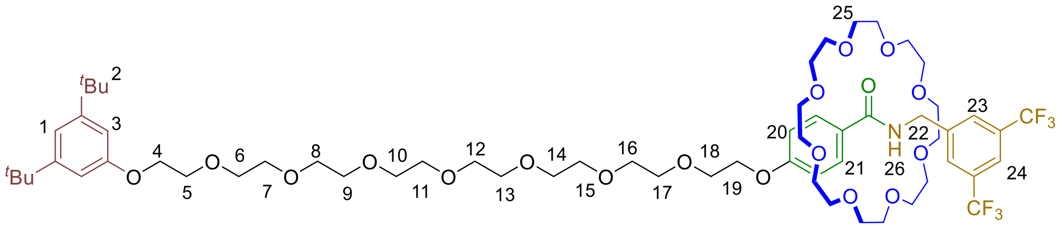

To a solution of **1** (1.48 g, 1.8 mmol, 1.0 eq.) in toluene (10 mL) were added 24-C-8 **2** (0.63 g, 1.8 mmol, 1.0 eq.) and 3,5-bis-trifluoromethylbenzylamine (0.44 g, 1.8 mmol, 1.0 eq.). The reaction mixture was stirred at room temperature for 16 hours. The solvent was removed under reduced pressure and the residue was purified by flash column chromatography ($SiO_2$, $CH_2Cl_2$/MeOH 25:1) to afford **3** as a colorless oil (1.40 g, 1.1 mmol, 65%).

**$^1$H NMR** (600 MHz, $CDCl_3$) δ 8.72 (s, 2H, $H_{23}$), 8.01 (d, *J* = 8.51 Hz, 2H, $H_{21}$), 7.81 (br, 1H, $H_{26}$), 7.75 (s, 1H, $H_{24}$), 7.03 (s, 1H, $H_1$), 6.87 (d, *J* = 9.17 Hz, 2H, $H_{20}$), 6.79 (s, 2H, $H_3$), 4.94 (s, 2H, $H_{22}$), 4.15 (t, *J* = 4.25 Hz, 4H, $H_{4,19}$), 3.88 (t, *J* = 4.69 Hz, 4H, $H_{5,18}$), 3.76 – 3.73 (m, 4H, $H_{6,17}$), 3.71 – 3.68 (m, 4H, $H_{7,16}$), 3.67 (s, 8H, $H_{8,9,14,15}$), 3.66 (s, 8H, $H_{10,11,12,13}$), 3.42 (q, *J* = 6.57 Hz, 16H, $H_{25}$), 3.19 (q, *J* = 6.15 Hz, 16H, $H_{25}$), 1.32 (s, 18H, $H_2$).

**$^{13}$C NMR** (151 MHz, $CDCl_3$) δ 166.9, 160.8, 158.4, 152.3, 141.3, 135.5, 130.0, 129.1 (q, *J* = 32.8 Hz), 128.2, 124.3 (q, *J* = 272.4 Hz), 119.8 (m), 115.2, 113.6, 109.1, 71.0, 70.9, 70.8 (3C), 70.74, 70.70 (6C), 70.6, 70.0, 69.8, 67.5, 67.3, 44.7, 35.1, 31.6.

**HRMS** (ESI$^+$) Calculated for $C_{62}H_{95}O_{18}NF_6Na$ $[M+Na]^+$ 1278.6346 found 1278.6341.

## 3.8 Synthesis of **4**

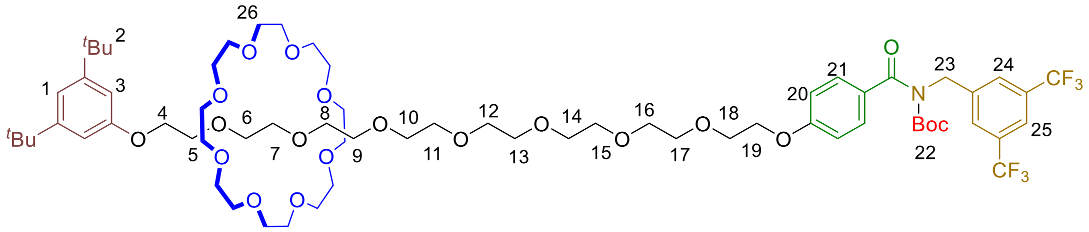


To a THF (4 ml) solution of **3** (1.40 g, 1.1 mmol, 1.0 eq.) in a microwave vial were added DMAP (27.0 mg, 0.22 mmol, 0.2 eq.) and $Boc_2O$ (1.45 g, 6.7 mmol, 6.0 eq.) in THF (4 ml). The reaction mixture was sealed and stirred under microwave irradiation at 90 °C for 10 hours. The mixture was cooled to room temperature and the solvent was removed under reduced pressure. Purification of the residue by flash column chromatography ($SiO_2$, $CH_2Cl_2$/MeOH 30:1) afforded **4** as a colorless oil (1.20 g, 0.87 mmol, 77%).

**$^1$H NMR** (600 MHz, $CDCl_3$) δ 7.82 (s, 2H, $H_{24}$), 7.72 (s, 1H, $H_{25}$), 7.48 (d, $J$ = 8.62 Hz, 2H, $H_{21}$), 7.08 (d, $J$ = 8.26 Hz, 2H, $H_{20}$), 6.93 (s, 1H, $H_1$), 6.74 (s, 2H, $H_3$), 4.96 (s, 2H, $H_{23}$), 4.38 (br, 2H, $H_{19}$), 4.11 (t, $J$ = 4.96 Hz, 2H, $H_4$), 3.88 (t, $J$ = 4.87 Hz, 2H, $H_{18}$), 3.80 (t, $J$ = 5.00 Hz, 2H, $H_5$), 3.68 – 3.65 (m, 4H, $H_{6, 17}$), 3.63 – 3.60 (m, 4H, $H_{7, 16}$), 3.58 (s, 16H, $H_{8, 9, 10, 11, 12, 13, 14, 15}$), 3.54 – 3.43 (m, 32H, $H_{26}$), 1.23 (s, 18H, $H_2$), 1.13 (s, 9H, $H_{22}$).

**$^{13}$C NMR** (126 MHz, $CDCl_3$) δ $^{13}$C NMR (126 MHz, $CDCl_3$) δ 172.8, 163.0, 158.6, 153.6, 152.1, 140.9, 131.8 (q, $J$ = 33.2 Hz), 130.2, 128.5 (q, $J$ = 4.1 Hz), 127.8, 123.4 (q, $J$ = 272.6 Hz), 121.5 (m), 115.1, 115.0, 109.3, 83.6, 70.74 (3C), 70.69 (3C), 70. 7 (4C), 70.6, 70.4, 70.3, 70.1, 69.7, 67.5, 67.1, 48.6, 35.1, 31.6, 27.6.

**HRMS** (ESI$^+$) Calculated for $C_{67}H_{103}O_{20}NF_6Na$ $[M+Na]^+$ 1378.6870 found 1378.6858.

## 3.9 Synthesis of **5**

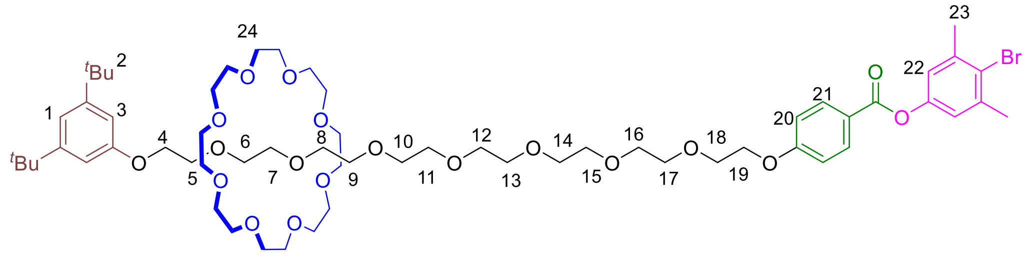

To a THF (2 ml) solution of **4** (1.40 g, 1.0 mmol, 1.0 eq.) in a microwave vial were added 4-bromo-3,5-dimethyl phenol (0.20 g, 1.0 mmol, 1.0 eq.) and $K_3PO_4$ (0.32 g, 1.5 mmol, 1.5 eq.). The reaction mixture was sealed and stirred under microwave irradiation at 60 °C for 16 hours. The mixture was cooled to room temperature and the solvent was removed under reduced pressure. Purification by flash column chromatography ($SiO_2$, $CH_2Cl_2$/MeOH 40:1) yielded **5** as a colorless oil (0.85 g, 0.68 mmol, 68%).

**$^1$H NMR** (600 MHz, $CDCl_3$) δ 8.12 (d, *J* = 8.17 Hz, 2H, $H_{21}$), 7.31 (d, *J* = 8.17 Hz, 2H, $H_{20}$), 7.03 (s, 1H, $H_1$), 6.98 (s, 2H, $H_{22}$), 6.82 (s, 2H, $H_3$), 4.56 (t, *J* = 5.24 Hz, 2H, $H_{19}$), 4.20 (t, *J* = 4.47 Hz, 2H, $H_4$), 4.00 (t, *J* = 5.59 Hz, 2H, $H_{18}$), 3.89 (t, *J* = 4.61 Hz, 2H, $H_5$), 3.77 – 3.75 (m, 4H, $H_{6, 17}$), 3.72 – 3.70 (m, 4H, $H_{7, 16}$), 3.68 (m, 8H, $H_{8, 9, 14, 15}$), 3.68 (m, 8H, $H_{10, 11, 12, 13}$), 3.64 – 3.58 (m, 16H, $H_{24}$), 3.56 – 3.51 (m, 16H, $H_{24}$), 2.45 (s, 6H, $H_{23}$), 1.33 (s, 18H, $H_2$).

**$^{13}$C NMR** (151 MHz, $CDCl_3$) δ 165.5, 164.3, 158.5, 152.2, 149.7, 139.6, 132.0, 124.1, 121.7, 120.6, 115.8, 115.1, 109.3, 70.80 (2C), 70.76, 70.73 (2C), 70.70 (5C), 70.66, 70.4, 70.2, 70.1, 69.9, 67.5, 67.2, 35.1, 31.6, 24.1.

**HRMS** (ESI$^+$) Calculated for $C_{61}H_{97}O_{19}BrNa$ $[M+Na]^+$ 1235.5700 found 1235.5718.

## 3.10 Synthesis of **6**

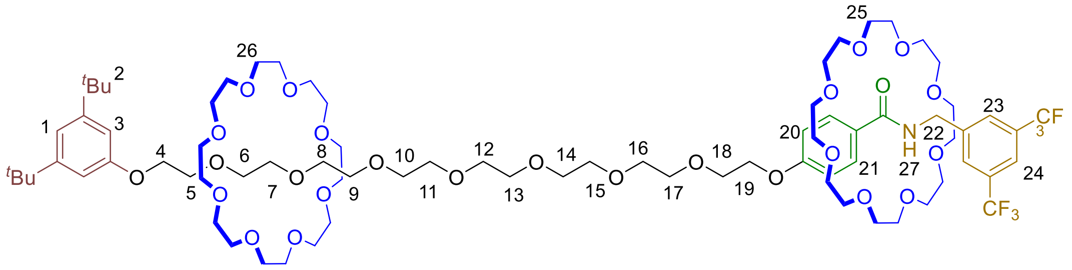


To a solution of **5** (0.12 g, 0.10 mmol, 1.0 eq.) in toluene (2.5 mL) were added 24-C-8 **2** (0.63 g, 0.19 mmol, 2.0 eq.) and 3,5-bis-trifluoromethylbenzylamine (0.047 g, 0.19 mmol, 2.0 eq.). The reaction mixture was stirred at room temperature for 10 days. The solvent was removed under reduced pressure. Purification of the residue by flash column chromatography ($SiO_2$, $CH_2Cl_2$/MeOH 25:1) afforded **6** as a colorless oil (0.079 g, 0.048 mmol, 50%).

**$^1$H NMR** (600 MHz, $CDCl_3$) δ 8.74 (s, 2H, $H_{23}$), 7.98 (d, $J$ = 9.15 Hz, 2H, $H_{21}$), 7.78 (br, s, 1H, $H_{27}$), 7.74 (s, 1H, $H_{24}$), 7.01 (s, 1H, $H_1$), 7.00 (d, $J$ = 9.05 Hz, 2H, $H_{20}$), 6.85 (s, 2H, $H_3$), 4.93 (d, $J$ = 3.89 Hz, 2H, $H_{22}$), 4.33 (t, $J$ = 4.78 Hz, 2H, $H_{19}$), 4.24 (t, $J$ = 4.96 Hz, 2H, $H_4$), 3.95 (t, $J$ = 4.65 Hz, 2H, $H_{18}$), 3.91 (t, $J$ = 5.54 Hz, 2H, $H_5$), 3.77 – 3.74 (m, 4H, $H_{6, 17}$), 3.69 – 3.66 (m, 4H, $H_{7, 16}$), 3.64 (s, 16H, $H_{8, 9, 10, 11, 12, 13, 14, 15}$), 3.59 – 3.57 (m, 32H, $H_{26}$), 3.43 (q, $J$ = 6.34 Hz, 16H, $H_{25}$), 3.19 (q, $J$ = 6.35 Hz, 16H, $H_{25}$), 1.32 (s, 18H, $H_2$).

**$^{13}$C NMR** (151 MHz, $CDCl_3$) δ 167.0, 161.1, 158.6, 152.0, 141.6, 135.6, 129.6, 129.0 (q, $J$ = 32.4 Hz), 128.0, 124.3 (q, $J$ = 272.6 Hz), 119.7, 114.8, 114.1, 109.4, 70.71 (2C), 70.66 (2C), 70.64 (6C), 70.59, 70.56, 70.4, 70.3, 70.1, 69.8, 67.3, 67.0, 44.6, 35.1, 31.6.

**HRMS** (ESI$^+$) Calculated for $C_{78}H_{127}O_{26}NF_6Na$ $[M+Na]^+$ 1630.8443 found 1630.8384.

## 3.11 Synthesis of non-interlocked thread **7**

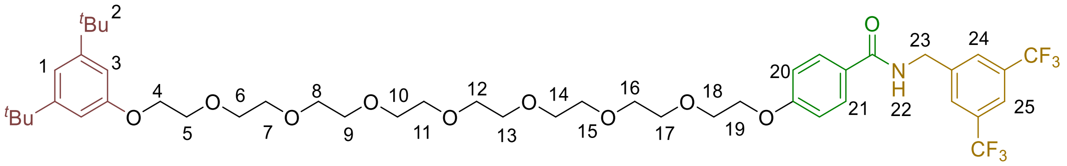

To a solution of **S5** (0.070 g, 0.11 mmol, 1.0 eq.) in $CH_2Cl_2$ (3 mL) were added 3,5-bis-trifluoromethylbenzylamine (0.030 g, 0.13 mmol, 1.2 eq.), DMAP (0.020 g, 0.13 mmol, 1.2 eq.) and EDCI (0.03 g, 0.13 mmol, 1.2 eq.). The reaction mixture was stirred for 16 hours at room temperature. The solvent was removed under reduced pressure and the residue was purified by flash column chromatography ($SiO_2$, Hexane/EtOAc 2:1) to afford **7** as a colorless oil (0.080 g, 0.080 mmol, 78%).

**$^1$H NMR** (500 MHz, $CDCl_3$) δ 7.81 – 7.77 (m, 4H, $H_{20, 24}$), 7.00 (s, 1H, $H_1$), 6.96 (d, *J* = 8.70 Hz, 2H, $H_{21}$), 6.75 (s, 3H, $H_{3, 22}$), 4.24 (d, *J* = 5.94 Hz, 2H, $H_{23}$), 4.17 (t, *J* = 4.62 Hz, 2H, $H_4$), 4.11 (t, *J* = 5.12 Hz, 2H, $H_{19}$), 3.86 (t, *J* = 4.99 Hz, 2H, $H_{18}$), 3.83 (t, *J* = 5.49 Hz, 2H, $H_5$), 3.72 – 3.69 (m, 4H, $H_{6, 17}$), 3.66 – 3.64 (m, 4H, $H_{7, 16}$), 3.63 – 3.60 (s, 8H, $H_{8, 9, 14, 15}$), 3.60 (s, 8H, $H_{10, 11, 12, 13}$), 1.29 (s, 18H, $H_2$).

**$^{13}$C NMR** (126 MHz, $CDCl_3$) δ 167.2, 161.9, 158.4, 152.3, 141.7, 132.0 (q, *J* = 33.3 Hz), 129.1, 128.1, 126.0, 123.3 (d, *J* = 272.1 Hz), 121.5, 115.3, 114.8, 109.1, 71.3, 70.9, 70.8 (4C), 70.7 (6C), 70.0, 69.6, 67.8, 67.2, 42.9, 35.1, 31.6.

**HRMS** (ESI$^+$) Calculated for $C_{46}H_{63}O_{10}NF_6Na$ $[M+Na]^+$ 926.4248 found 926.4203.

## 4 Synthesis of Benzo-24-crown-8 ether **12**

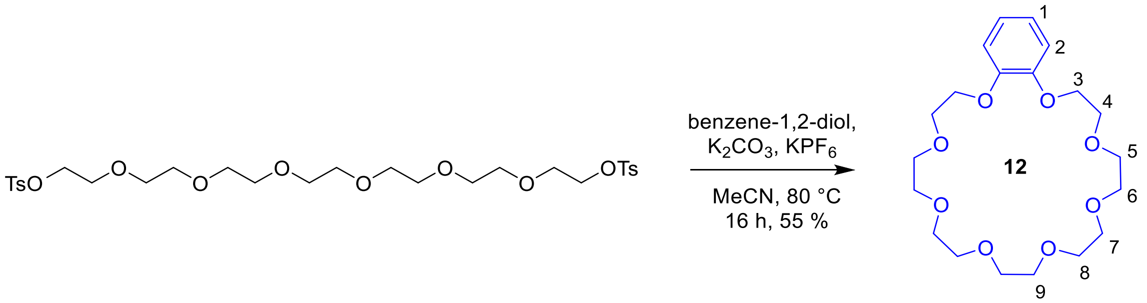


To a solution of heptaethylene glycol di(p-toluenesulfonate) (0.35 g, 0.55 mmol, 1.0 eq.) in MeCN (5 ml) were added benzene-1,2-diol (0.060 g, 0.55 mmol, 1.0 eq.), $K_2CO_3$ (0.23 g, 1.7 mmol, 3.0 eq.) and $KPF_6$ (0.31 g, 1.7 mmol, 3.0 eq.). The reaction was stirred at 80 °C for 16 hours, after which the reaction was concentrated to dryness and washed with water (50 ml) and extracted with ethyl acetate (3 x 50 ml). Purification of the residue by flash column chromatography ($SiO_2$, $CH_2Cl_2$/MeOH 25:1) afforded **12** as a colorless oil (0.20 g, 0.47 mmol, 55%).

**$^1$H NMR** (600 MHz, $CDCl_3$) δ 6.91 (s, 4H, $H_{1,2}$), 4.17 (t, *J* = 4.24 Hz, 4H, $H_3$), 3.91 (t, *J* = 4.65 Hz, 4H, $H_4$), 3.80 (t, *J* = 4.10 Hz, 4H, $H_5$), 3.72 (t, *J* = 5.47 Hz, 4H, $H_6$), 3.70 – 3.67 (m, 8H, $H_{7,8}$), 3.66 (s, 4H, $H_9$).

**$^{13}$C NMR** (151 MHz, $CDCl_3$) δ 149.03, 121.50, 114.59, 71.15, 70.94, 70.86, 70.77, 70.75, 69.89, 69.31.

**HRMS** (ESI$^+$) Calculated for $C_{20}H_{32}O_8Na$ $[M+Na]^+$ 423.1989 found 423.1977.

## 5 Synthesis of [4]rotaxane **13**

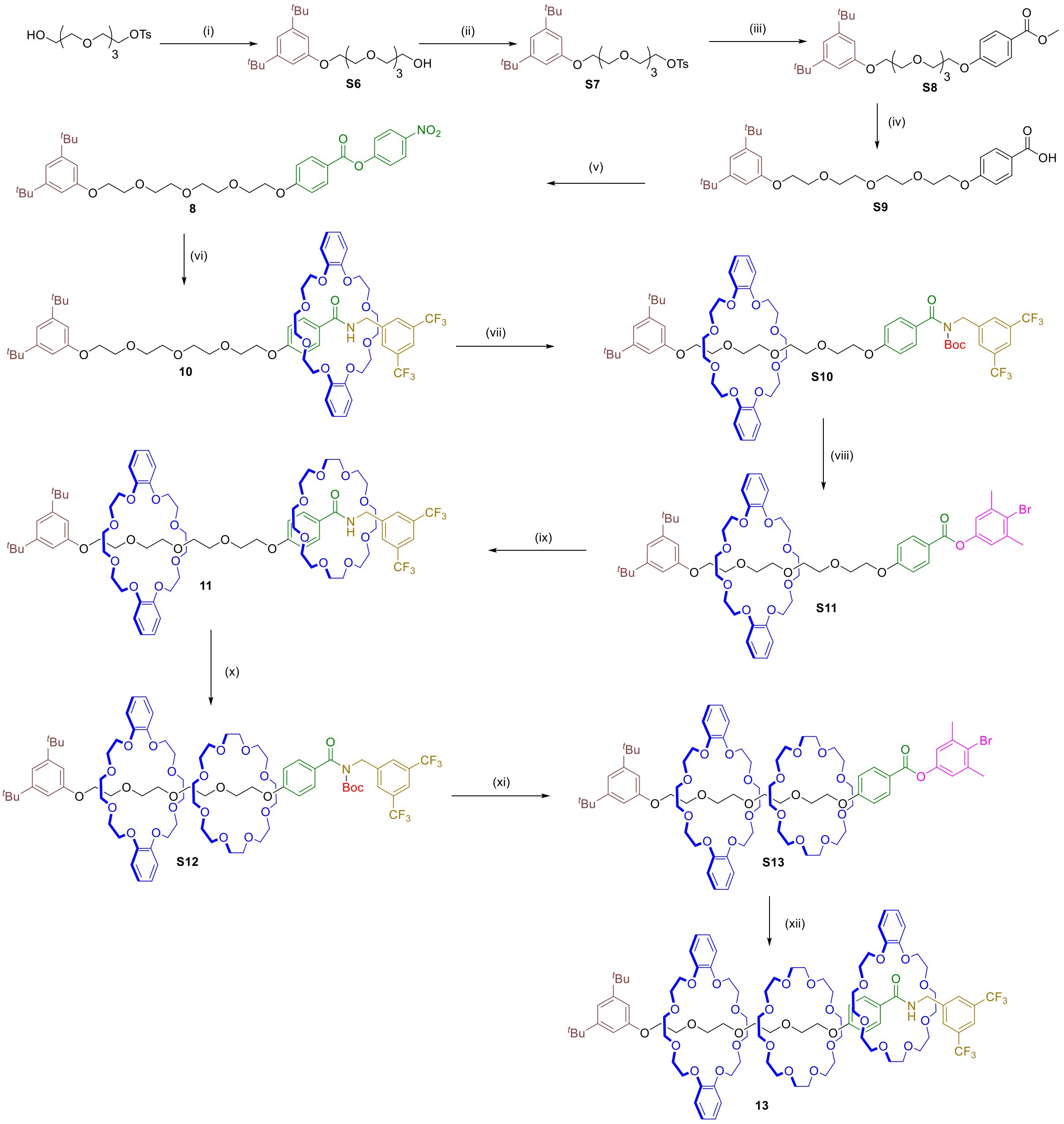


**Scheme S2**. Reagents and conditions (i) 3,5-di-*tert*-butyl-phenol, $K_2CO_3$, DMF, 80 °C, 16 h, 92%. (ii) TsCl, $Et_3N$, $CH_2Cl_2$, rt, 16 h, 90%. (iii) methyl paraben, $K_2CO_3$, DMF, 80 °C, 16 h, 83%. (iv) NaOH (5 % in $H_2O$), MeOH, 60 °C, 16 h, 98%. (v) 4-nitro-phenol, EDCI, DMAP, $CH_2Cl_2$, rt, 16 h, 85%. (vi) 3,5-bis-trifluoromethylbenzylamine, DB-24-C-8 **9**, toluene, rt, 16 h, 61%. (vii) $Boc_2O$, DMAP, THF, 90 °C, 10 h, MW, 81%. (viii) 4-bromo-3,5-dimethyl phenol, $K_3PO_4$, THF, 70 °C, 8 h, MW, 90%. (ix) 3,5-bis-trifluoromethylbenzylamine, 24-C-8 **2**, toluene, rt, 7 days, 54%. (x) $Boc_2O$, DMAP, THF, 80 °C, 4 h, MW, 75%. (xi) 4-bromo-3,5-dimethyl phenol, $K_3PO_4$, THF, 60 °C, 16 h, MW, 54%. (xii) 3,5-bis-trifluoromethylbenzylamine, B-24-C-8 **12**, toluene, rt, 21 days, 20%.

## 5.1 Synthesis of **S6**

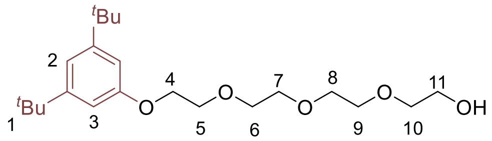


Tetraethylene glycol *p*-toluenesulfonate (2.80 g, 5.1 mmol, 1.0 eq.) and 3,5-di-*tert*-butyl-phenol (1.78 g, 5.1 mmol, 1.0 eq.) were dissolved in DMF (10 ml). To this solution was added $K_2CO_3$ (0.71 g, 5.1 mmol, 1.0 eq.). The reaction was stirred at 80 °C for 16 hours after which the reaction mixture was filtered and washed with EtOAc. The solvent was removed under reduced pressure. The residue was purified by flash column chromatography ($SiO_2$, Hexane/EtOAc 3:1) to afford **S6** as a colorless oil (1.80 g, 4.7 mmol, 92%).

**$^1$H NMR** (600 MHz, $CDCl_3$) δ 7.02 (br, 1H, $H_2$), 6.78 (d, *J* = 1.28 Hz, 2H, $H_3$), 4.15 (t, *J* = 4.92 Hz, 2H, $H_4$), 3.87 (t, *J* = 4.39 Hz , 2H, $H_5$), 3.78 – 3.75 (m, 2H, $H_{6/7}$), 3.75 – 3.71 (m, 4H, $H_{6/7,11}$), 3.69 (s, 4H, $H_{8,9}$), 3.62 (t, *J* = 4.52 Hz , 2H, $H_{10}$), 1.31 (s, 18H, $H_1$).

**$^{13}$C NMR** (151 MHz, $CDCl_3$) δ 158.4, 152.3, 115.2, 109.1, 72.6, 70.9, 70.83, 70.76, 70.5, 70.1, 67.3, 61.9, 35.1, 31.6.

**HRMS** (ESI$^+$) Calculated for $C_{22}H_{38}O_5Na$ $[M+Na]^+$ 405.2611 found 405.2617.

## 5.2 Synthesis of **S7**

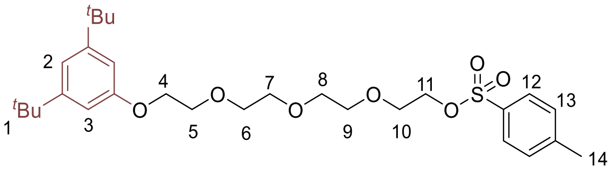


To a stirring solution of **S6** (0.63 g, 1.7 mmol, 1.0 eq.) in $CH_2Cl_2$ (2.0 ml) were added $Et_3N$ (0.30 ml) and *p*-toluenesulfonyl chloride (0.35 g, 1.8 mmol, 1.1 eq.), The reaction mixture was stirred at room temperature for 16 hours after which the solution was concentrated. The residue was purified by flash chromatography (Hexane/EtOAc 1:1) to afford **S7** as a colorless oil (0.79 g, 1.5 mmol, 90%).

**$^1$H NMR** (600 MHz, $CDCl_3$) δ 7.80 (d, *J* = 8.29 Hz, 2H, $H_{12}$), 7.34 (d, *J* = 8.37 Hz, 2H, $H_{13}$), 7.02 (br, 1H, $H_2$), 6.77 (d, *J* = 1.47 Hz, 2H, $H_3$), 4.17 – 4.13 (m, 4H, $H_{4,11}$), 3.86 (t, *J* = 4.64 Hz, 2H, $H_5$), 3.74 (t, *J* = 4.34 Hz, 2H, $H_{7/8}$) , 3.67 (t, *J* = 4.64 Hz, 2H, $H_{7/8}$), 3.69 (t, *J* = 4.04 Hz, 2H, $H_{10}$), 3.63 – 3.59 (m, 4H, $H_{6,9}$), 2.44 (s, 3H, $H_{14}$), 1.31 (s, 18H, $H_1$).

**$^{13}$C NMR** (151 MHz, $CDCl_3$) δ 158.4, 152.3, 144.9, 133.1, 130.0, 128.1, 115.2, 109.1, 70.91, 70.90, 70.8, 70.7, 70.0, 69.4, 68.8, 67.3, 35.1, 31.6, 21.8.

**HRMS** (ESI$^+$) Calculated for $C_{29}H_{45}O_7S$ $[M+H]^+$ 537.2881 found 537.2884.

## 5.3 Synthesis of **S8**

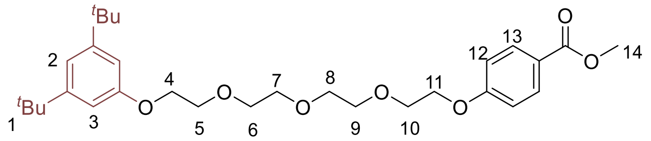

**S7** (0.39 g, 0.75 mmol, 1.0 eq.) and methyl paraben (0.14 g, 0.90 mmol, 1.2 eq.) were dissolved in DMF (10 ml). To this solution was added $K_2CO_3$ (0.31 g, 2.3 mmol, 3.0 eq.). The reaction mixture was stirred at 80 °C for 16 hours after which the mixture was filtered and extracted with EtOAc. The solvent was removed under reduced pressure and the residue was purified by flash column chromatography ($SiO_2$, Hexane/EtOAc 2:1) to afford **S8** as a colorless oil (0.32 g, 0.62 mmol, 83%).

**$^1$H NMR** (600 MHz, $CDCl_3$) δ 7.97 (d, *J* = 8.86 Hz, 2H, $H_{13}$), 7.02 (s, 1H, $H_2$), 6.92 (d, *J* = 8.90 Hz, 2H, $H_{12}$), 6.78 (d, *J* = 1.38 Hz, 2H, $H_3$), 4.17 (t, *J* = 4.62 Hz, 2H, $H_{11}$), 4.14 (t, *J* = 5.28 Hz, 2H, $H_4$), 3.88 – 3.85 (m, 4H, $H_{5,10}$), 3.88 (s, 3H, $H_{14}$), 3.75 – 3.73 (m, 4H, $H_{6,9}$), 3.71 – 3.69 (m, 4H, $H_{7,8}$), 1.30 (s, 18H, $H_1$).

**$^{13}$C NMR** (151 MHz, $CDCl_3$) δ 167.0, 162.7, 158.4, 152.3, 131.7, 122.83, 115.2, 114.3, 109.1, 71.0, 70.9, 70.84, 70.82, 70.0, 69.7, 67.7, 67.3, 52.0, 35.1, 31.6.

**HRMS** (ESI$^+$) Calculated for $C_{30}H_{44}O_7$ $[M]^+$ 516.3082 found 516.3080.

## 5.4 Synthesis of **S9**

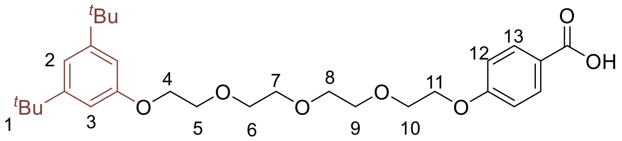


To a solution of compound **S8** (0.32 g, 0.62 mmol, 1.0 eq.) in MeOH (4.3 mL) was added NaOH aqueous solution (5% w/v, 3.4 mL). The reaction mixture was stirred at 60 °C overnight. The solvent was removed under reduced pressure and the residue was dissolved with water. The pH of the solution was adjusted to pH = 1 using 1 M HCl and extracted with $CH_2Cl_2$ (3 x 50 ml) three times. The organic layers were combined and washed with brine and dried over $Na_2SO_4$. After filtration, the solvent was removed under reduced pressure to afford **S9** as a colorless solid (0.31 g, 0.62 mmol, 98%).

**$^1$H NMR** (600 MHz, $CDCl_3$) δ 8.05 (d, *J* = 8.79 Hz, 2H, $H_{13}$), 7.04 (t, *J* = 1.75 Hz, 1H, $H_2$), 6.96 (d, *J* = 8.76 Hz, 2H, $H_{12}$), 6.79 (d, *J* = 1.74 Hz, 2H, $H_3$), 4.21 (t, *J* = 4.74 Hz, 2H, $H_{11}$), 4.16 (t, *J* = 5.00 Hz, 2H, $H_4$), 3.91 (t, *J* = 4.75 Hz, 2H, $H_{10}$), 3.88 (t, *J* = 5.08 Hz, 2H, $H_5$), 3.77 – 3.74 (m, 4H, $H_{6,9}$), 3.74 – 3.70 (m, 4H, $H_{7,8}$), 1.32 (s, 18H, $H_1$).

**$^{13}$C NMR** (151 MHz, $CDCl_3$) δ 163.4, 158.5, 152.3, 132.4, 115.3, 114.5, 109.1, 71.0, 70.94, 70.86, 70.8, 70.0, 69.7, 67.8, 67.3, 35.1, 31.6.

**HRMS** ($ESI^-$) Calculated for $C_{29}H_{41}O_7$ $[M–H]^-$ 501.2858 found 501.2837.

## 5.5 Synthesis of **8**

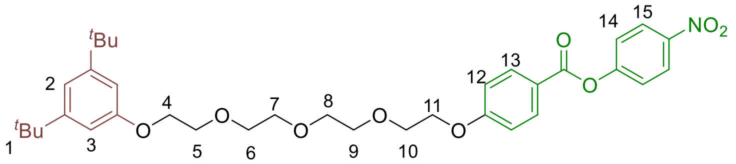


To a solution of **S9** (0.31 g, 0.62 mmol, 1.0 eq.) in $CH_2Cl_2$ (6 mL) were added 4-nitro-phenol (0.10 g, 0.73 mmol, 1.2 eq.), DMAP (0.080 g, 0.62 mmol, 1.0 eq.) and EDCI (0.13 g, 0.68 mmol, 1.1 eq.). The reaction mixture was stirred for 16 hours at room temperature. The solvent was removed under reduced pressure and the residue was purified by flash column chromatography ($SiO_2$, Hexane/EtOAc 2:1) to afford **8** as a colorless oil (0.33 g, 0.53 mmol, 85%).

**$^1$H NMR** (600 MHz, $CDCl_3$) δ 8.32 (d, $J$ = 8.93 Hz, 2H, $H_{15}$), 8.13 (d, $J$ = 9.04 Hz, 2H, $H_{13}$), 7.40 (d, $J$ = 8.85 Hz, 2H, $H_{14}$), 7.02 (s, 1H, $H_2$), 7.01 (d, $J$ = 9.00 Hz, 2H, $H_{12}$), 6.78 (d, $J$ = 1.52 Hz, 2H, $H_3$), 4.22 (t, $J$ = 4.70 Hz, 2H, $H_{11}$), 4.15 (t, $J$ = 5.08 Hz, 2H, $H_4$), 3.91 (t, $J$ = 4.57 Hz, 2H, $H_{10}$), 3.87 (t, $J$ = 5.30 Hz, 2H, $H_5$), 3.77 – 3.74 (m, 4H, $H_{6,9}$), 3.73 – 3.70 (m, 4H, $H_{7,8}$), 1.31 (s, 18H, $H_1$).

**$^{13}$C NMR** (126 MHz, $CDCl_3$) 13C NMR (126 MHz, CDCl3) δ 164.0, 163.8, 158.4, 156.1, 152.3, 145.4, 132.6, 125.4, 122.8, 120.9, 115.3, 114.8, 109.1, 71.1, 70.9, 70.84, 70.82, 70.0, 69.6, 67.9, 67.3, 35.1, 31.6.

**HRMS** (ESI$^+$) Calculated for $C_{35}H_{45}O_9NNa$ $[M+Na]^+$ 646.2987 found 646.2968.

## 5.6 Synthesis of **10**

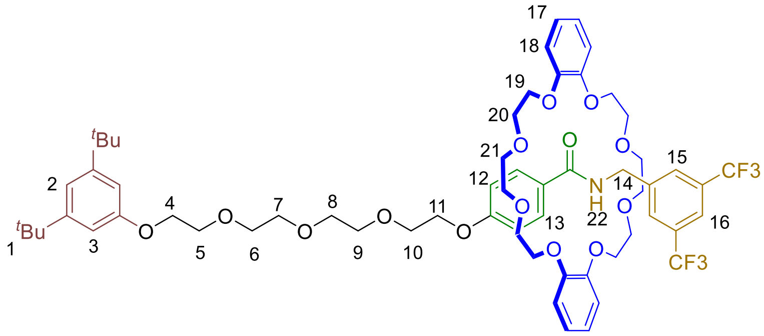


To a solution of compound **8** (0.31 g, 0.50 mmol, 1.0 eq.) in toluene (3.3 mL) were added DB-24-C-8 **9** (0.34 g, 0.75 mmol, 1.5 eq.) and 3,5-bis-trifluoromethylbenzylamine (0.18 g, 0.75 mmol, 1.5 eq.). The reaction mixture was stirred at room temperature for 16 hours. The solvent was removed under reduced pressure and the residue was purified by flash column chromatography ($SiO_2$, $CH_2Cl_2$/MeOH 25:1) to afford **10** as a colorless oil (0.36 g, 0.31 mmol, 61%).

**$^1$H NMR** (600 MHz, $CDCl_3$) δ 8.27 (s, 2H, $H_{15}$), 8.10 (d, *J* = 8.72 Hz , 2H, $H_{13}$), 8.04 (s, 1H, $H_{16}$), 7.02 (s, 1H, $H_2$), 6.84 (br, 1H, $H_{22}$), 6.82 (d, *J* = 8.52 Hz, 2H, $H_{12}$), 6.77 (d, *J* = 1.14 Hz, 2H, $H_3$), 6.64 – 6.61 (m, 4H, $H_{17}$), 6.47 – 6.44 (m, 4H, $H_{18}$), 5.01 (d, *J* = 3.62 Hz, 2H, $H_{14}$), 4.07 – 4.02 (m, 4H, $H_{4,11}$), 3.93 (t, *J* = 7.69 Hz , 4H, $H_{19}$), 3.81 – 3.75 (m, 8H, $H_{5,10,19}$) 3.72 (dd, *J* = 10.51, 4.13 Hz, 4H, $H_{6/7/8/9}$), 3.67 – 3.63 (m, 4H, $H_{20}$), 3.63 – 3.60 (m, 4H, $H_{20}$), 3.57 – 3.48 (m, 8H, $H_{21}$), 3.43 – 3.37 (m, 4H, $H_{6/7/8/9}$), 1.30 (s, 18H, $H_1$).

**$^{13}$C NMR** (126 MHz, $CDCl_3$) δ 165.8, 160.6, 158.4, 152.3, 147.2, 141.2, 132.4, 129.8, 128.4 (q, *J* = 32.1 Hz), 128.3, 124.0 (q, *J* = 272.6 Hz), 120.2, 118.8 (m), 115.2, 113.8, 111.1, 109.1, 71.0, 70.9, 70.81, 70.79, 70.4, 70.0, 69.8, 69.6, 67.8, 67.5, 67.3, 44.3, 35.1, 31.6.

**HRMS** (ESI$^+$) Calculated for $C_{62}H_{79}O_{14}NF_6Na$ $[M + Na]^+$ 1198.5297 found 1198.5279.

## 5.7 Synthesis of **S10**

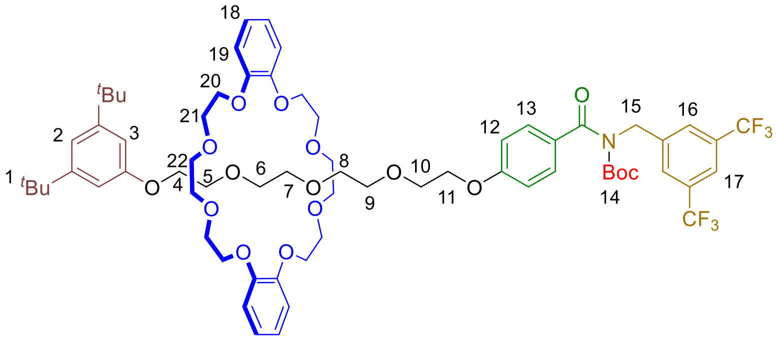


To a solution of compound **10** (0.59 g, 0.50 mmol, 1.0 eq.) in THF (5 ml) in a microwave vial were added DMAP (0.070 g, 0.60 mmol, 1.2 eq.) and $Boc_2O$ (1.50 ml, 3.00 mmol, 2M THF solution, 6.0 eq.). The reaction mixture was sealed and stirred under microwave irradiation at 90 °C for 10 hours. The mixture was cooled to room temperature and the solvent was removed under reduced pressure. Purification of the residue by flash column chromatography ($SiO_2$, $CH_2Cl_2$/MeOH 30:1) yielded **S10** as a colorless oil (0.52 g, 0.41 mmol, 81%).

**$^1$H NMR** (600 MHz, $CDCl_3$) δ 7.87 (s, 2H, $H_{16}$), 7.78 (s, 1H, $H_{17}$), 7.39 (d, $J$ = 8.93 Hz, 2H, $H_{13}$), 7.26 (d, $J$ = 8.19 Hz, 2H, $H_{12}$), 7.00 (s, 1H, $H_2$), 6.90 – 6.86 (m, 4H, $H_{18}$), 6.86 – 6.82 (m, 4H, $H_{19}$), 6.77 (s, 2H, $H_3$), 4.98 (s, 2H, $H_{15}$), 4.56 (t, $J$ = 5.39 Hz, 2H, $H_{11}$), 4.23 – 4.18 (m, 4H, $H_{20}$), 4.16 – 4.09 (m, 6H, $H_{4, 20}$), 3.96 (t, $J$ = 5.83 Hz, 2H, $H_{10}$), 3.85 – 3.80 (m, 10H, $H_{5, 21}$), 3.65 (t, $J$ = 4.63 Hz, 2H, $H_{8/9}$), 3.61 (t, $J$ = 4.76 Hz, 2H, $H_6$), 3.58 – 3.55 (m, 6H, $H_{8/9, 22}$), 3.51 (t, $J$ = 5.26 Hz, 2H, $H_7$), 3.40 – 3.33 (m, 4H, $H_{22}$), 1.30 (s, 18H, $H_1$), 1.15 (s, 9H, $H_{14}$).

**$^{13}$C NMR** (126 MHz, $CDCl_3$) δ 172.9, 163.2, 158.5, 153.6, 152.2, 148.7, 141.0, 131.8 (q, $J$ = 33.2 Hz), 130.0, 128.6 (q, $J$ = 3.9 Hz), 127.1, 123.4 (q, $J$ = 272.8 Hz), 121.5 (m), 121.0, 115.6, 115.1, 112.7, 109.1, 83.5, 70.7, 70.51, 70.45, 70.00, 69.99, 69.9, 69.7, 69.4, 68.3, 67.2, 67.1, 48.7, 35.1, 31.6, 27.6.

**HRMS** (ESI$^+$) Calculated for $C_{67}H_{87}O_{16}NF_6Na$ $[M+Na]^+$ 1298.5821 found 1298.5784.

## 5.8 Synthesis of **S11**

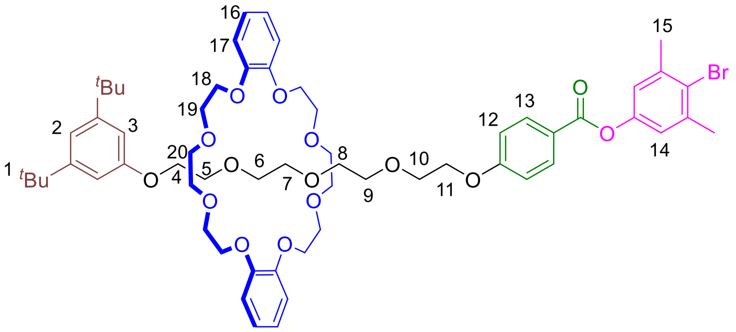


To a solution of compound **S10** (0.26 g, 0.20 mmol, 1.0 eq.) in THF (2 ml) in a microwave vial were added 4-bromo-3,5-dimethyl phenol (0.12 g, 0.60 mmol, 3.0 eq.) and $K_3PO_4$ (0.19 g, 0.90 mmol, 4.5 eq.). The reaction mixture was sealed and stirred under microwave irradiation at 70 °C for 8 hours. The mixture was cooled to room temperature and the solvent was removed under reduced pressure. Purification of the residue by flash column chromatography ($SiO_2$, $CH_2Cl_2$/MeOH 40:1) yielded **S11** as a colorless oil (0.20 g, 0.18 mmol, 90%).

**$^1$H NMR** (600 MHz, $CDCl_3$) δ 7.95 (d, $J$ = 8.28 Hz, 2H, $H_{13}$), 7.58 (d, $J$ = 8.87 Hz, 2H, $H_{12}$), 7.01 (br, 1H, $H_2$), 6.92 (s, 2H, $H_{14}$), 6.90 – 6.87 (m, 4H, $H_{16}$), 6.87 – 6.84 (m, 4H, $H_{17}$), 6.77 (d, $J$ = 1.54 Hz, 2H, $H_3$), 4.62 (t, $J$ = 5.30 Hz, 2H, $H_{11}$), 4.23 – 4.19 (m, 4H, $H_{18}$), 4.15 – 4.10 (m, 6H, $H_{4, 18}$), 3.90 (t, $J$ = 5.29 Hz, 2H, $H_{10}$), 3.83 (t, $J$ = 5.08 Hz, 2H, $H_5$), 3.81 (t, $J$ = 4.47 Hz, 8H, $H_{19}$), 3.67 (t, $J$ = 4.40 Hz, 2H, $H_{6/9}$), 3.59 (q, $J$ = 4.77 Hz, 4H, $H_{6/7/8/9}$), 3.54 – 3.50 (m, 6H, $H_{7/8, 20}$), 3.24 – 3.21 (m, 4H, $H_{20}$) , 2.42 (s, 6H, $H_{15}$), 1.30 (s, 18H, $H_1$).

**$^{13}$C NMR** (151 MHz, $CDCl_3$) δ 165.7, 164.3, 158.5, 152.3, 149.7, 148.6, 139.5, 131.5, 123.9, 121.7, 121.0, 120.3, 116.8, 115.1, 112.5, 109.1, 70.8, 70.60, 70.56, 70.1, 69.9, 69.77, 69.75, 69.7, 68.3, 67.2, 66.9, 35.1, 31.6, 24.1.

**HRMS** (ESI$^+$) Calculated for $C_{61}H_{81}O_{15}BrNa$ $[M+Na]^+$ 1155.4651 found 1155.4605.

## 5.9 Synthesis of **11**

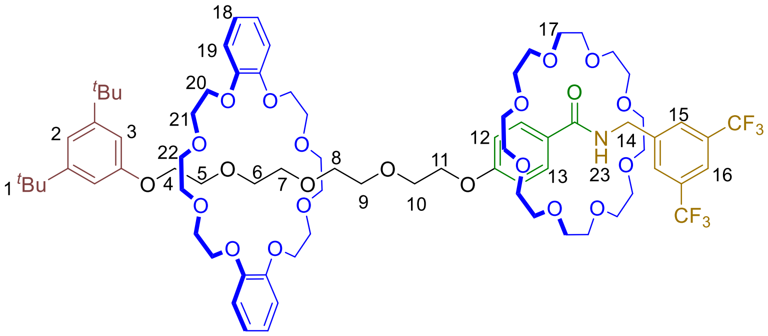


To a solution of compound **S11** (0.42 g, 0.37 mmol, 1.0 eq.) in toluene (2.5 mL) were added 24-C-8 **2** (0.26 g, 0.75 mmol, 2.0 eq.) and 3,5-bis-trifluoromethylbenzylamine (0.18 g, 0.75 mmol, 2.0 eq.). The reaction mixture was stirred at room temperature for 7 days. The solvent was removed under reduced pressure and the residue was purified by flash column chromatography ($SiO_2$, $CH_2Cl_2$/MeOH 25:1) to afford **11** as a colorless oil (0.31 g, 0.20 mmol, 54%).

**$^1$H NMR** (600 MHz, $CDCl_3$) δ 8.67 (s, 2H, $H_{15}$), 7.92 (d, *J* = 8.83 Hz, 2H, $H_{13}$), 7.76 (br, 1H, $H_{23}$), 7.68 (s, 1H, $H_{16}$), 7.53 (br, 2H, $H_{12}$), 6.99 (s, 1H, $H_2$), 6.86 – 6.83 (m, 4H, $H_{18}$), 6.82 – 6.79 (m, 4H, $H_{19}$), 6.76 (d, *J* = 1.43 Hz, 2H, $H_3$), 4.86 (d, *J* = 3.61 Hz, 2H, $H_{14}$), 4.49 (t, *J* = 5.36 Hz, 2H, $H_{11}$), 4.17 – 4.11 (m, 6H, $H_{4, 20}$), 4.08 – 4.04 (m, 4H, $H_{20}$), 3.87 – 3.77 (m, 12 H, $H_{5, 10, 21}$), 3.66 (t, *J* = 4.42 Hz, 2H, $H_{6/7/8/9}$), 3.62 – 3.58 (m, 4H, $H_{6/7/8/9}$), 3.53 (t, *J* = 5.28 Hz, 2H, $H_{6/7/8/9}$), 3.45 (q, *J* = 5.10 Hz, 4H, $H_{22}$), 3.34 – 3.30 (m, 16H, $H_{17}$), 3.14 – 3.10 (m, 20H, $H_{17, 22}$), 1.30 (s, 18H, $H_1$).

**$^{13}$C NMR** (151 MHz, $CDCl_3$) δ 167.2, 161.5, 158.5, 152.2, 148.7, 141.3, 135.7, 128.9, 128.8 (q, *J* = 29.9 Hz), 124.3 (q, *J* = 272.3 Hz), 120.8, 119.7, 116.0, 115.0, 112.2, 109.1, 70.71, 70.69, 70.6, 70.52, 70.46, 70.1, 70.0, 69.9, 69.7, 69.4, 68.2, 67.2, 66.4, 44.6, 35.1, 31.6.

**HRMS** (ESI$^+$) Calculated for $C_{78}H_{111}O_{22}NF_6Na$ $[M+Na]^+$ 1550.7394 found 1550.7333.

## 5.10 Synthesis of **S12**

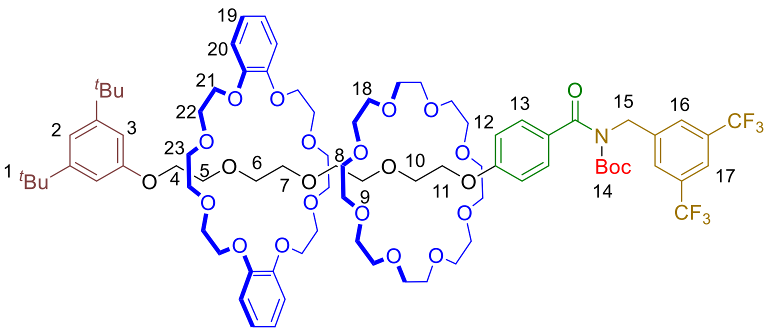

To a solution of compound **11** (0.15 g, 0.10 mmol, 1.0 eq.) in THF (1 ml) in a microwave vial were added DMAP (0.015 g, 0.12 mmol, 1.2 eq.) and $Boc_2O$ (0.3 ml, 0.60 mmol, 2M THF solution, 6.0 eq.). The reaction mixture was sealed and stirred under microwave irradiation at 80 °C for 4 hours. The mixture was cooled to room temperature and the solvent was removed under reduced pressure. Purification of the residue by flash column chromatography ($SiO_2$, $CH_2Cl_2$/MeOH 20:1) yielded **S12** as a colorless oil (0.12 g, 0.074 mmol, 75%).

**$^1$H NMR** (600 MHz, $CDCl_3$) δ 7.89 (s, 2H, $H_{16}$), 7.79 (s, 1H, $H_{17}$), 7.53 (d, *J* = 8.67 Hz, 2H, $H_{13}$), 7.23 (d, *J* = 8.97 Hz, 2H, $H_{12}$), 7.00 (s, 1H, $H_2$), 6.87 – 6.84 (m, 8H, $H_{19, 20}$), 6.83 (s, 2H, $H_3$), 5.02 (s, 2H, $H_{15}$), 4.53 (t, *J* = 5.15 Hz, 2H, $H_{11}$), 4.42 (t, *J* = 5.62 Hz, 2H, $H_4$), 4.22 – 4.17 (m, 4H, $H_{21}$), 4.16 – 4.12 (m, 4H, $H_{21}$), 4.03 (t, *J* = 5.77 Hz, 2H, $H_5$), 3.92 (t, *J* = 4.84 Hz, 2H, $H_{10}$), 3.89 (t, *J* = 5.70 Hz, 8H, $H_{22}$), 3.73 (t, *J* = 5.55 Hz, 2H, $H_{6/7/8/9}$), 3.71 – 3.66 (m, 4H, $H_{6/7/8/9}$) 3.65 – 3.59 (m, 6H, $H_{23, 6/7/8/9}$), 3.58 – 3.51 (m, 20H, $H_{18, 23}$), 3.47 – 3.42 (m, 16H, $H_{18}$), 1.20 (s, 9H, $H_{14}$), 1.19 (s, 18H, $H_1$).

**$^{13}$C NMR** (151 MHz, $CDCl_3$) δ 173.0, 163.4, 159.2, 153.7, 151.5, 149.0, 141.0, 131.8 (q, *J* = 33.5 Hz), 130.1, 128.5 (q, *J* = 4.0 Hz), 127.3, 123.4 (q, *J* = 273.1 Hz), 121.5, 121.1, 115.5, 113.8, 113.4, 109.8, 83.5, 70.4, 70.3, 70.2, 70.0, 69.74, 69.68, 69.60, 69.57, 68.3, 68.2, 67.5, 66.8, 48.6, 35.0, 31.5, 27.6.

**HRMS** ($ESI^+$) Calculated for $C_{83}H_{119}O_{24}NF_6Na$ $[M+Na]^+$ 1650.7918 found 1650.7865.

## 5.11 Synthesis of **S13**

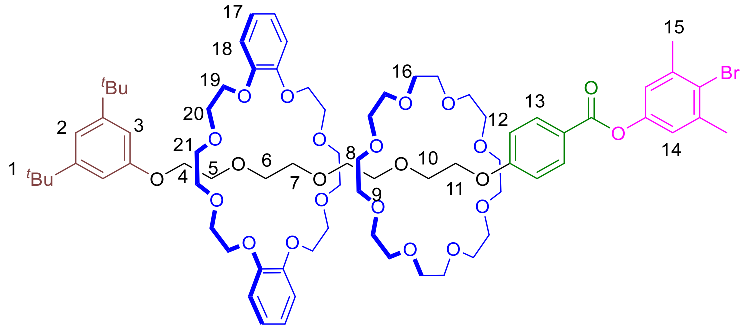


To a solution of compound **S12** (0.12 g, 0.074 mmol, 1.0 eq.) in THF (2 ml) in a microwave vial were added 4-bromo-3,5-dimethyl phenol (0.045 g, 0.22 mmol, 3.0 eq.) and $K_3PO_4$ (0.071 g, 0.33 mmol, 4.5 eq.). The reaction mixture was sealed and stirred under microwave irradiation at 60 °C for 16 hours. The mixture was cooled to room temperature and the solvent was removed under reduced pressure. Purification of the residue by flash column chromatography ($SiO_2$, $CH_2Cl_2$/MeOH 20:1) yielded **S13** as a colorless oil (0.060 g, 0.040 mmol, 54%).

**$^1$H NMR** (600 MHz, $CDCl_3$) δ 8.09 (d, *J* = 8.83 Hz, 2H, $H_{13}$), 7.37 (d, *J* = 8.35 Hz, 2H, $H_{12}$), 6.97 (s, 2H, $H_{14}$), 6.88 – 6.84 (m, 9H, $H_{2,\ 17,\ 18}$), 6.83 (d, *J* = 1.46 Hz, 2H, $H_3$), 4.58 (t, *J* = 5.02 Hz, 2H, $H_{11}$), 4.41 (t, *J* = 5.77 Hz, 2H, $H_4$), 4.01 (t, *J* = 5.40 Hz, 2H, $H_5$), 4.22 – 4.19 (m, 4H, $H_{19}$), 4.17 – 4.11 (m, 4H, $H_{19}$), 3.92 (*t, J* = 4.83 Hz, 2H, $H_{10}$), 3.89 (t, *J* = 5.14 Hz, 8H, $H_{20}$), 3.73 (t, *J* = 5.88 Hz, 2H, $H_{6/7/8/9}$), 3.71 – 3.65 (m, 4H, $H_{21}$), 3.65 – 3.60 (m, 8H, $H_{6/7/8/9,\ 21}$), 3.60 – 3.52 (m, 18H, $H_{6/7/8/9,\ 16}$), 3.47 – 3.42 (m, 16H, $H_{16}$), 2.43 (s, 6H, $H_{15}$), 1.20 (s, 18H, $H_1$).

**$^{13}$C NMR** (151 MHz, $CDCl_3$) δ 165.6, 164.5, 159.2, 151.6, 149.8, 149.0, 139.5, 131.8, 124.0, 121.7, 121.1, 120.2, 116.2, 113.8, 113.3, 109.8, 70.3 (2C), 70.1 (2C), 70.0, 69.8, 69.7, 69.61, 69.58, 68.3, 67.3, 66.8, 35.0, 31.5, 24.1.

**HRMS** (ESI$^+$) Calculated for $C_{77}H_{113}BrO_{23}Na$ $[M+Na]^+$ 1507.6748 found 1507.6734.

## 5.12 Synthesis of **13**

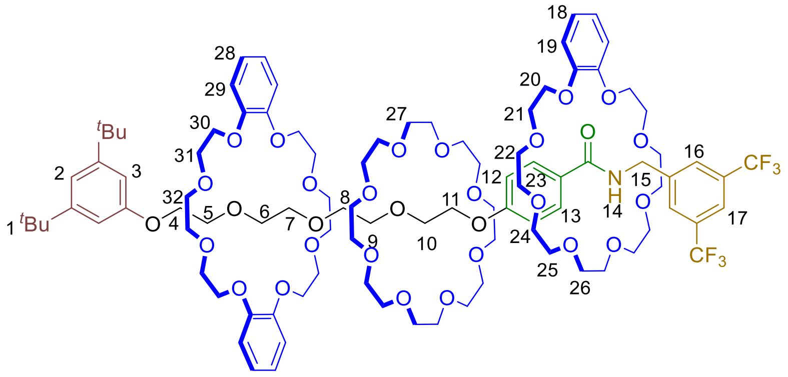


To a solution of **S13** (0.030 g, 0.020 mmol, 1.0 eq.) in toluene (0.3 mL) were added B-24-C-8 **12** (0.017 g, 0.040 mmol, 2.0 eq.) and 3,5-bis-trifluoromethylbenzylamine (0.0097 mg, 0.040 mmol, 2.0 eq.). The reaction mixture was stirred at room temperature for 21 days. The solvent was removed under reduced pressure. Purification of the residue by flash column chromatography ($SiO_2$, $CH_2Cl_2$/MeOH 15:1) yielded **13** as a colorless oil (0.008 g, 4.0 µmol, 20%).

**$^1$H NMR** (600 MHz, $CDCl_3$) δ 8.57 (s, 2H, $H_{16}$), 7.92 (d, $J$ = 7.82 Hz, 2H, $H_{13}$), 7.86 (br, 1H, $H_{14}$), 7.42 (s, 1H, $H_{17}$), 7.09 (d, $J$ = 7.54 Hz, 2H, $H_{12}$), 6.88 – 6.82 (m, 11H, $H_{2, 3, 28, 29}$), 6.73 (q, $J$ = 3.82 Hz, 2H, $H_{18}$), 6.58 (q, $J$ = 3.45 Hz, 2H, $H_{19}$), 5.01 (d, $J$ = 3.03 Hz, 2H, $H_{15}$), 4.42 (d, $J$ = 5.14 Hz, 4H, $H_{4,11}$), 4.21 – 4.17 (m, 4H, $H_{30}$), 4.16 – 4.12 (m, 4H, $H_{30}$), 4.01 (t, $J$ = 5.08 Hz, 2H, $H_5$), 3.98 (t, $J$ = 6.07 Hz, 4H, $H_{20}$), 3.90 br, 2H, $H_{10}$), 3.88 (t, $J$ = 5.61 Hz, 8H, $H_{31}$), 3.78 (t, $J$ = 6.64 Hz, 4H, $H_{21}$), 3.72 (t, $J$ = 5.08 Hz, 2H, $H_{6/9}$), 3.65 – 3.58 (m, 10H, $H_{6/9, 32}$), 3.55 – 3.52 (m, 8H, $H_{24,25}$), 3.52 – 3.48 (m, 18H, $H_{7/8,27}$), 3.45 – 3.40 (m, 16H, $H_{27}$), 3.39 – 3.30 (m, 8H, $H_{22/26}$), 3.20 (t, $J$ = 6.75 Hz, 4H, $H_{23}$), 3.02 (t, $J$ = 6.75 Hz, 2H,$H_{26}$), 1.18 (s, 18H, $H_1$).

**$^{13}$C NMR** (151 MHz, $CDCl_3$) δ 167.7, 161.9, 159.1, 151.4, 148.8, 147.6, 133.7, 129.5, 129.0 (q, $J$ = 30.95 Hz), 125.3, 123.4 (q, $J$ = 272.9 Hz), 120.9, 120.4, 119.7, 114.9, 113.6, 113.2, 111.5, 109.7, 71.1, 70.8 (6C), 70.63, 70.5, 70.39, 70.36, 70.2, 70.1, 70.0, 69.8, 69.7, 68.4, 68.3, 67.5, 67.0, 34.9, 31.4.

**HRMS** (ESI$^+$) Calculated for $C_{98}H_{143}O_{30}NF_6Na$ $[M+Na]^+$ 1950.9491 found 1950.9431.

# 6 Synthesis of [5]rotaxane **16**

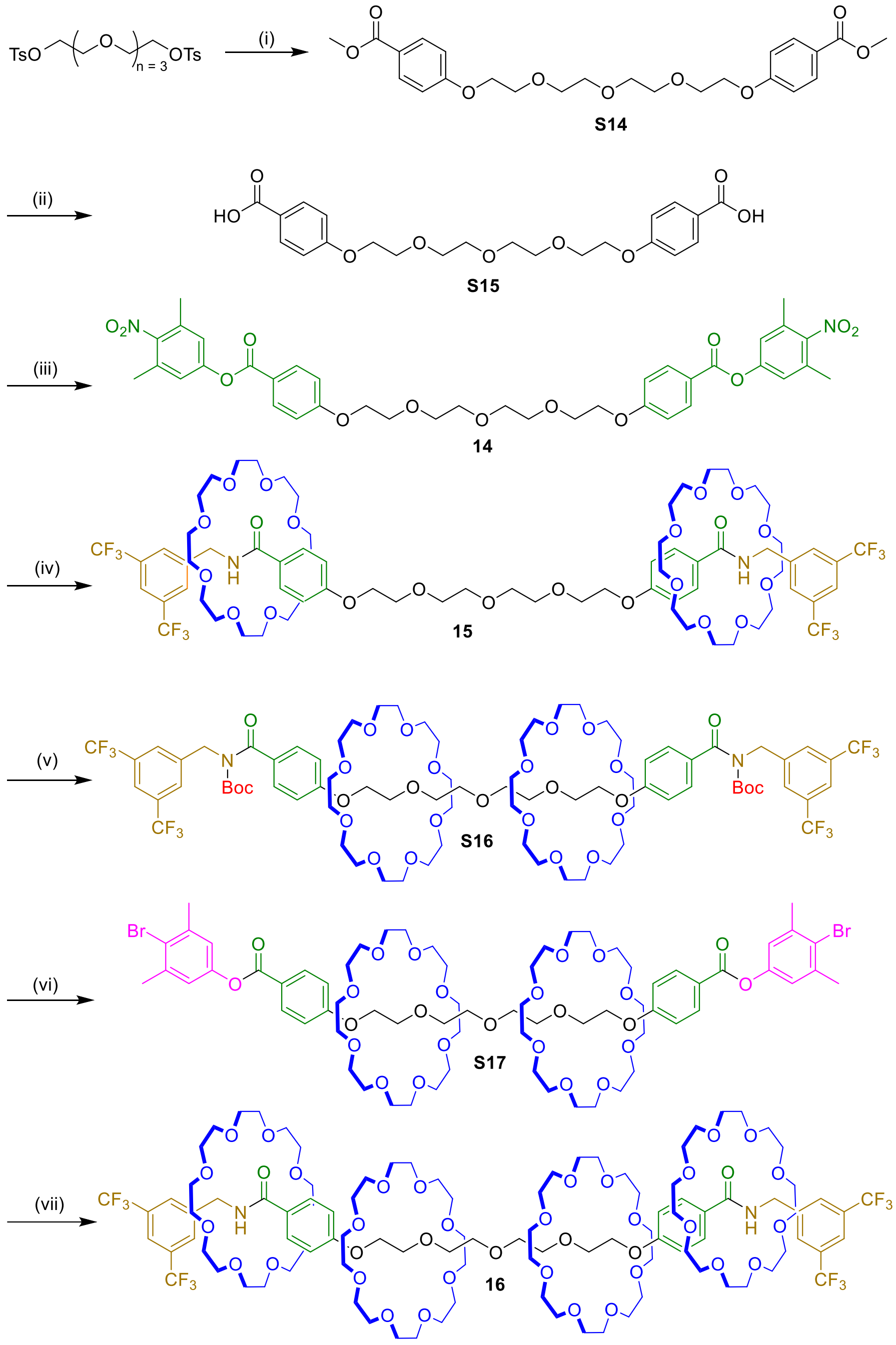


**Scheme S3**. Reagents and conditions (i) methyl paraben, $K_2CO_3$, DMF, 80 °C, 16 h, 95%. (ii) NaOH (5 % in $H_2O$), MeOH, 60 °C, 16 h, 86% (iii) 3,5-dimethyl-nitrophenol, EDCI, DMAP, $CH_2Cl_2$, rt, 16 h, 49%. (iv) 3,5-bis-trifluoromethylbenzylamine, 24-C-8 **2**, toluene, 50 °C, 16 h, 60%. (v) $Boc_2O$, DMAP, THF, 80 °C, 10h, MW, 80%. (vi) 4-bromo-3,5-dimethyl phenol, $K_3PO_4$, THF, 60 °C, 16 h, MW, 53%. (vii) 3,5-bis-trifluoromethylbenzylamine, 24-C-8 **2**, toluene, rt, 21 days, 35%.

## 6.1 Synthesis of **S14**

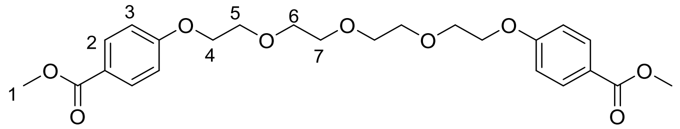


Tetraethylene glycol di(p-toluenesulfonate) (0.30 g, 0.60 mmol, 1.0 eq.) and methyl paraben (0.18 g, 1.2 mmol, 2.0 eq.) were dissolved in DMF (4 ml). To this solution was added $K_2CO_3$ (0.164 g, 1.2 mmol, 2.0 eq.). The reaction was stirred at 80 °C for 16 hours after which the solution was concentrated under reduced pressure. The residue was purified by flash column chromatography ($SiO_2$, EtOAc/Hexane 3:1) to afford **S14** as a colorless solid (0.28 g, 0.58 mmol, 95%).

**$^1$H NMR** (600 MHz, $CDCl_3$) δ 7.99 (d, $J$ = 9.30 Hz, 4H, $H_2$), 6.94 (d, $J$ = 9.03 Hz, 4H, $H_3$), 4.19 (t, $J$ = 4.54 Hz, 4H, $H_4$), 3.90 (s, 6H, $H_1$), 3.89 (t, $J$ = 5.14 Hz, 4H, $H_5$), 3.76 – 3.74 (m, 4H, $H_6$), 3.72 – 3.70 (m, 4H, $H_7$).

**$^{13}$C NMR** (151 MHz, $CDCl_3$) δ 167.0, 162.7, 131.7, 122.9, 114.3, 71.0, 70.8, 69.7, 67.7, 52.0.

**HRMS** (ESI$^+$) Calculated for $C_{24}H_{30}O_9Na$ $[M+Na]^+$ 485.1782 found 485.1781.

## 6.2 Synthesis of **S15**

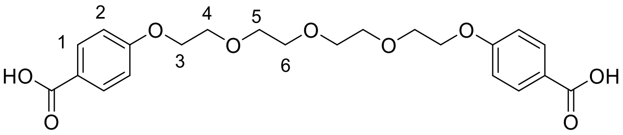


To a solution of **S14** (0.28 g, 0.58 mmol, 1.0 eq.) in MeOH (4.3 mL) was added NaOH as an aqueous solution (5% w/v, 3.4 mL). The reaction mixture was stirred at 60 °C for 16 hours. The solvent was removed under reduced pressure and the residue was purified by flash column chromatography ($SiO_2$, $CH_2Cl_2$/MeOH 25:1) to afford **S15** as a colorless solid (0.22 g, 0.50 mmol, 86%).

**$^1$H NMR** (600 MHz, $(CD_3)_2SO$) δ 12.62 (br s, 2H, –COOH), 7.88 (d, *J* = 9.17 Hz, 4H, $H_1$), 7.02 (d, *J* = 9.78 Hz, 4H, $H_2$), 4.15 (t, *J* = 4.78 Hz, 4H, $H_3$), 3.76 (t, *J* = 4.87 Hz, 4H, $H_4$), 3.59 – 3.57 (m, 4H, $H_5$), 3.56 – 3.53 (m, 4H, $H_6$).

**$^{13}$C NMR** (151 MHz $(CD_3)_2SO$) δ 167.0, 162.1, 131.4, 123.0, 114.3, 69.9, 69.8, 68.8, 67.4.

**HRMS** (ESI$^+$) Calculated for $C_{22}H_{26}O_9Na$ $[M+Na]^+$ 457.1469 found 457.1474.

## 6.3 Synthesis of **14**

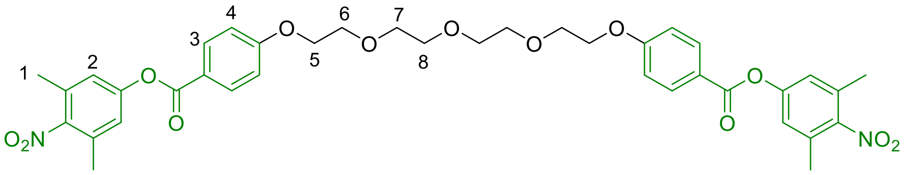


To a solution of **S15** (0.22 g, 0.50 mmol, 1.0 eq.) in $CH_2Cl_2$ (5 mL) were added 3,5-dimethyl-nitrophenol (0.17 g, 1.0 mmol, 2.0 eq.), DMAP (0.12 g, 1.0 mmol, 2.0 eq.) and EDCI (0.21 g, 1.1 mmol, 2.2 eq.). The reaction mixture was stirred for 16 hours at room temperature. The solvent was removed under reduced pressure and the residue was purified by flash column chromatography ($SiO_2$, Hexane/EtOAc 2:1) to afford **14** as a colorless solid (0.18 g, 0.25 mmol, 49%).

**$^1$H NMR** (600 MHz, $CDCl_3$) δ 8.13 (d, *J* = 7.88 Hz, 4H, $H_3$), 7.01 (d, *J* = 7.87 Hz, 4H, $H_4$), 7.00 (s, 4H, $H_2$), 4.24 (t, *J* = 4.83 Hz, 4H, $H_5$), 3.93 (t, *J* = 4.81 Hz, 4H, $H_6$), 3.79 – 3.76 (m, 4H, $H_7$), 3.75 – 3.72 (m, 4H, $H_8$), 2.36 (s, 12H, $H_1$).

**$^{13}$C NMR** (151 MHz, $CDCl_3$) δ 164.5, 163.6, 151.4, 149.4, 132.5, 131.9, 122.3, 121.4, 114.7, 71.1, 70.9, 69.7, 67.9, 17.9.

**HRMS** (ESI$^+$) Calculated for $C_{38}H_{40}O_{13}N_2Na$ $[M+Na]^+$ 755.2423 found 755.2411.

## 6.4 Synthesis of **15**

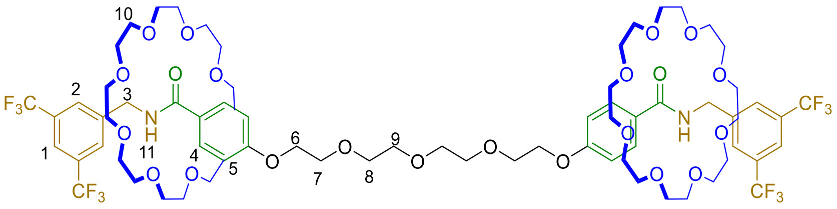


To a solution of **14** (0.18 g, 0.24 mmol, 1.0 eq.) in toluene (5 mL) were added 24-C-8 **2** (0.33 g, 0.94 mmol, 4.0 eq.) and 3,5-bis-trifluoromethylbenzylamine (0.11 g, 0.47 mmol, 2.0 eq.). The reaction mixture was stirred at 50 °C for 16 hours. The solvent was removed under reduced pressure and the residue was purified by flash column chromatography ($SiO_2$, $CH_2Cl_2$/MeOH 25:1) to afford **15** as a colorless oil (0.23 g, 0.14 mmol, 60%).

**$^1$H NMR** (600 MHz, $CDCl_3$) δ 8.73 (s, 4H, $H_2$), 8.01 (d, *J* = 8.05 Hz, 4H, $H_4$), 7.81 (br, 2H, $H_{11}$), 7.74 (s, 2H, $H_1$), 6.86 (d, *J* = 9.06 Hz, 4H, $H_5$), 4.93 (d, *J* = 3.44 Hz, 4H, $H_3$), 4.15 (t, *J* = 4.42 Hz, 4H, $H_6$), 3.87 (t, *J* = 5.41 Hz, 4H, $H_7$), 3.75 – 3.73 (m, 4H, $H_8$), 3.71 – 3.69 (m, 4H, $H_9$), 3.43 – 3.39 (m, 32H, $H_{10}$), 3.20 – 3.15 (m, 32H, $H_{10}$).

**$^{13}$C NMR** (151 MHz, $CDCl_3$) δ 166.8, 160.7, 141.3, 135.6, 130.1, 129.0 (q, *J* = 32.5 Hz), 128.2, 124.3 (q, *J* = 272.4 Hz), 119.8 (m), 113.6, 71.0, 70.8, 70.6, 69.8, 67.5, 44.7.

**HRMS** (ESI$^+$) Calculated for $C_{72}H_{100}O_{23}N_2F_{12}Na$ $[M+Na]^+$ 1611.6417 found 1611.6372.

## 6.5 Synthesis of **S16**

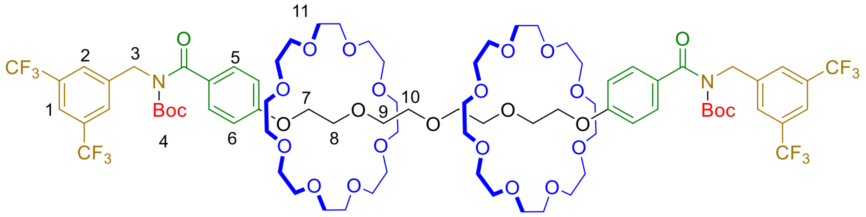


To a THF (4 mL) solution of **15** (0.23 g, 0.14 mmol, 1.0 eq.) in a microwave vial were added DMAP (0.0067 g, 0.056 mmol, 0.4 eq.) and $Boc_2O$ (0.36 g, 1.7 mmol, 12.0 eq.). The reaction mixture was sealed and stirred under microwave irradiation at 80 °C for 10 hours. The mixture was cooled to room temperature and the solvent was removed under reduced pressure. Purification of the residue by flash column chromatography ($SiO_2$, $CH_2Cl_2$/MeOH 30:1) yielded **S16** as a colorless oil (0.20 g, 0.11 mmol, 80%).

**$^1$H NMR** (400 MHz, $CDCl_3$) δ 7.89 (s, 4H, $H_2$), 7.78 (s, 2H, $H_1$), 7.55 (d, *J* = 8.93 Hz, 4H, $H_5$), 7.24 (d, *J* = 8.27 Hz, 4H, $H_6$), 5.02 (s, 4H, $H_3$), 4.57 (t, *J* = 5.17 Hz, 4H, $H_7$), 3.99 (t, *J* = 4.68 Hz, 4H, $H_8$), 3.75 – 3.77 (m, 4H, $H_9$), 3.70 – 3.68 (m, 4H, $H_{10}$), 3.61 – 3.56 (m, 32H, $H_{11}$), 3.52 – 3.47 (m, 32H, $H_{11}$), 1.20 (s, 18H, $H_4$).

**$^{13}$C NMR** (101 MHz, $CDCl_3$) δ 173.0, 163.4, 153.7, 141.0, 131.8 (q, *J* = 33.3 Hz), 130.1, 128.5 (q, *J* = 3.9 Hz), 127.4, 123.4 (q, *J* = 272.6 Hz), 121.5 (m), 115.4, 83.5, 70.7, 70.5, 70.0, 69.8, 67.5, 48.6, 27.6.

**HRMS** ($ESI^+$) Calculated for $C_{82}H_{116}O_{27}N_2F_{12}Na$ $[M+Na]^+$ 1811.7466 found 1811.7437.

## 6.6 Synthesis of **S17**

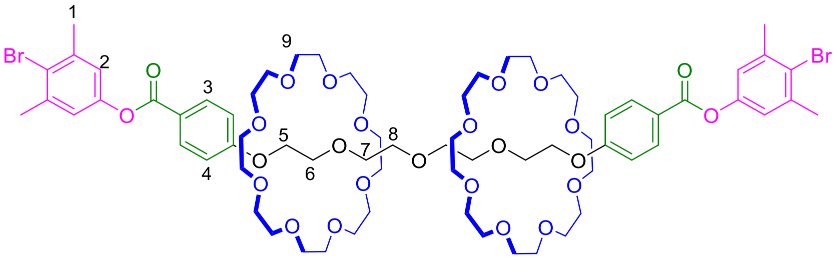

To a THF (1 ml) solution of **S16** (0.20 g, 0.11 mmol, 1.0 eq.) in a microwave vial were added 4-bromo-3,5-dimethyl phenol (0.067 g, 0.33 mmol, 3.0 eq.) and $K_3PO_4$ (0.068 g, 0.50 mmol, 4.5 eq.). The reaction mixture was sealed and stirred under microwave irradiation at 60 °C for 16 hours. The mixture was cooled to room temperature and the solvent was removed under reduced pressure. Purification of the residue by flash column chromatography ($SiO_2$, $CH_2Cl2$/MeOH 40:1) afforded **S17** as a colorless oil (0.089 mg, 0.058 mmol, 53%).

**$^1$H NMR** (600 MHz, $CDCl_3$) δ 8.12 (d, $J$ = 8.93 Hz, 4H, $H_3$), 7.39 (d, $J$ = 8.54 Hz, 4H, $H_4$), 6.98 (s, 4H, $H_2$), 4.65 (t, $J$ = 5.09 Hz, 4H, $H_5$), 4.02 (t, $J$ = 5.27 Hz, 4H, $H_6$), 3.79 – 3.76 (m, 4H, $H_7$), 3.75 – 3.72 (m, 4H, $H_8$), 3.66 – 3.61 (m, 32H, $H_9$), 3.55 – 3.50 (m, 32H, $H_9$), 2.45 (s, 12H, $H_1$).

**$^{13}$C NMR** (151 MHz, $CDCl_3$) δ 165.6, 164.5, 149.7, 139.5, 131.8, 124.0, 121.7, 120.3, 116.1, 70.7, 70.4, 69.98, 69.96, 67.3, 24.1.

**HRMS** (ESI$^+$) Calculated for $C_{70}H_{104}O_{25}Br_2Na$ $[M+Na]^+$ 1525.5126 found 1525.5069.

## 6.7 Synthesis of **16**

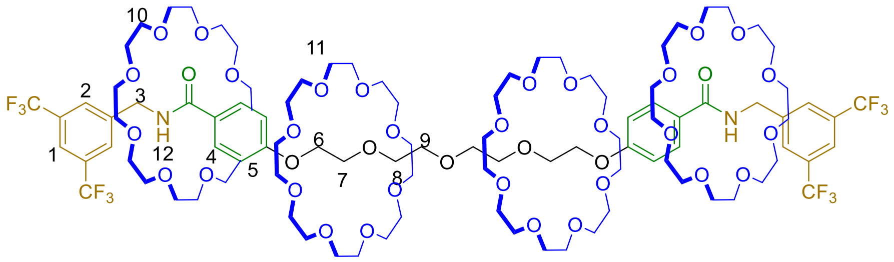

To a solution of **S17** (0.030 g, 0.019 mmol, 1.0 eq.) in toluene (1 mL) were added 24-C-8 **2** (0.037 mg, 0.10 mmol, 5.5 eq.) and 3,5-bis-trifluoromethylbenzylamine (0.013 g, 0.053 mmol, 2.8 eq.). The reaction mixture was stirred at room temperature for 21 days. The solvent was removed under reduced pressure and the residue was purified by flash column chromatography ($SiO_2$, $CH_2Cl_2$/MeOH 25:1) to afford **16** as a colorless oil (0.015 g, 6.5 µmol, 35%).

**$^1$H NMR** (600 MHz, $CDCl_3$) δ 8.74 (s, 4H, $H_2$), 7.98 (d, *J* = 9.17 Hz, 4H, $H_4$), 7.83 (br, 2H, $H_{12}$), 7.74 (s, 2H, $H_1$), 7.15 (d, *J* = 9.17 Hz, 4H, $H_5$), 4.94 (s, 4H, $H_3$), 4.50 (t, *J* = 5.40 Hz, 4H, $H_6$), 4.00 (t, *J* = 6.50 Hz, 4H, $H_7$), 3.76 (t, *J* = 5.40 Hz, 4H, $H_8$), 3.71 (t, *J* = 6.40 Hz, 4H, $H_9$), 3.62 – 3.57 (m, 32H, $H_{11}$), 3.55 – 3.51 (m, 32H, $H_{11}$), 3.43 (q, *J* = 6.82 Hz, 32H, $H_{10}$), 3.19 (q, *J* = 5.67 Hz, 32H, $H_{10}$).

**$^{13}$C NMR** (151 MHz, $CDCl_3$) δ 167.5, 161.8, 141.3, 135.6, 129.6, 129.0 (q, *J* = 32.5 Hz), 126.7, 124.3 (q, *J* = 272.4 Hz), 119.8, 114.8, 70.6, 70.4, 70.3, 69.94, 69.90, 67.2, 44.8.

**HRMS** (ESI$^+$) Calculated for $C_{104}H_{163}O_{39}N_2F_{12}$ $[M{-}H]^-$ 2292.0636 found 2292.0610.

# 8 Single Crystal X-Ray Diffraction

**Data Collection.** X-Ray data for **16** were collected at beamline I19 in Diamond Light Source synchrotron.[S1] Data were measured using GDA suite of programs.

**Crystal structure determinations and refinements.** X-Ray data were processed and reduced using CrysAlisPro suite of programmes. Absorption correction was performed using empirical methods (SCALE3 ABSPACK) based upon symmetry-equivalent reflections combined with measurements at different azimuthal angles. The crystal structure was solved and refined against all $F^2$ values using the SHELX and Olex 2 suite of programmes.[S2] Despite of the usage of a highly intense X-ray source, crystals of **16** only diffracted to 1 Å of resolution. X-ray data were found to be modulated with a q vector of (0, 0, ½). The phase problem was solved using C2 space group with the small unit cell setting. Diffraction data were also processed using the supercell configuration, but the phase problem couldn't be solved using either SHELX or Jana suit of programs. The crystal structure of **16** in space group C2 presented a large amount of disorder, caused by the data modulation. The disorder was modelled over two positions, where atomic distances were restrained using distance restrains (SHELX; DFIX and SADI). The atomic displacement parameters (adp) of the threads and the macrocycles were restrained using rigid body restrains (SHELX RIGU and SIMU commands). In order to maximize the data/parameter ratio, a large amount of disordered moieties were refined isotropically. Hydrogen atoms were placed in the calculated positions.

Solvent mask protocol in Olex 2 was used to account with the remaining electron density finding 8 electrons, which could correspond to a water molecule.

**a)**

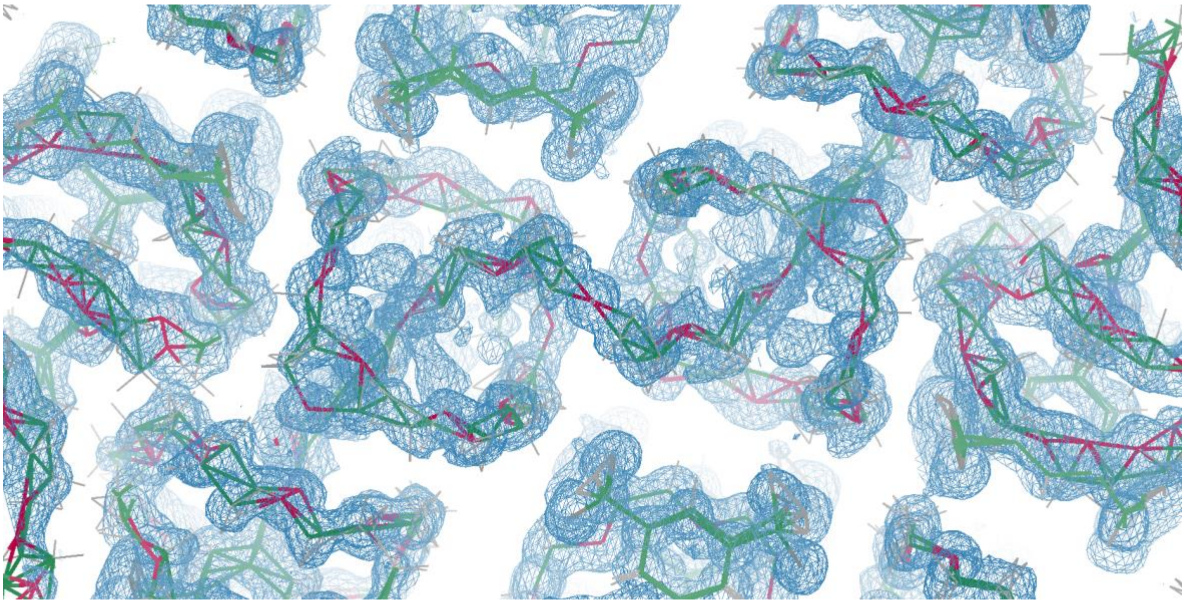

**b)**

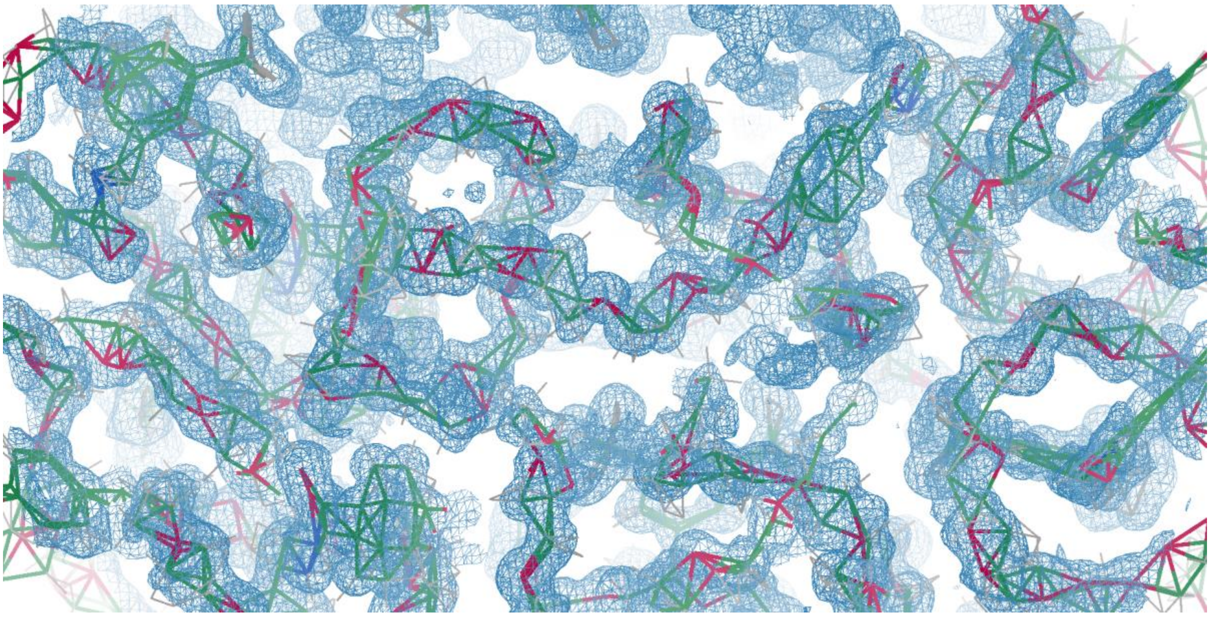

**Figure 1**. Electron density maps with the disordered structure model. Oxygens in red, carbons in green, nitrogen in blue and fluorine in white. Hydrogen atoms were omitted for clarity.[S3]

| | |
|---|---|
| Identification code | **16** |
| Empirical formula | $C_{156}H_{247.51}F_{18}N_3O_{59.26}$ |
| Formula weight | 3455.19 |
| Temperature/K | 100 |
| Crystal system | monoclinic |
| Space group | C2 |
| a/Å | 55.0452(12) |
| b/Å | 9.07320(17) |
| c/Å | 36.6583(9) |
| α/° | 90 |

| | |
|---|---|
| β/° | 106.624(2) |
| γ/° | 90 |
| Volume/Å$^3$ | 17543.2(7) |
| Z | 4 |
| $\rho_{calc}$g/cm$^3$ | 1.308 |
| μ/mm$^{-1}$ | 0.104 |
| F(000) | 7362.0 |
| Crystal size/mm$^3$ | 0.13 × 0.02 × 0.02 |
| Radiation | Synchrotron (λ = 0.6889) |
| 2Θ range for data collection/° | 2.88 to 40.294 |
| Index ranges | -54 ≤ h ≤ 54, -9 ≤ k ≤ 9, -36 ≤ l ≤ 36 |
| Reflections collected | 28884 |
| Independent reflections | 17297 [$R_{int}$ = 0.0361, $R_{sigma}$ = 0.0687] |
| Data/restraints/parameters | 17297/29176/2536 |
| Goodness-of-fit on F$^2$ | 2.314 |
| Final R indexes [I>=2σ (I)] | $R_1$ = 0.1890, $wR_2$ = 0.4822 |
| Final R indexes [all data] | $R_1$ = 0.1962, $wR_2$ = 0.4896 |
| Largest diff. peak/hole / e Å$^{-3}$ | 1.27/-0.76 |

CCDC 2168088 contains the supplementary crystallographic data for this paper. These data can be obtained free of charge via www.ccdc.cam.ac.uk/conts/retrieving.html (or from the Cambridge Crystallographic Data Centre, 12 Union Road, Cambridge CB21EZ, UK; fax: (+44)1223-336-033; or deposit@ccdc.cam.ac.uk).

# 9 NMR Spectra

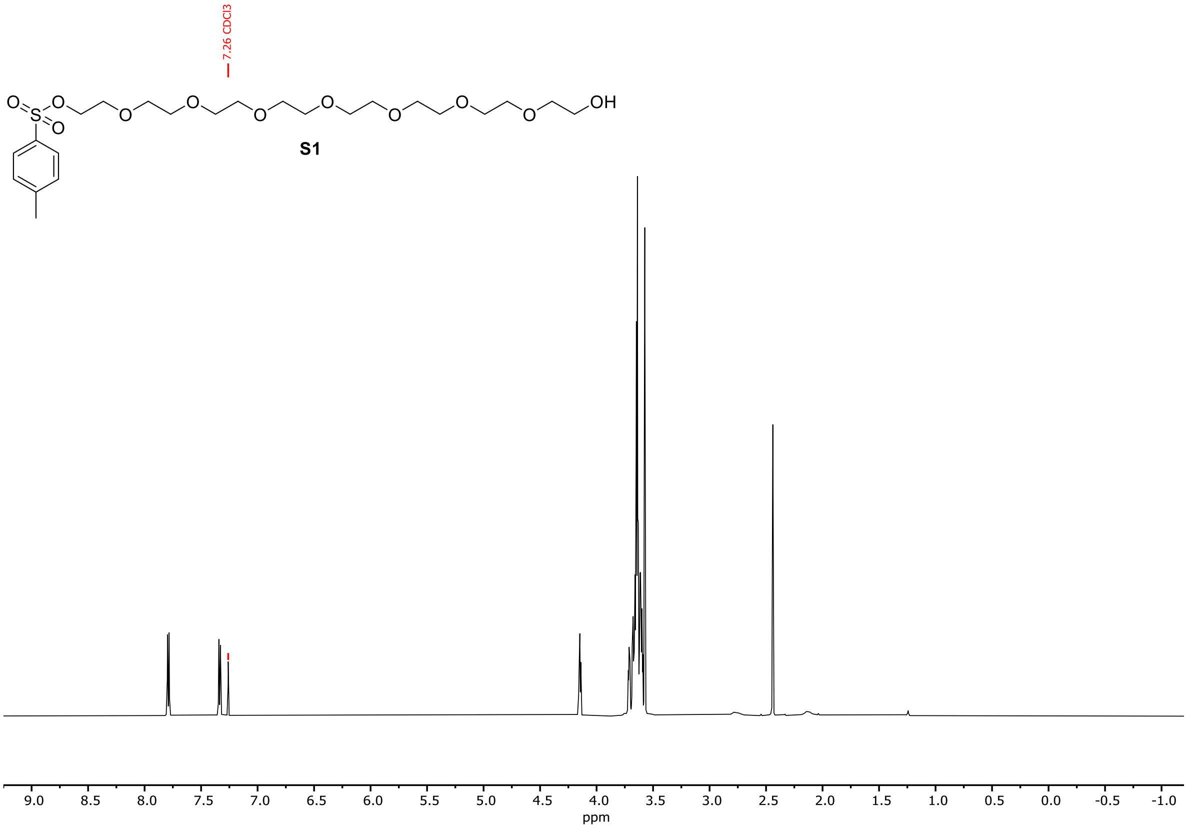


**Spectrum S1**: $^{1}H$ NMR ($CDCl_3$, 600 MHz) of **S1**.

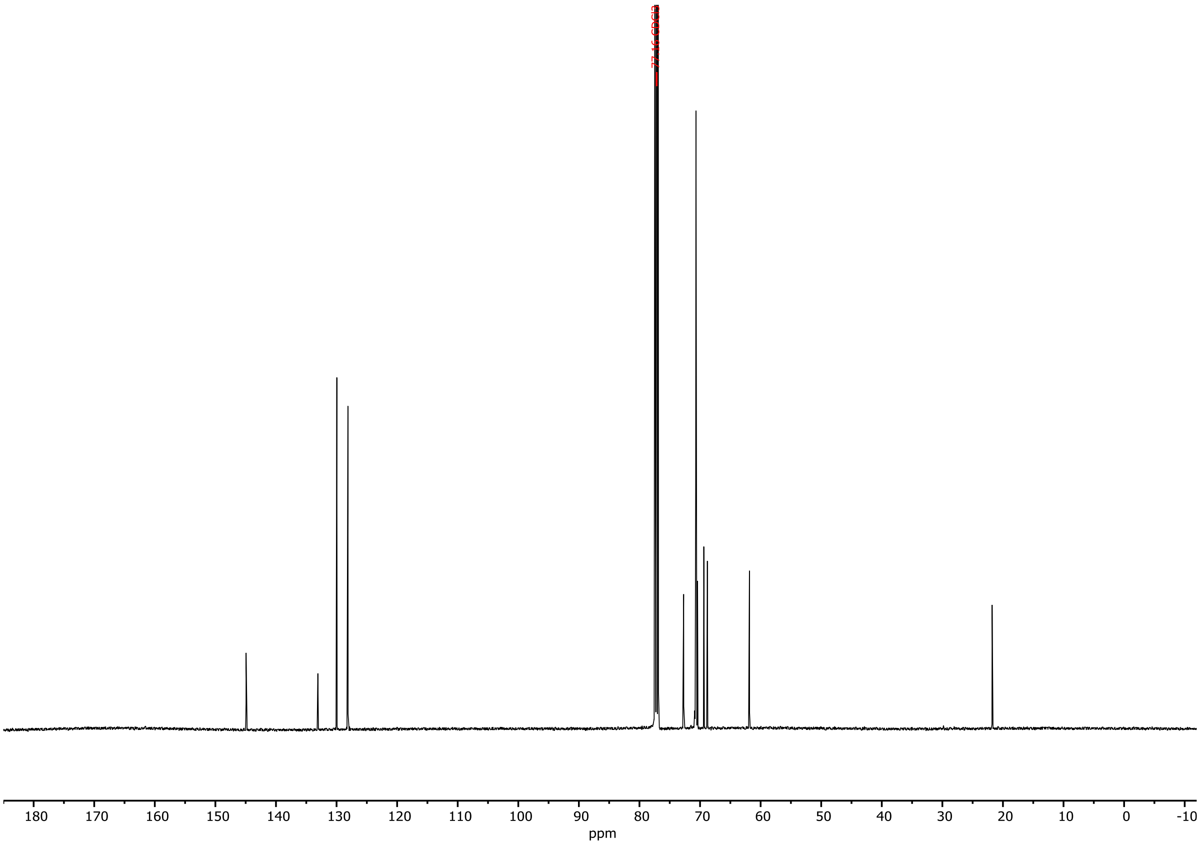


**Spectrum S2**: $^{13}C$ NMR ($CDCl_3$, 151 MHz) of **S1**.

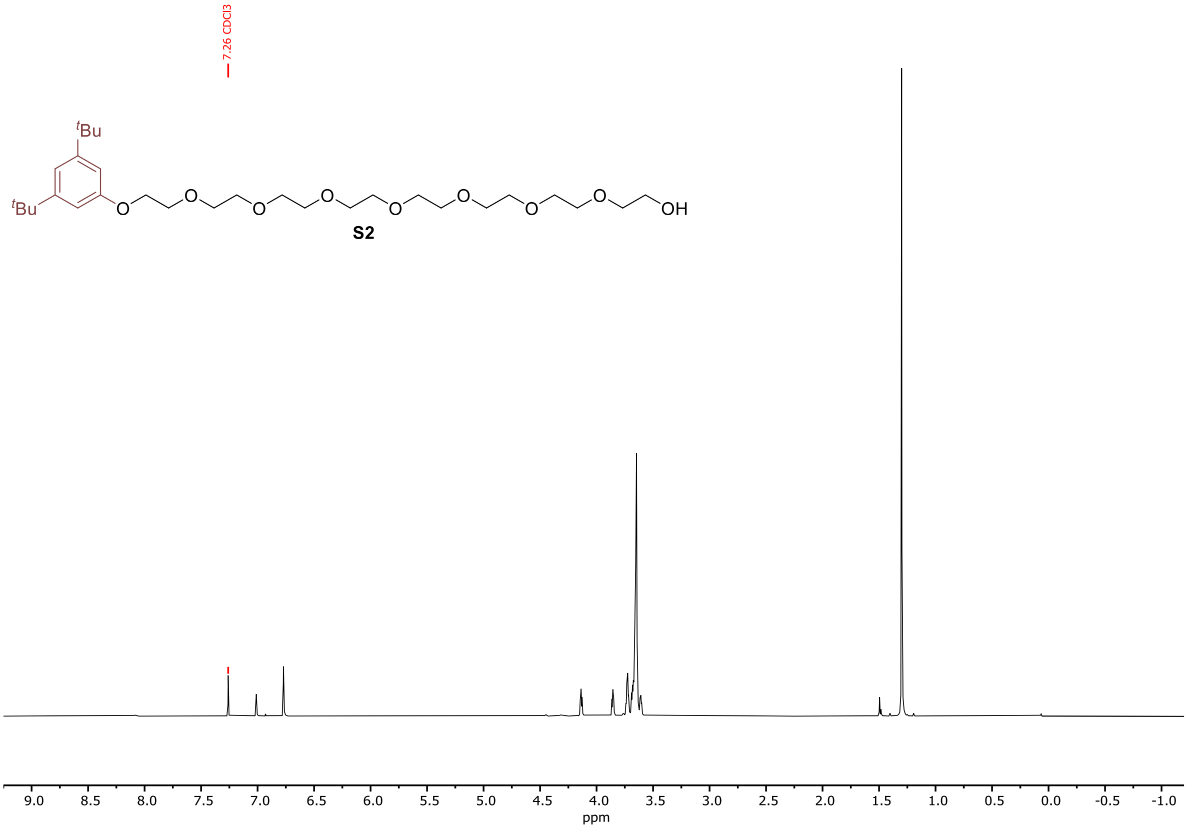


**Spectrum S3**: $^{1}H$ NMR ($CDCl_3$, 600 MHz) of **S2**.

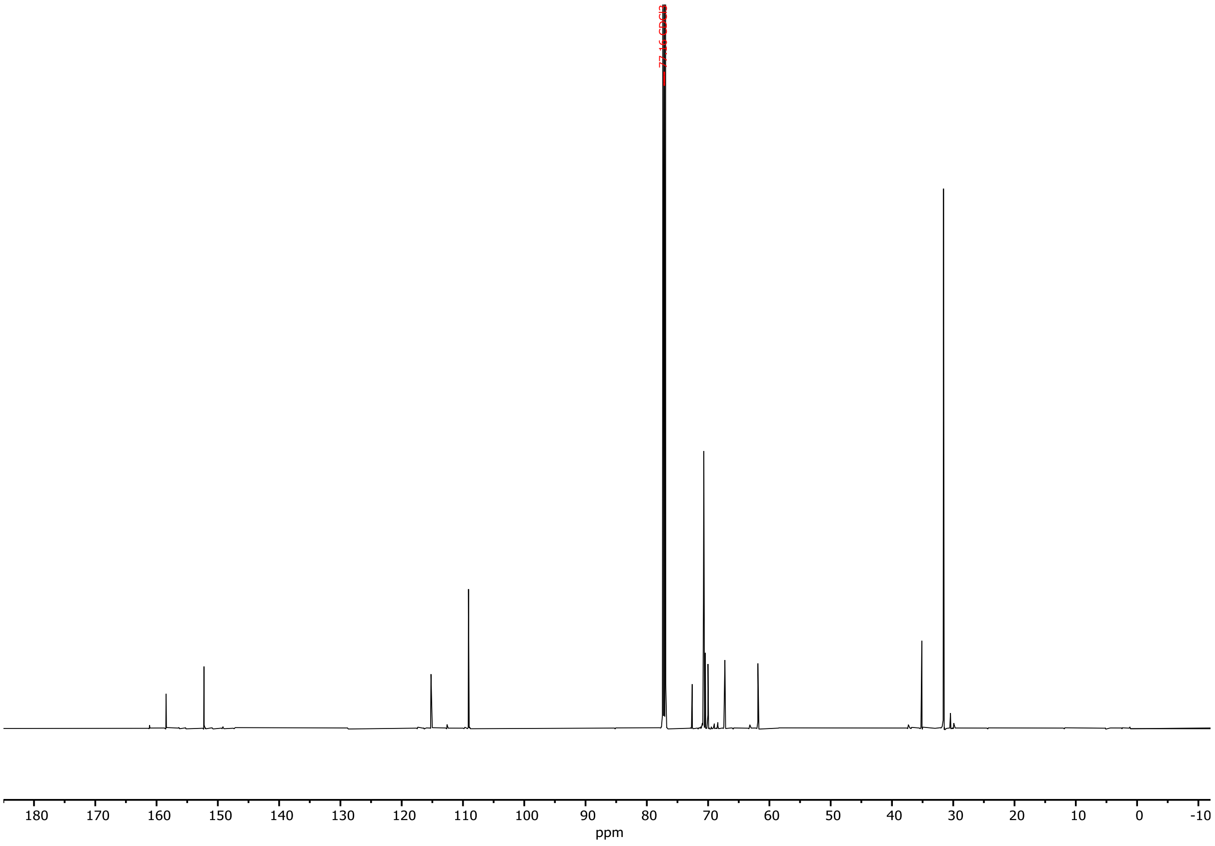


**Spectrum S4**: $^{13}C$ NMR ($CDCl_3$, 151 MHz) of **S2**.

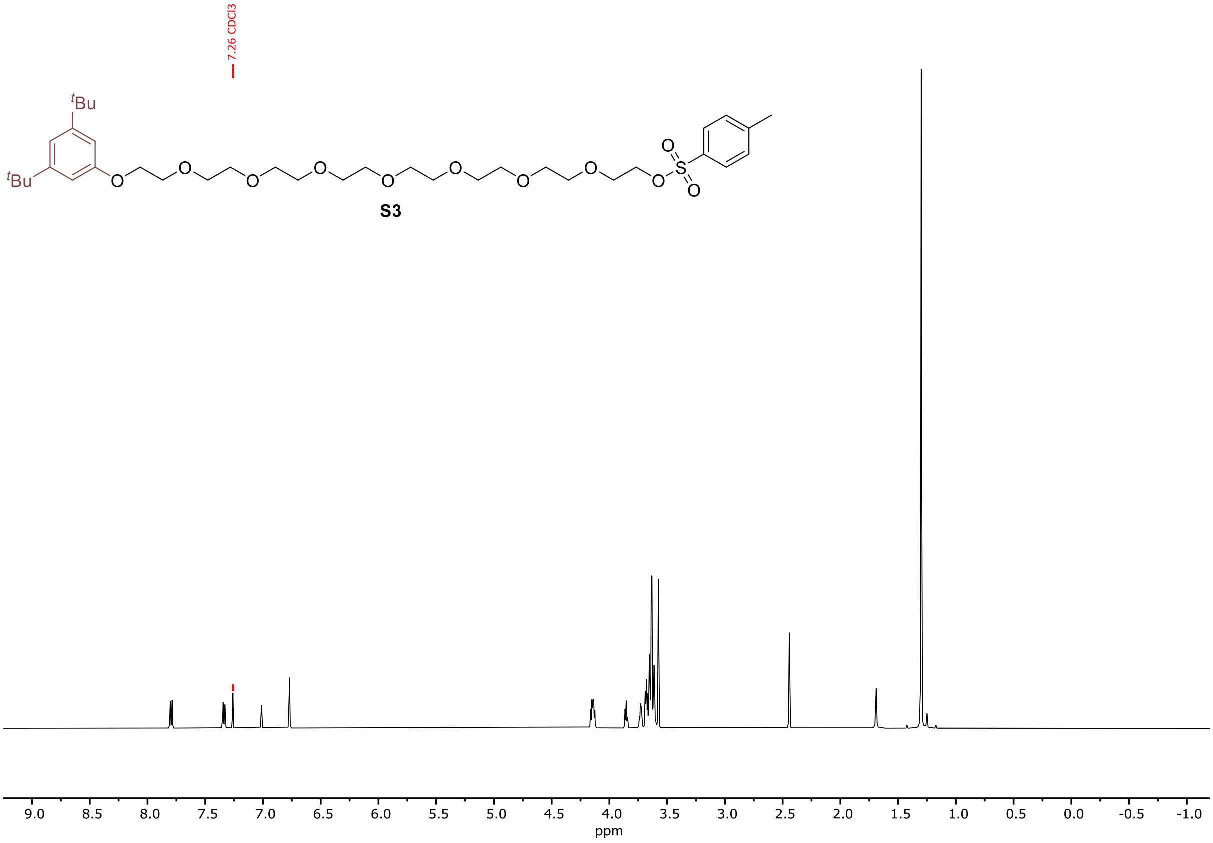


**Spectrum S5**: $^{1}H$ NMR ($CDCl_3$, 500 MHz) of **S3**.

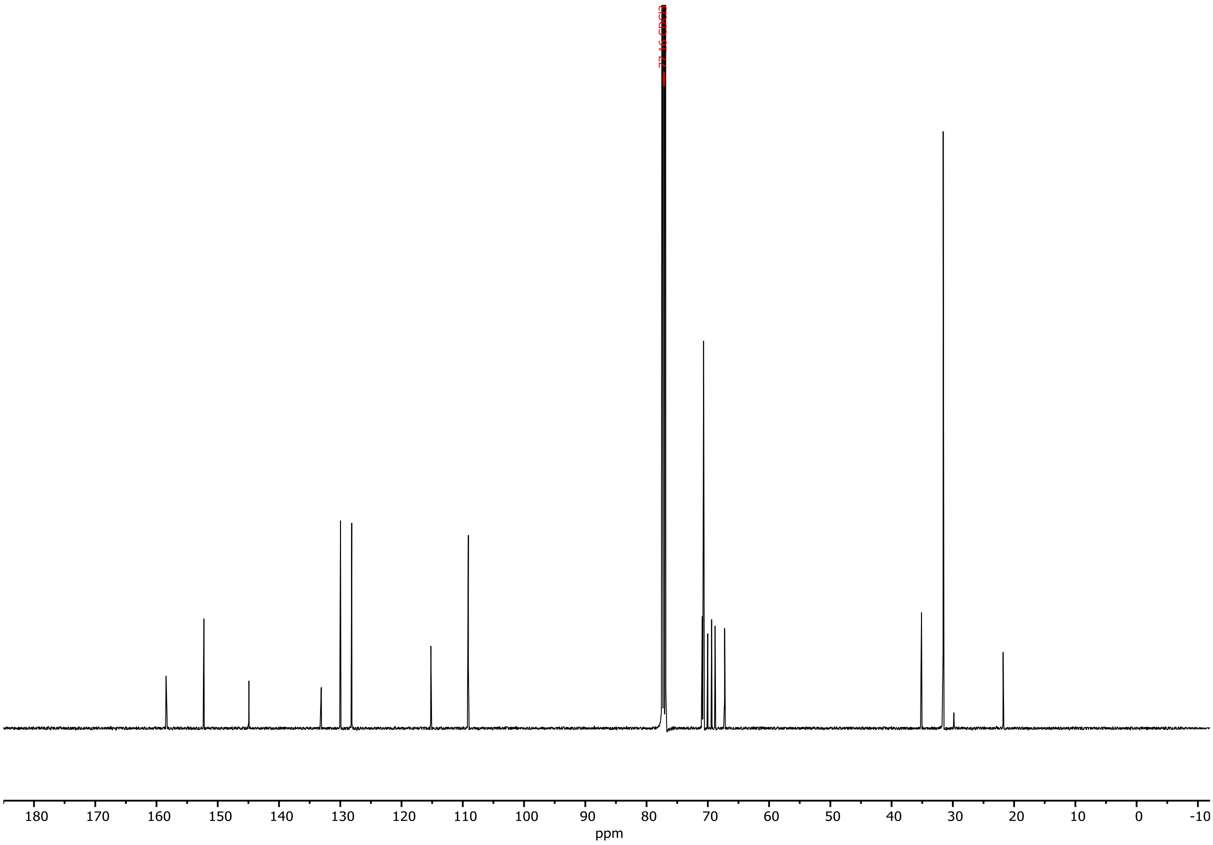


**Spectrum S6**: $^{13}C$ NMR ($CDCl_3$, 126 MHz) of **S3**.

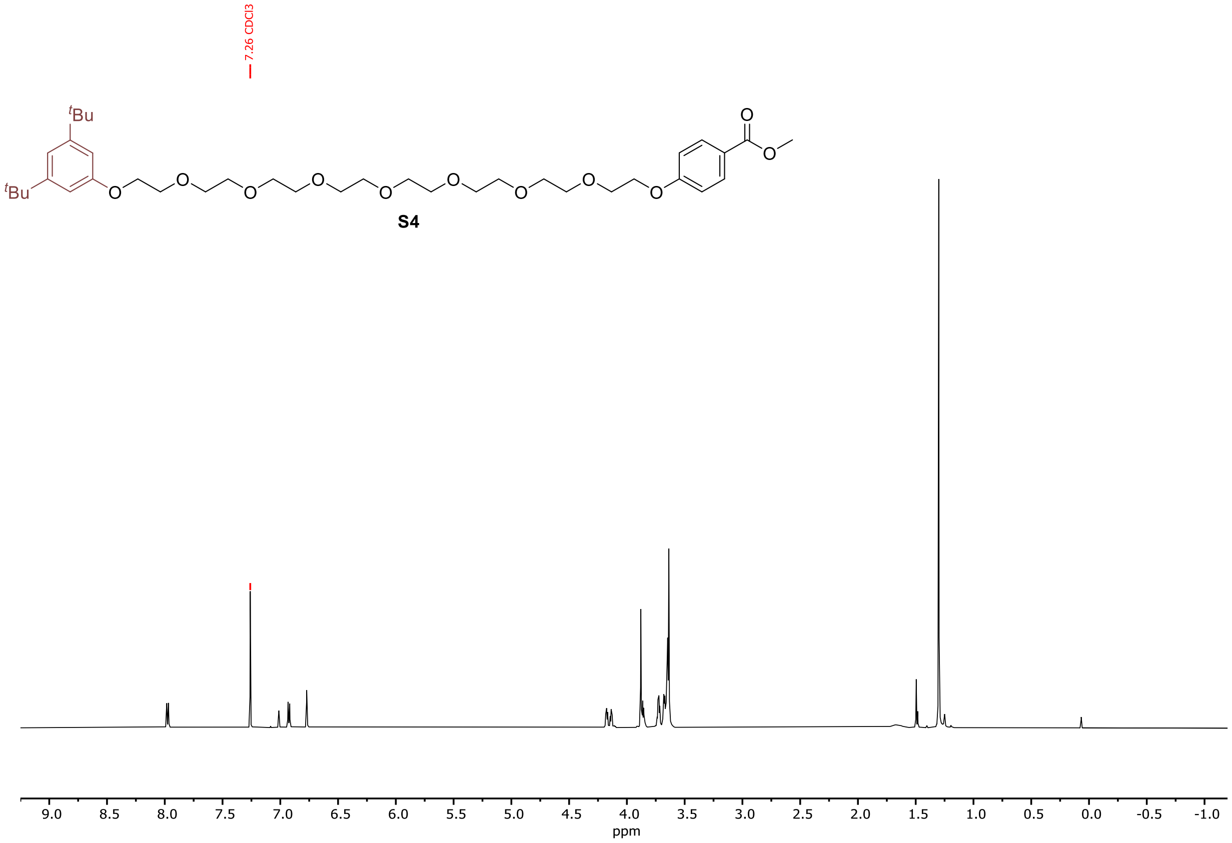


**Spectrum S7**: $^{1}H$ NMR ($CDCl_3$, 600 MHz) of **S4**.

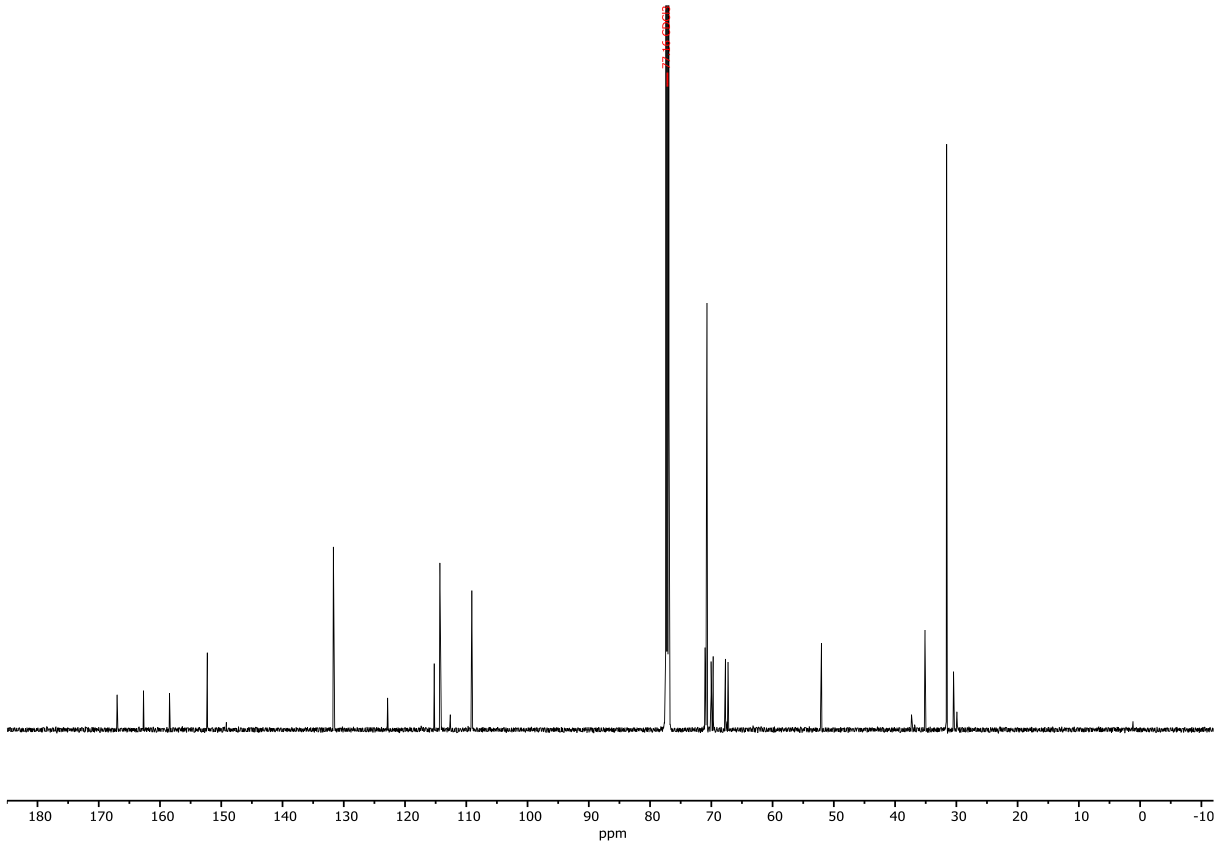

**Spectrum S8**: $^{13}C$ NMR ($CDCl_3$, 151 MHz) of **S4**.

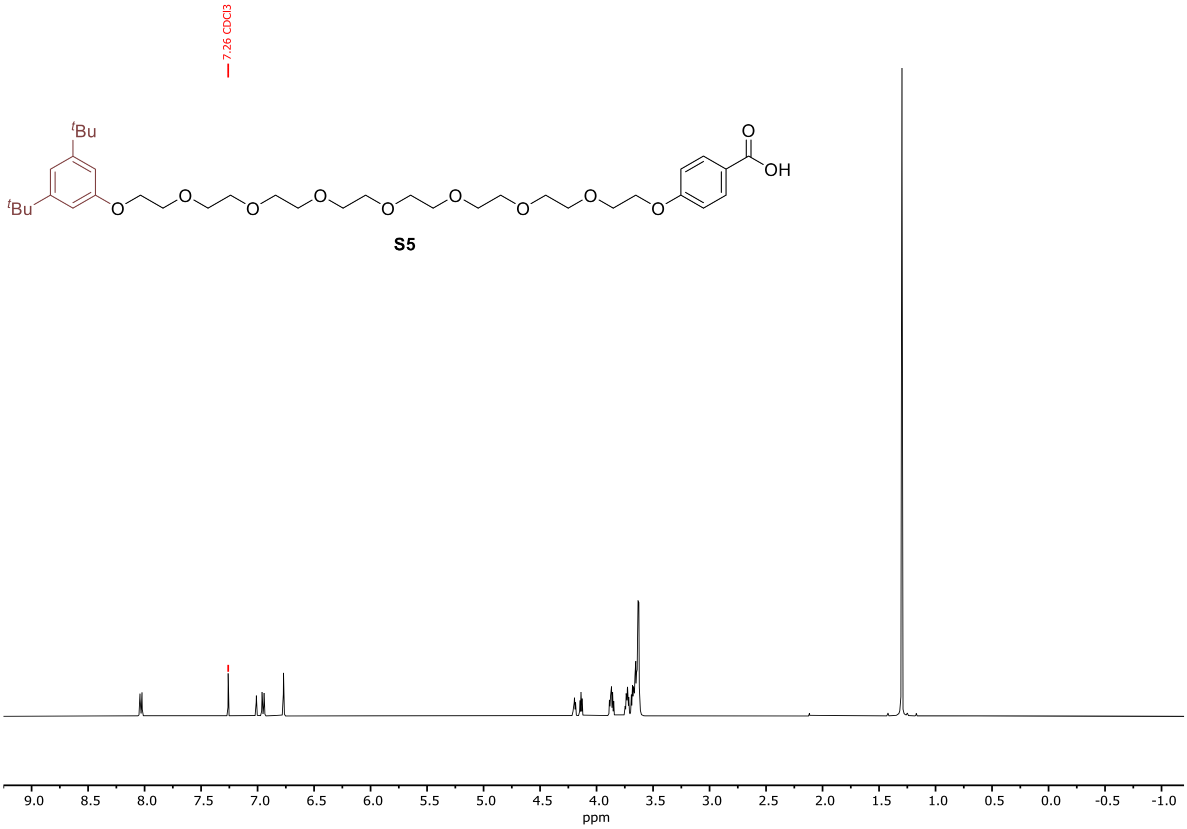


**Spectrum S9**: $^{1}$H NMR ($CDCl_3$, 500 MHz) of **S5**.

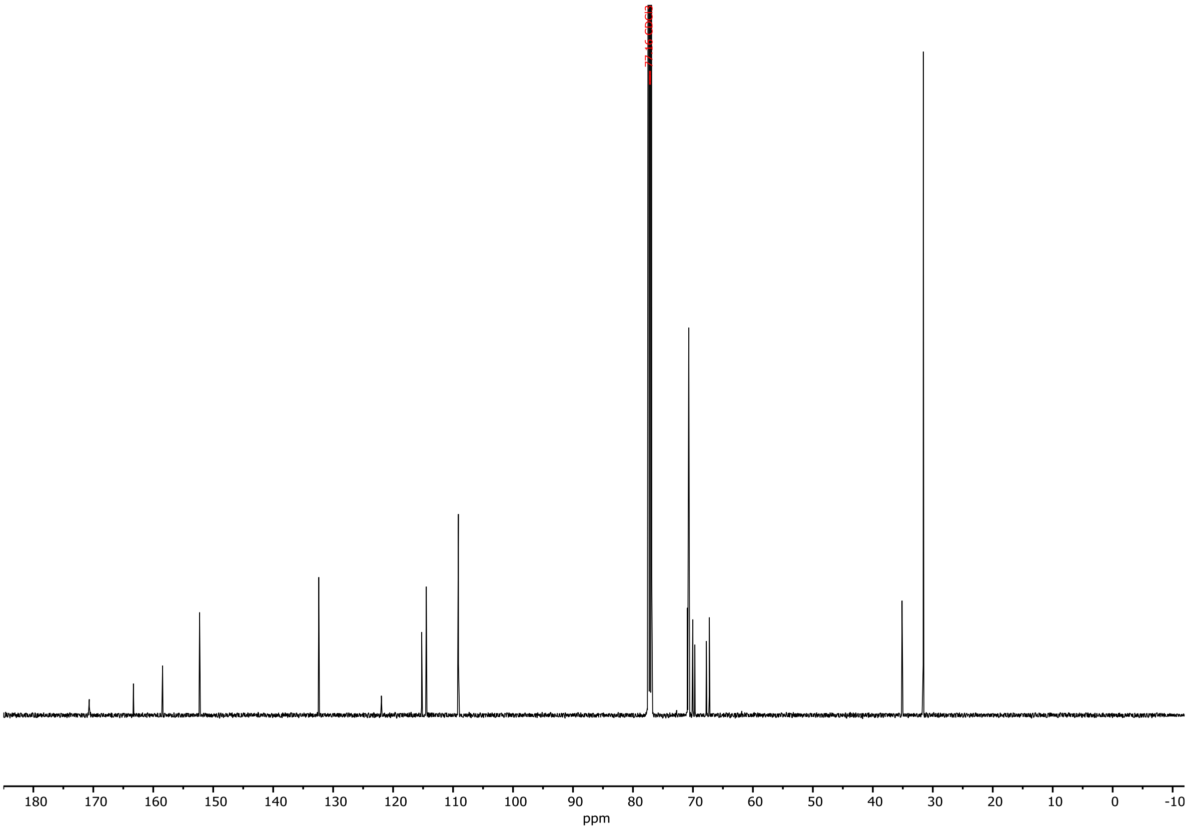


**Spectrum S10**: $^{13}$C NMR ($CDCl_3$, 126 MHz) of **S5**.

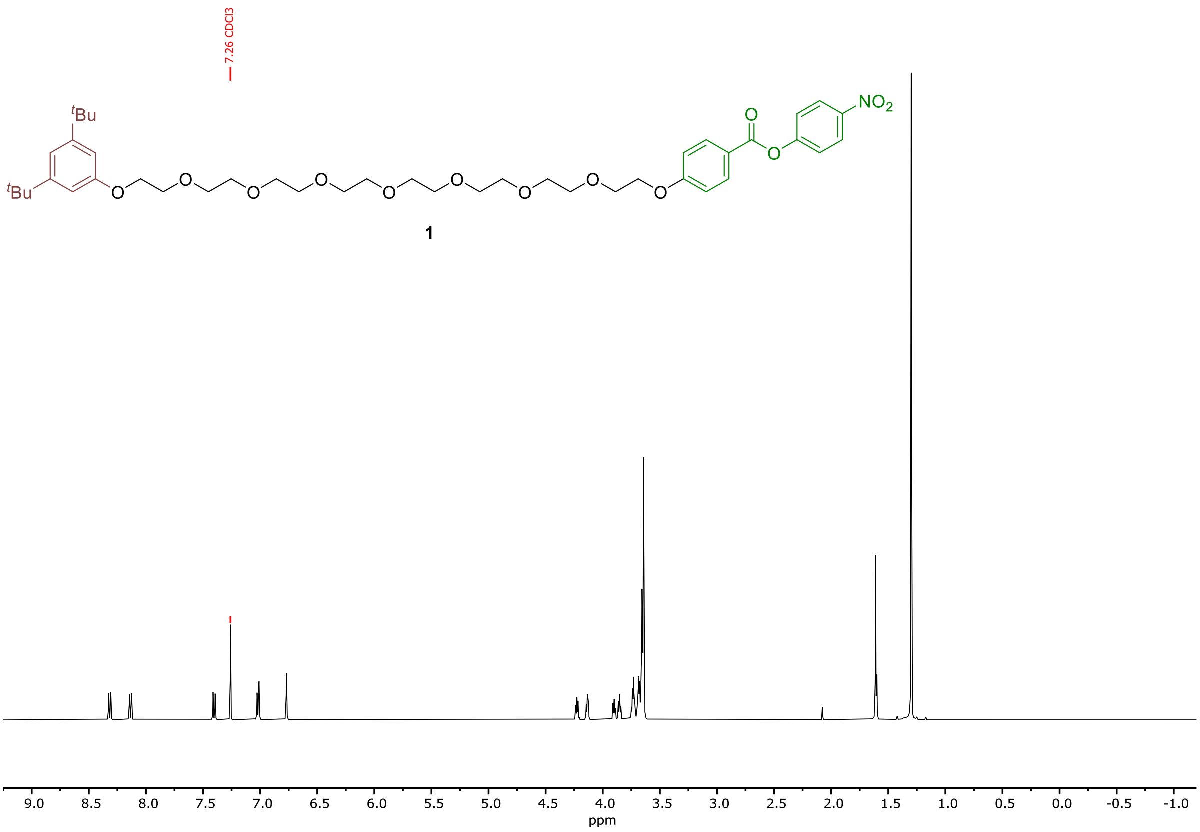


**Spectrum S11**: $^{1}H$ NMR ($CDCl_3$, 500 MHz) of **1**.

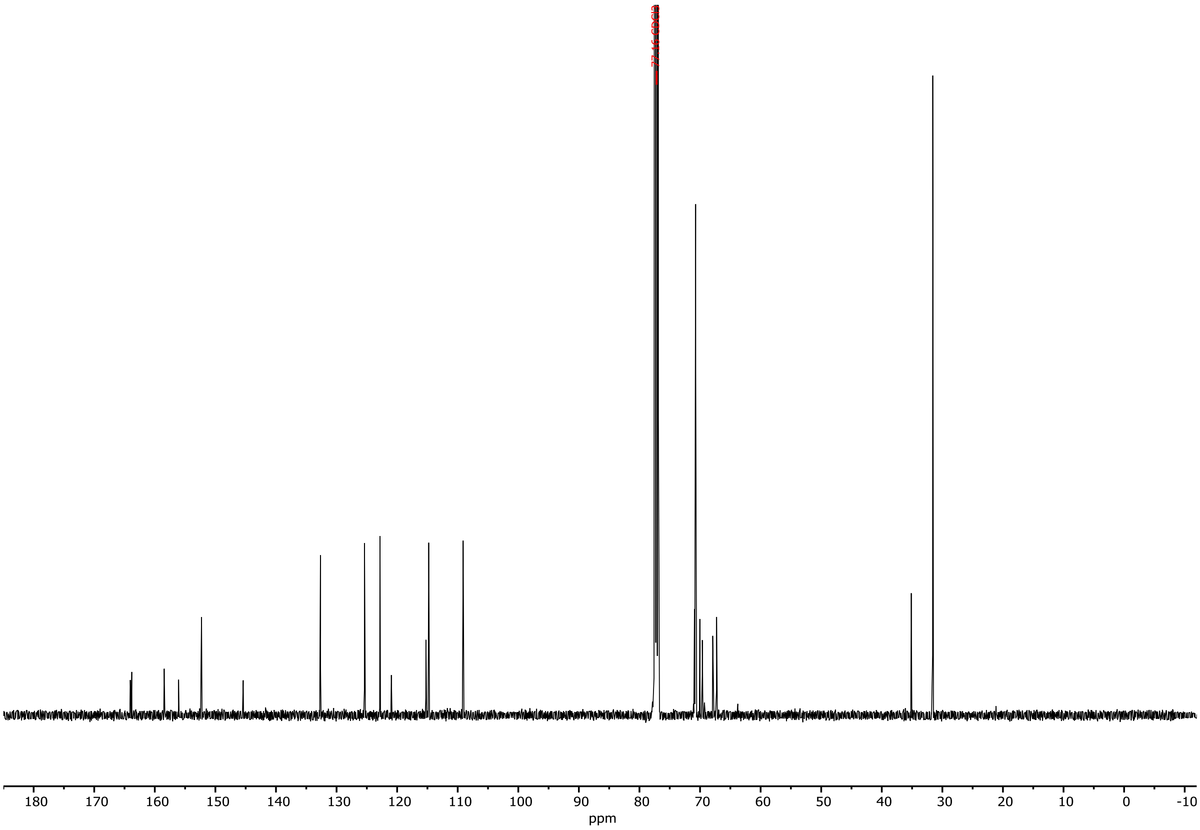


**Spectrum S12**: $^{13}C$ NMR ($CDCl_3$, 126 MHz) of **1**.

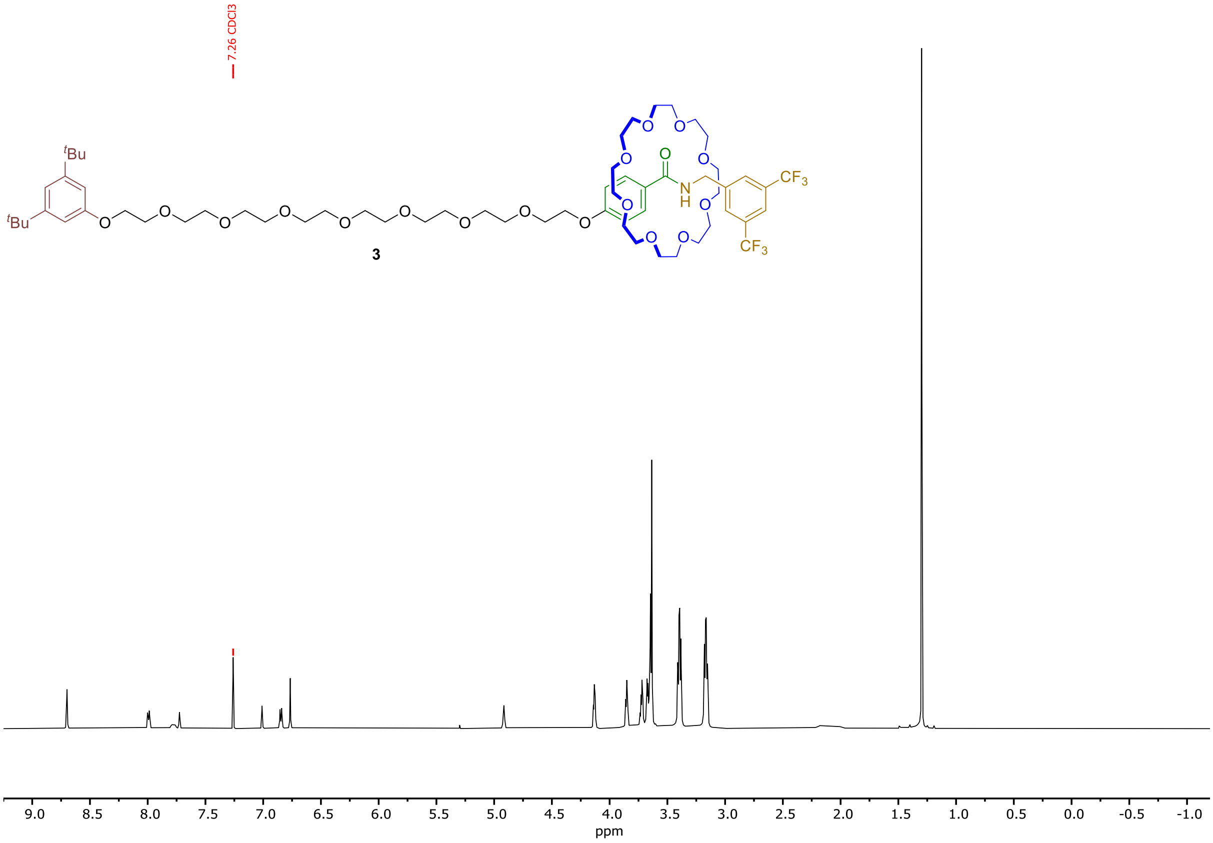


**Spectrum S13**: $^{1}H$ NMR ($CDCl_3$, 600 MHz) of **3**.

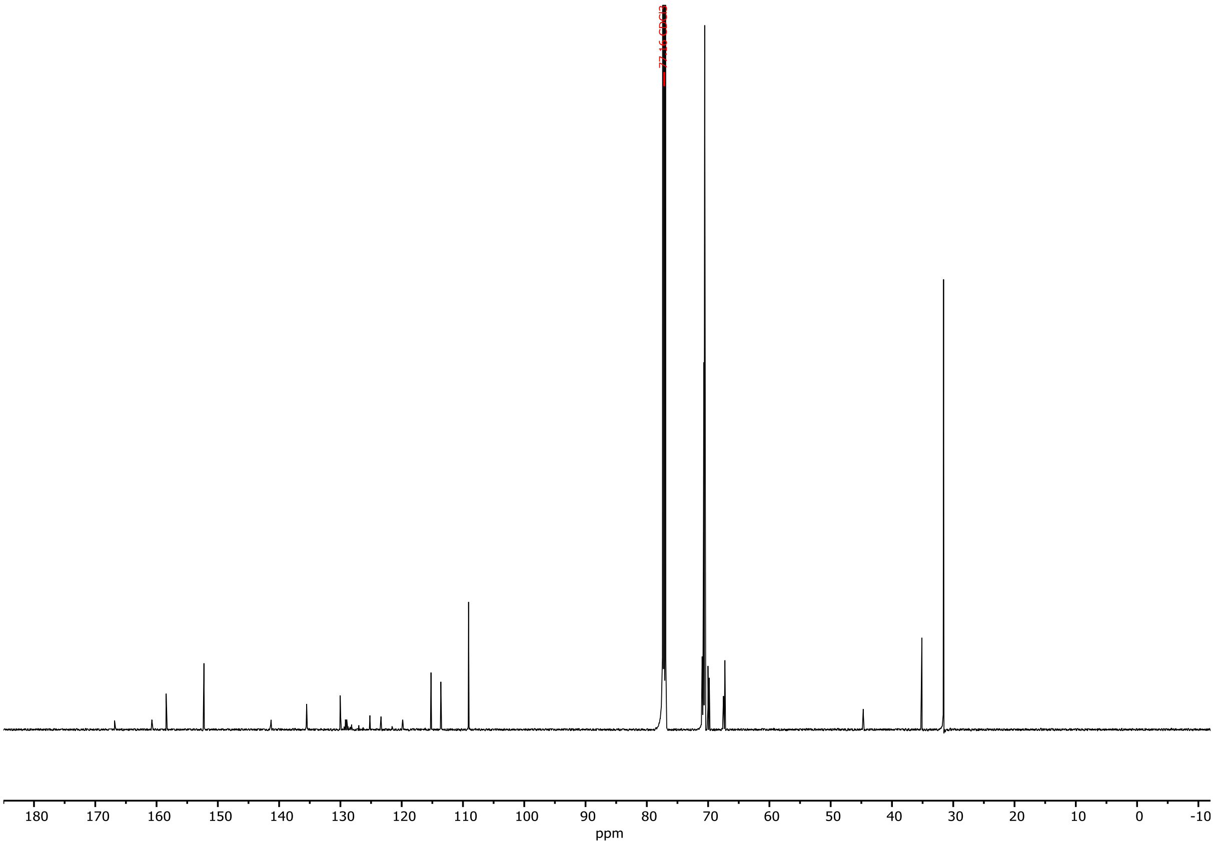


**Spectrum S14**: $^{13}C$ NMR ($CDCl_3$, 151 MHz) of **3**.

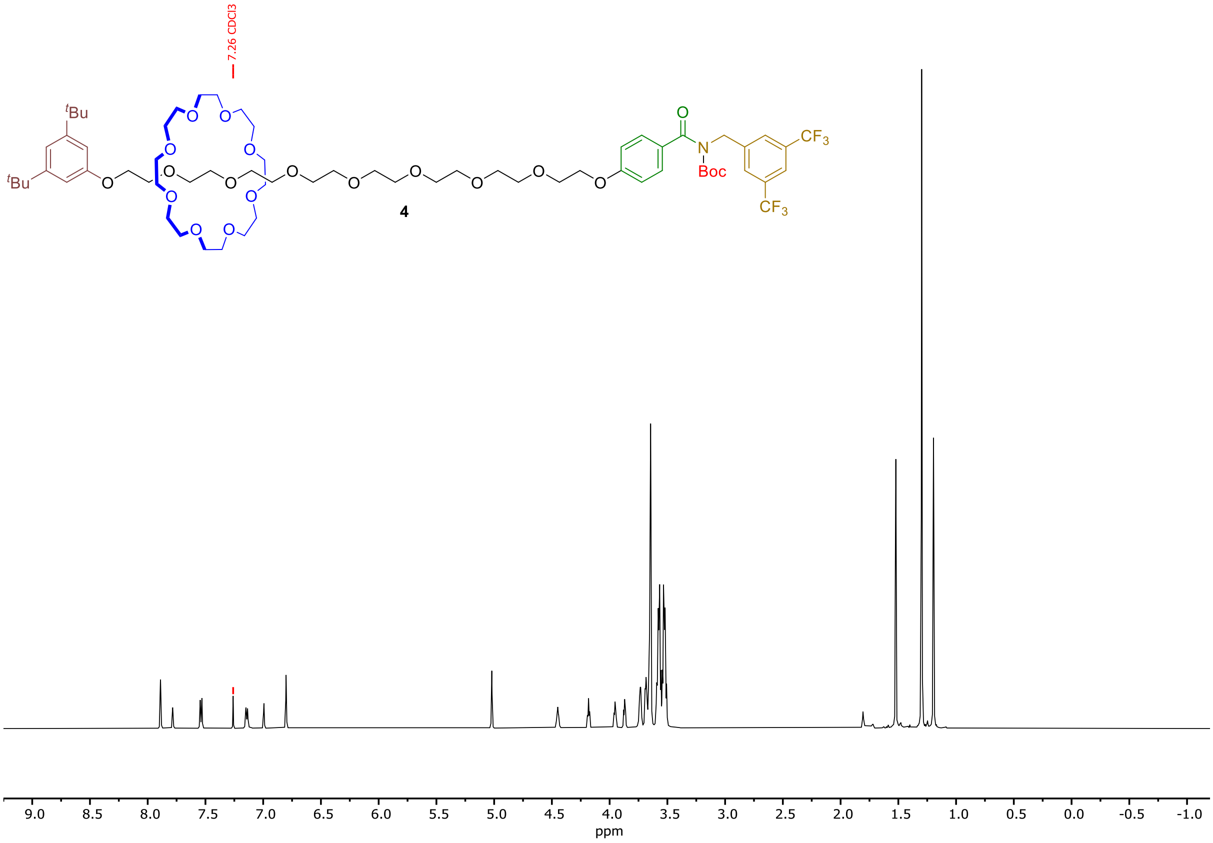


**Spectrum S15**: $^{1}H$ NMR ($CDCl_3$, 600 MHz) of **4**.

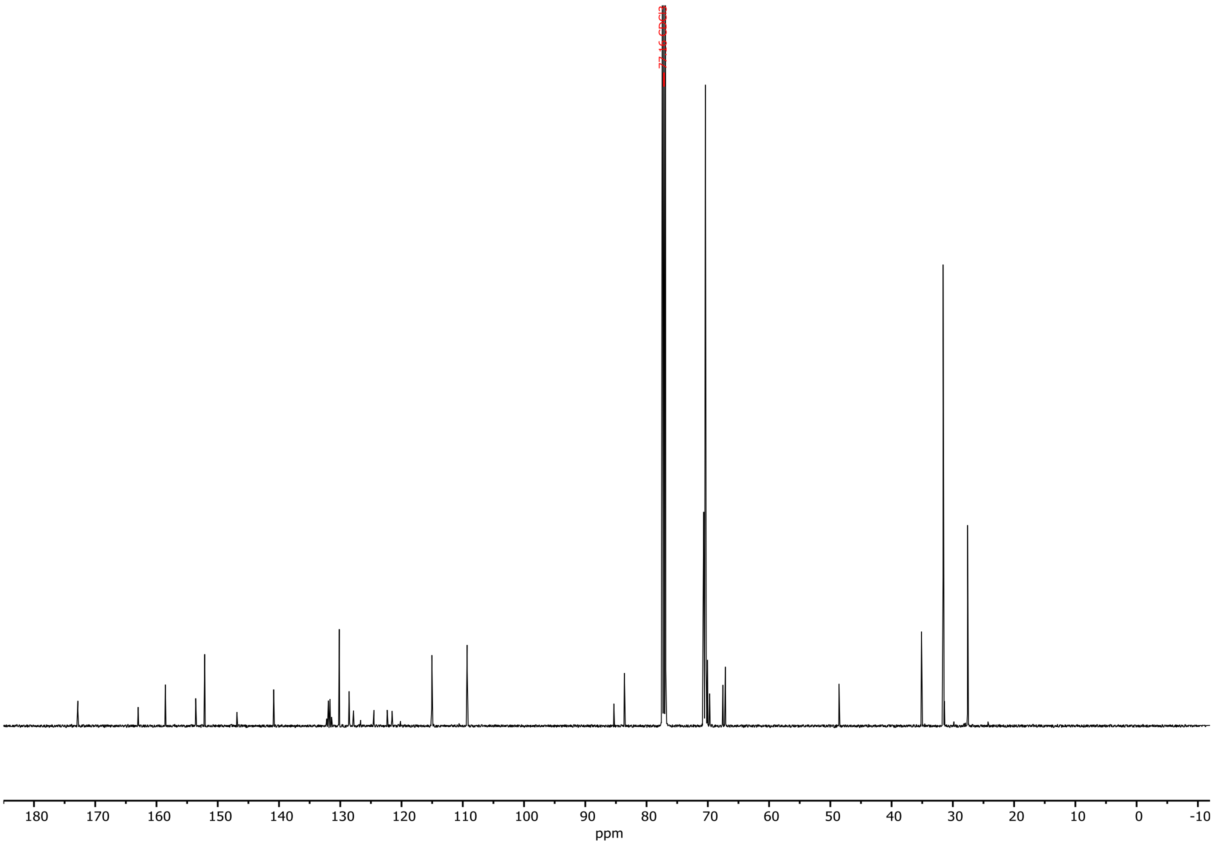


**Spectrum S16**: $^{13}C$ NMR ($CDCl_3$, 126 MHz) of **4**.

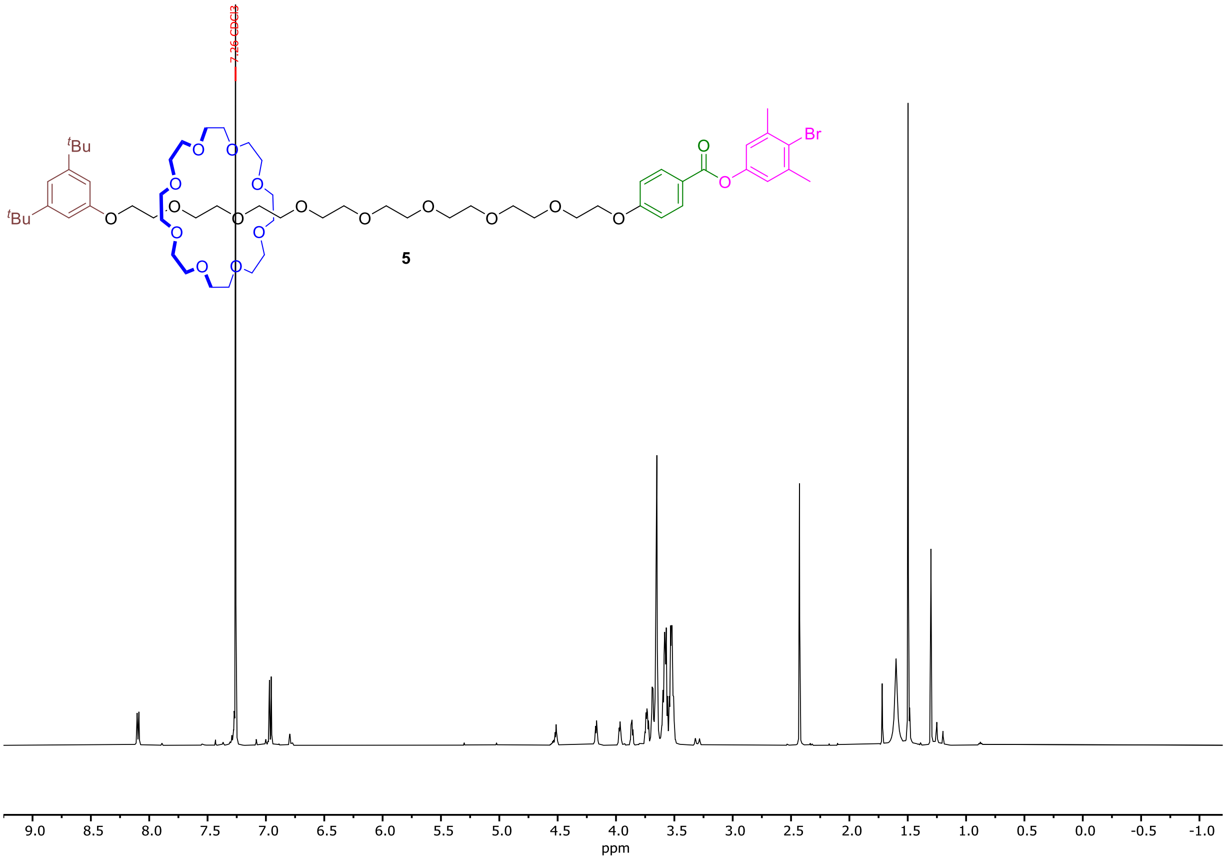


**Spectrum S17**: $^{1}H$ NMR ($CDCl_3$, 600 MHz) of **5**.

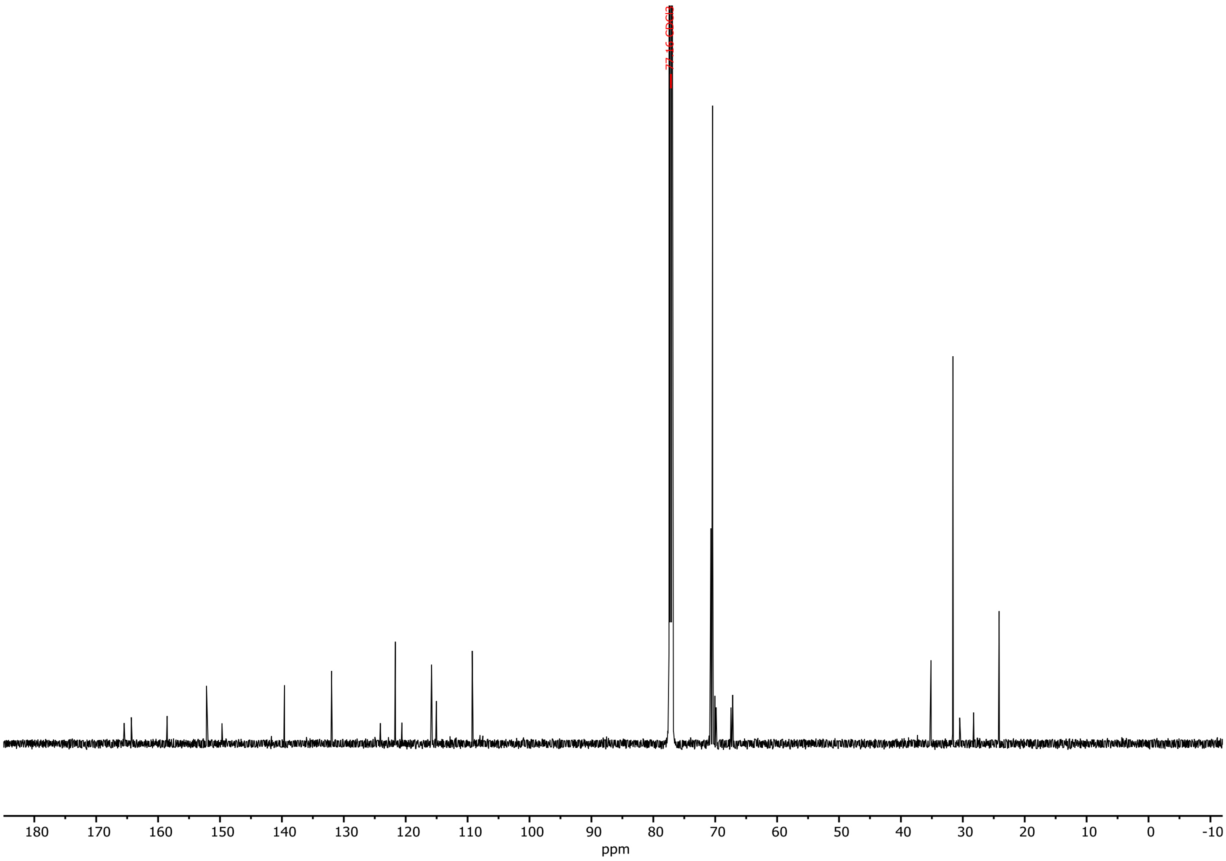


**Spectrum S18**: $^{13}C$ NMR ($CDCl_3$, 151 MHz) of **5**.

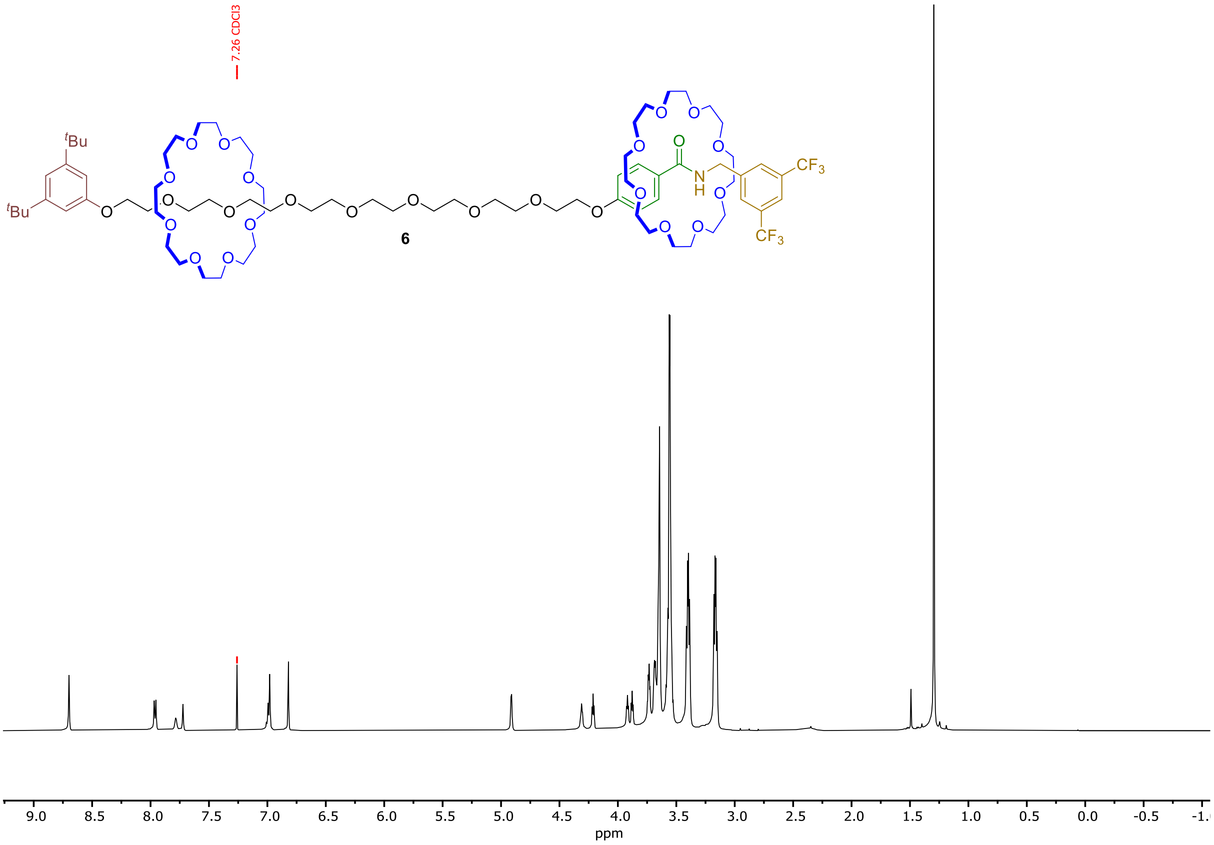


**Spectrum S19**: $^{1}H$ NMR ($CDCl_3$, 600 MHz) of **6**.

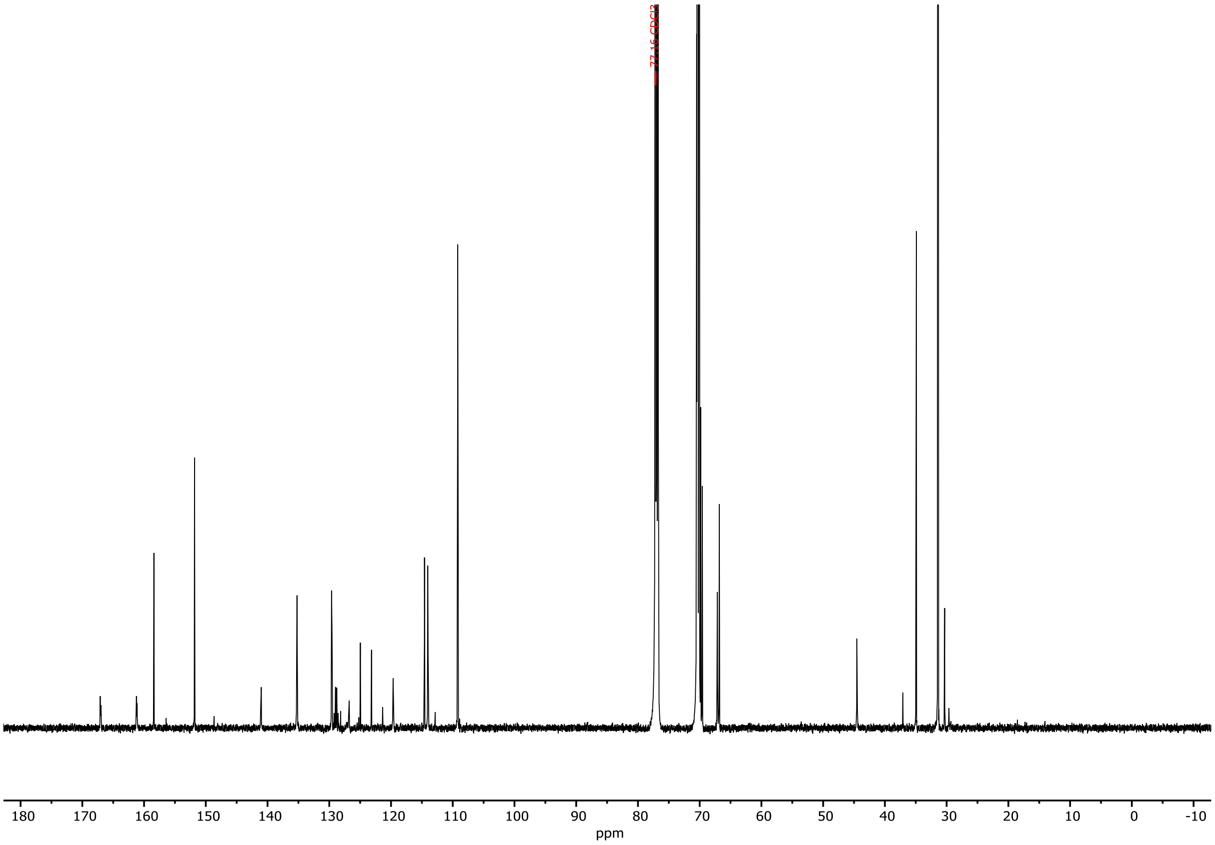


**Spectrum S20**: $^{13}C$ NMR ($CDCl_3$, 151 MHz) of **6**.

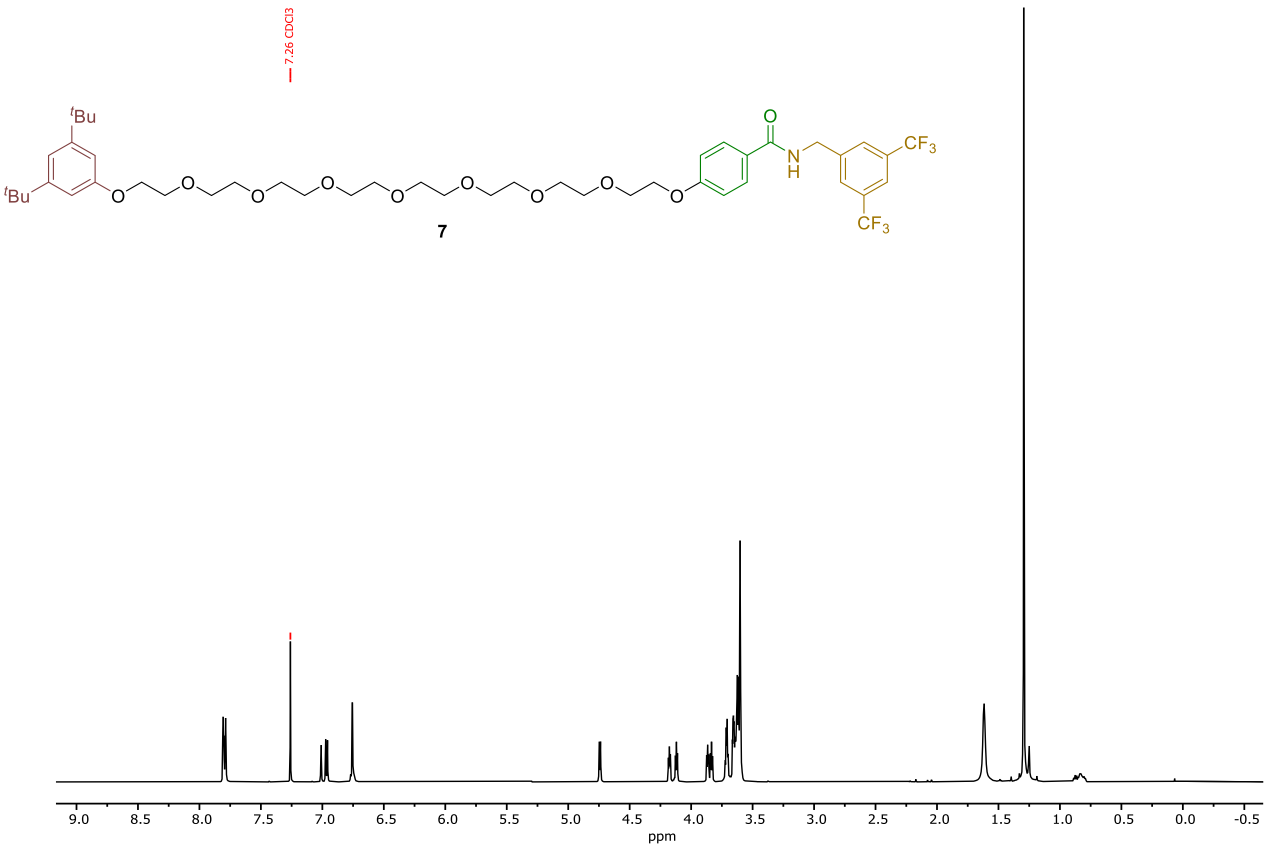


**Spectrum S21**: $^{1}H$ NMR ($CDCl_3$, 600 MHz) of **7**.

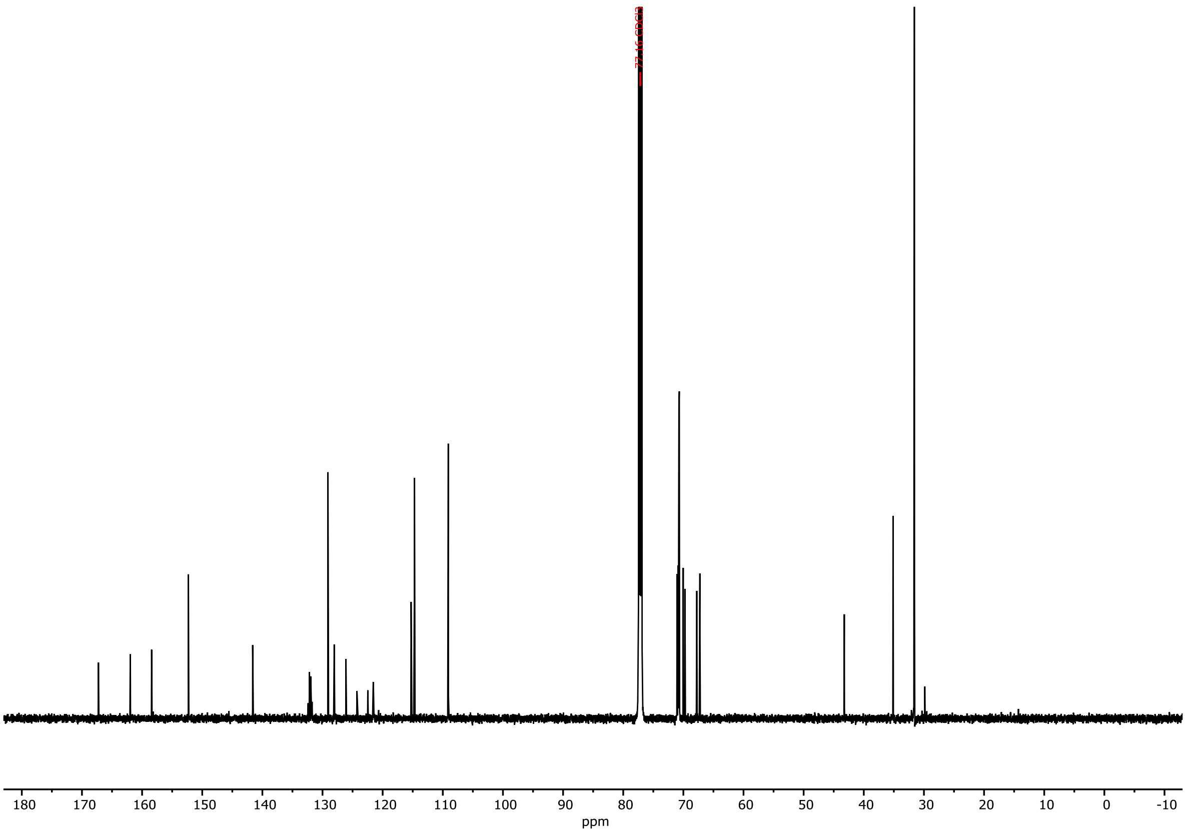


**Spectrum S22**: $^{13}C$ NMR ($CDCl_3$, 151 MHz) of **7**.

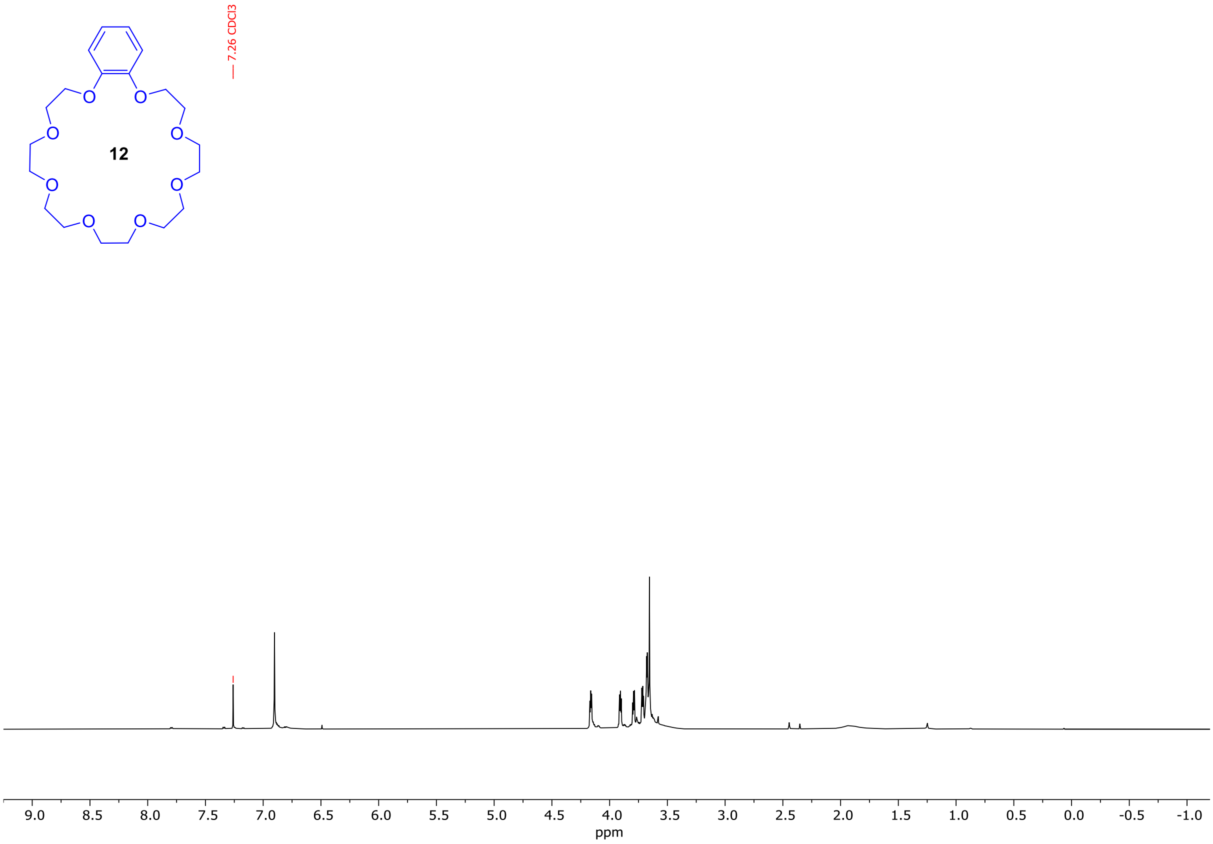


**Spectrum S23**: $^{1}H$ NMR ($CDCl_3$, 600 MHz) of **12**.

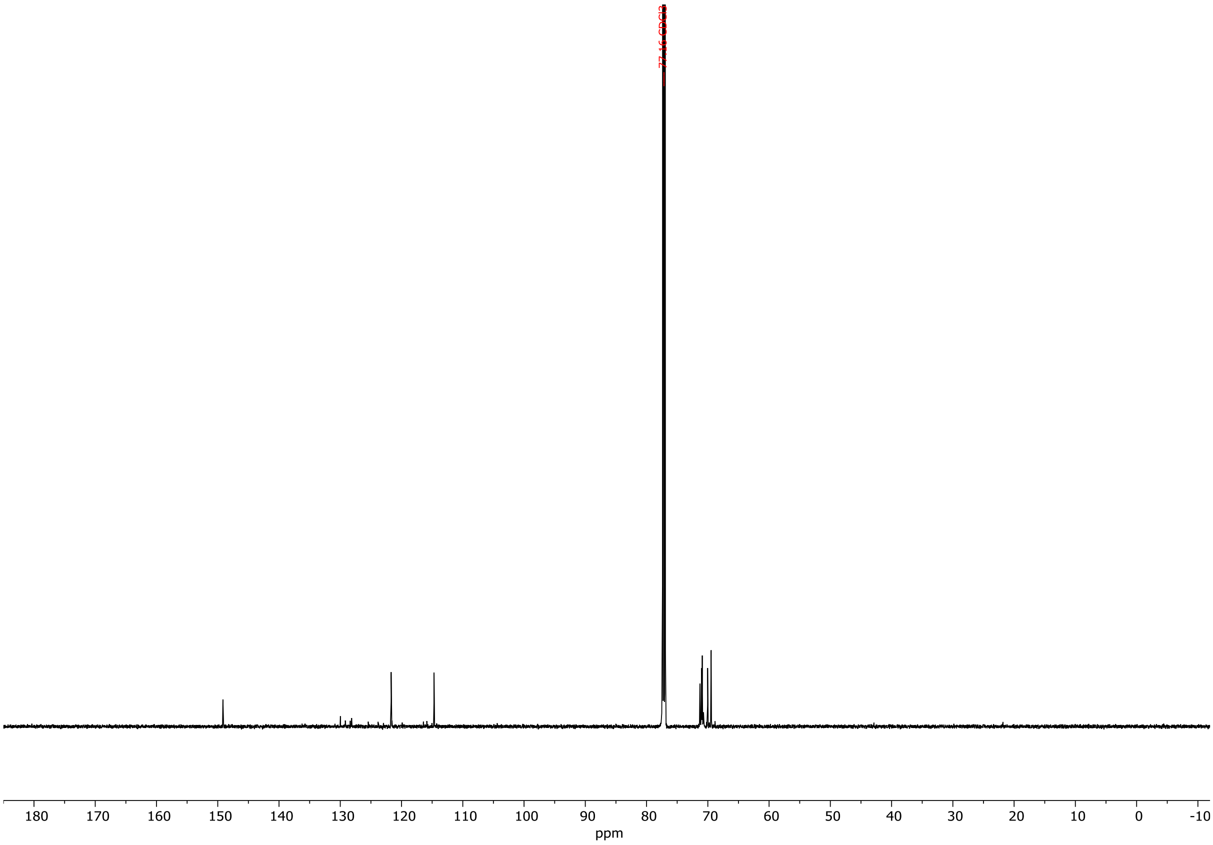


**Spectrum S24**: $^{13}C$ NMR ($CDCl_3$, 151 MHz) of **12**.

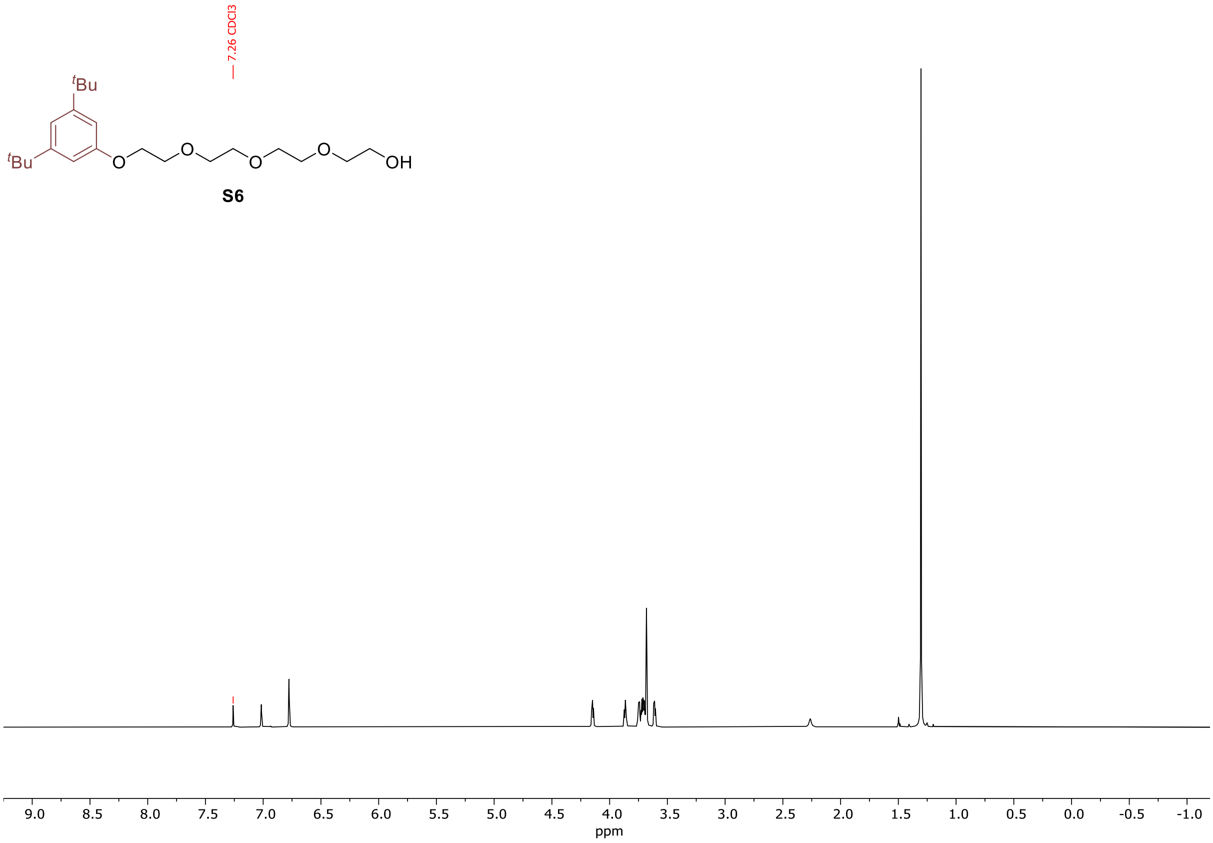


**Spectrum S25**: $^{1}H$ NMR ($CDCl_3$, 600 MHz) of **S6**.

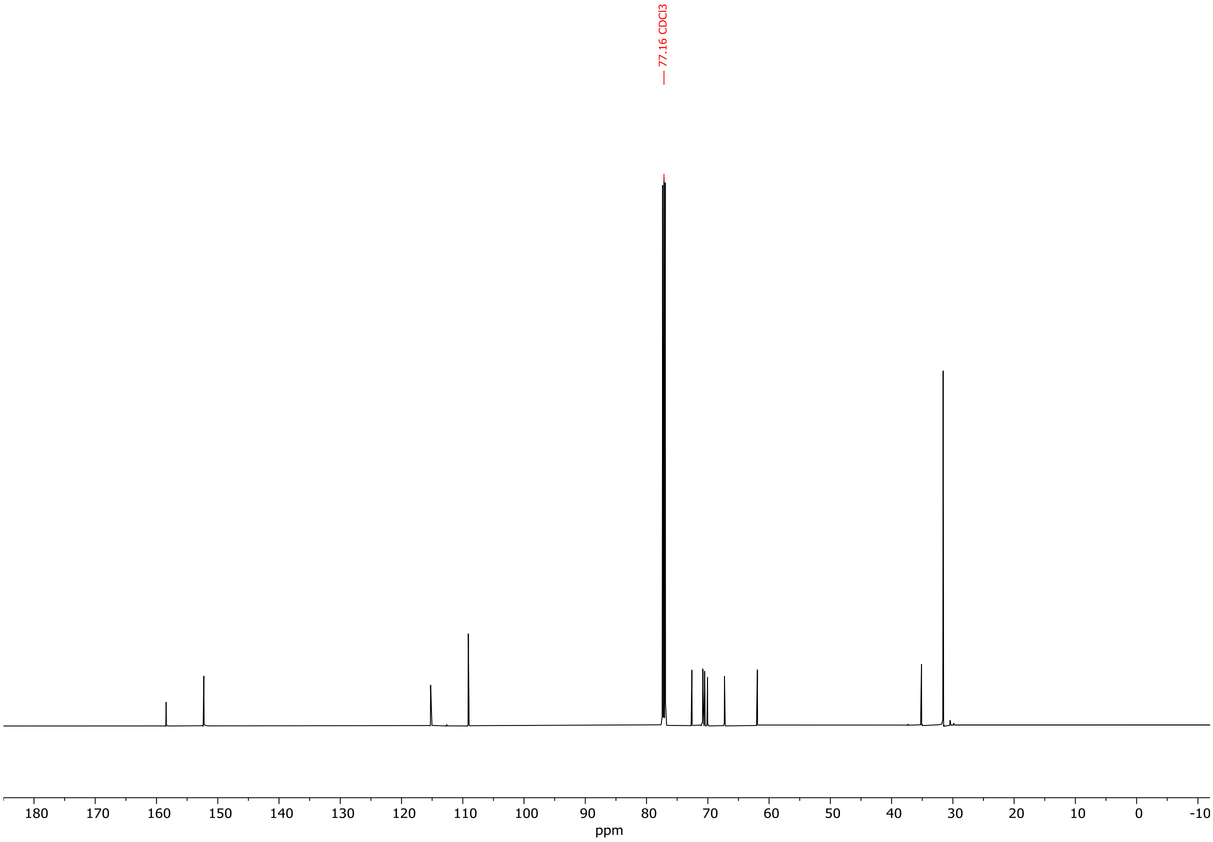


**Spectrum S26**: $^{13}C$ NMR ($CDCl_3$, 151 MHz) of **S6**.

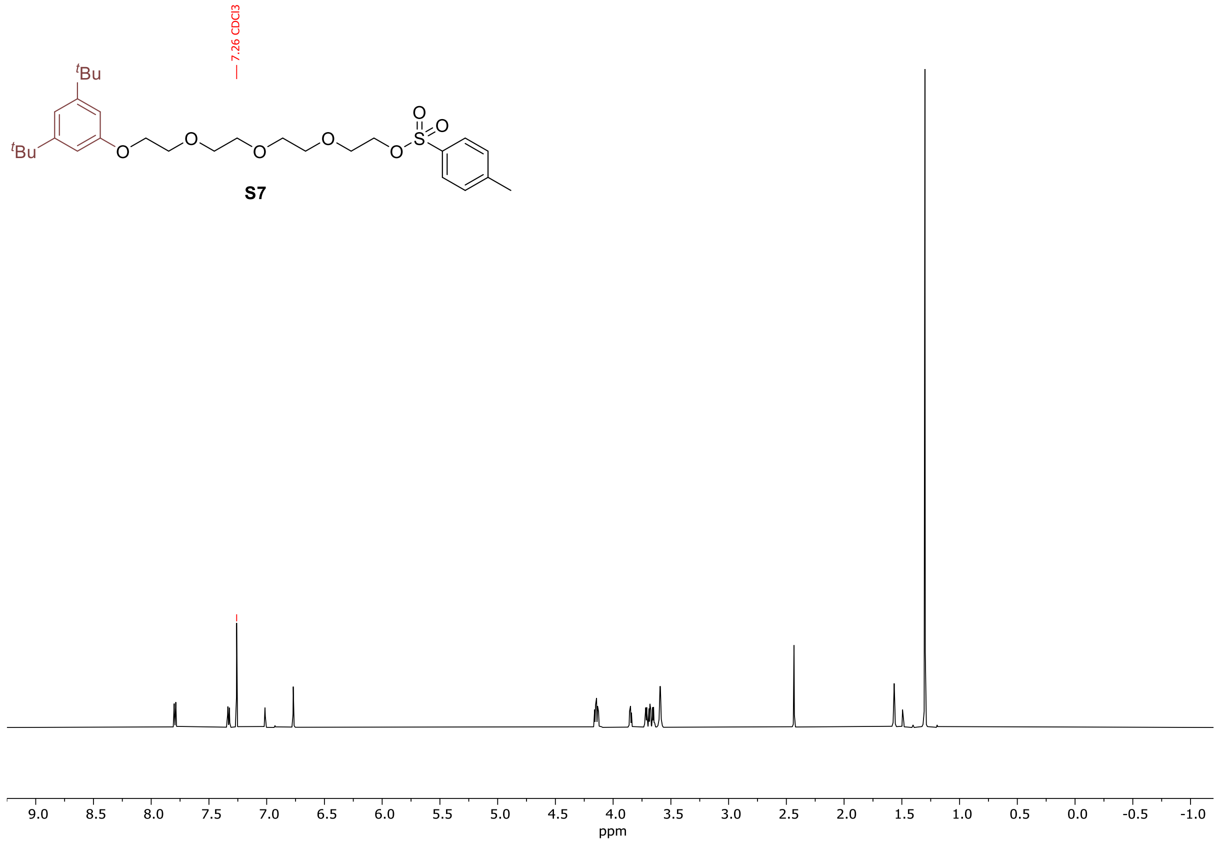


**Spectrum S27**: $^{1}$H NMR ($CDCl_3$, 600 MHz) of **S7**.

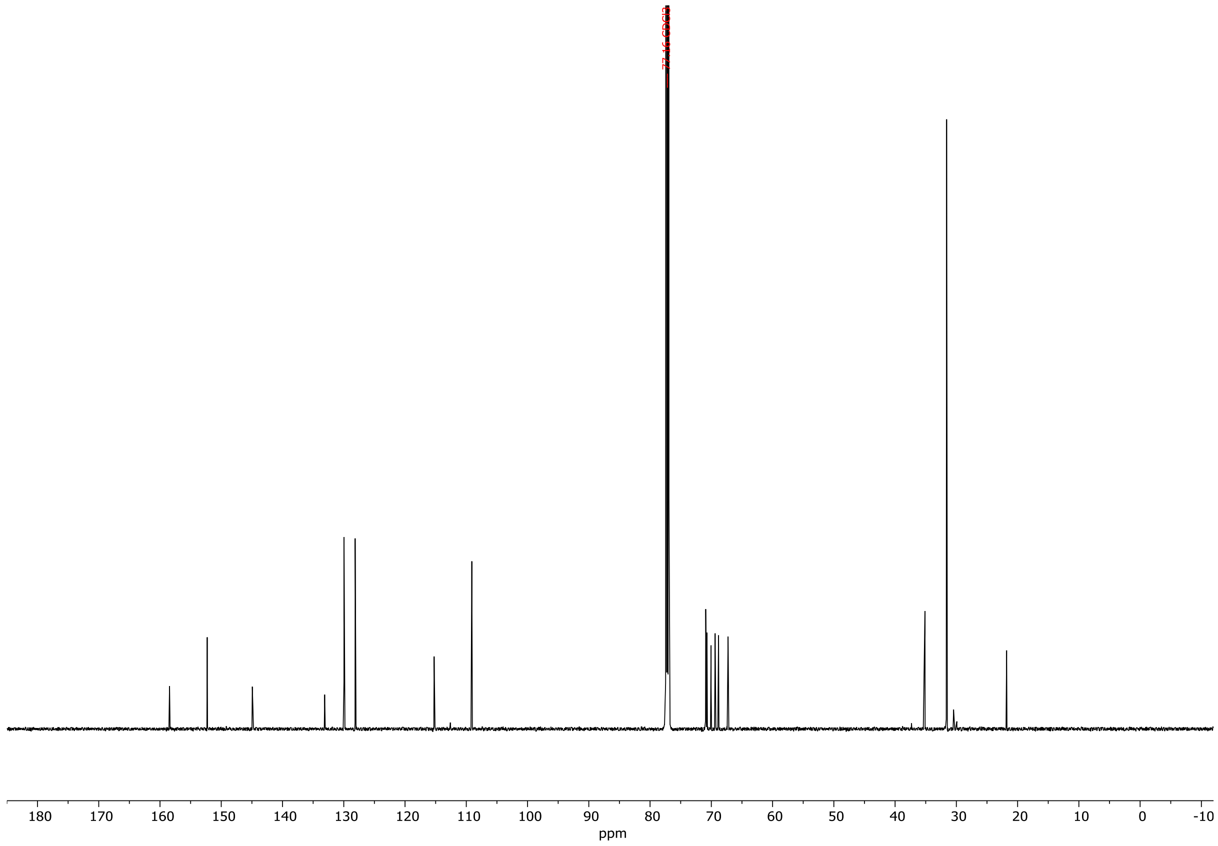


**Spectrum S28**: $^{13}$C NMR ($CDCl_3$, 151 MHz) of **S7**.

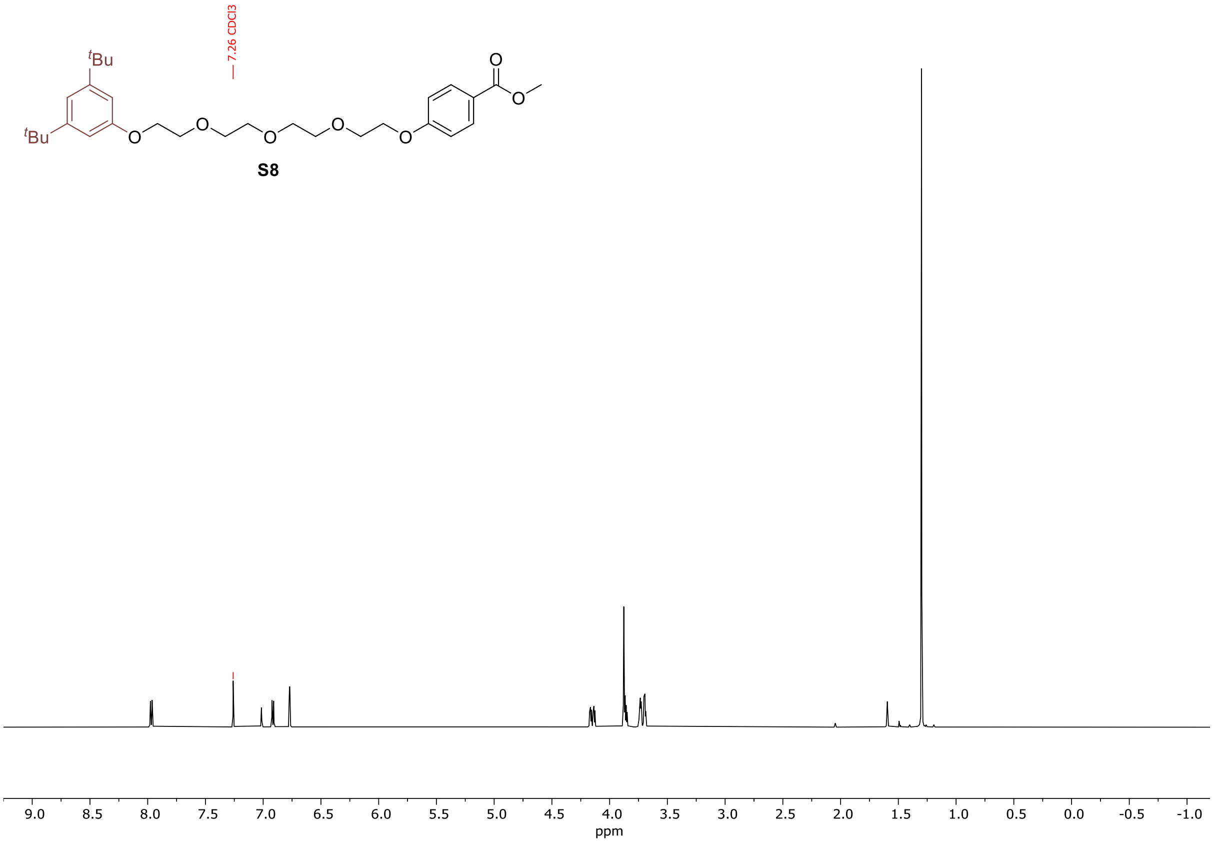


**Spectrum S29**: $^{1}H$ NMR ($CDCl_3$, 600 MHz) of **S8**.

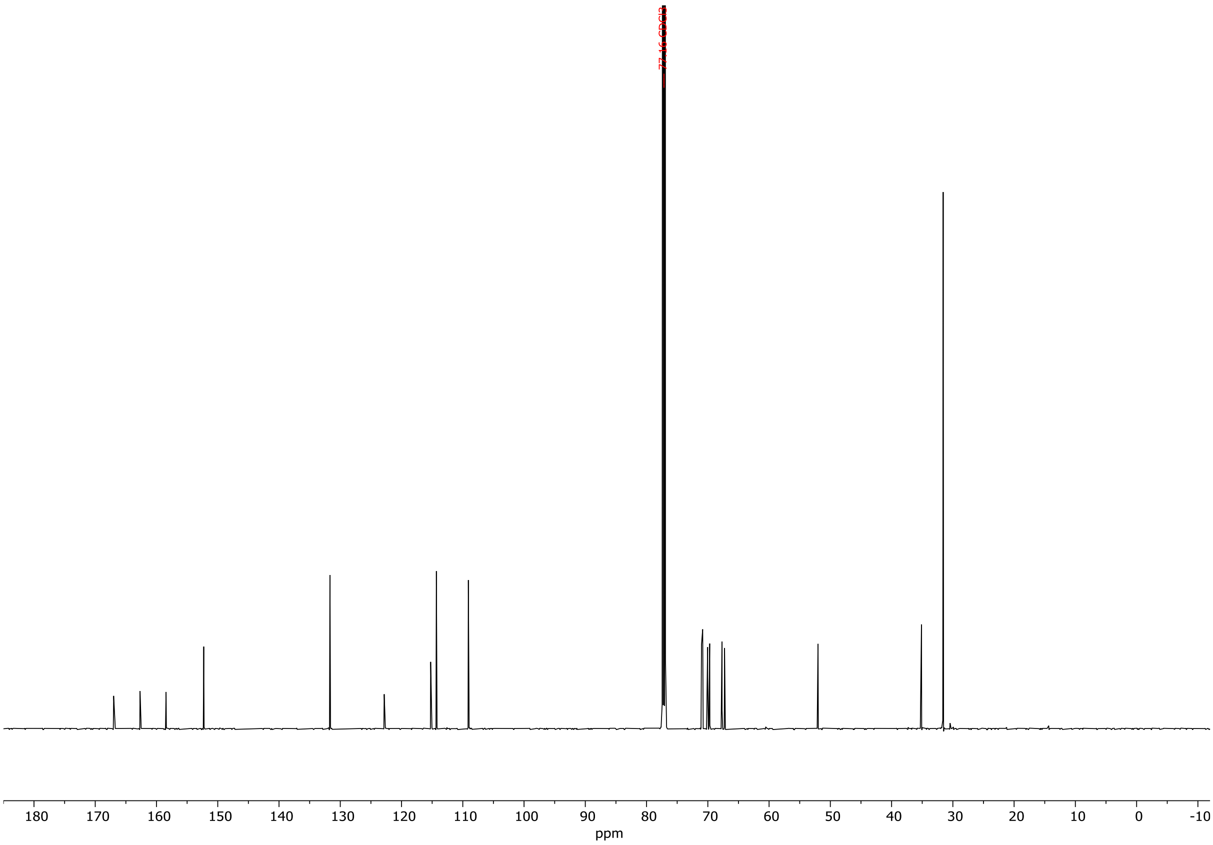


**Spectrum S30**: $^{13}C$ NMR ($CDCl_3$, 151 MHz) of **S8.**

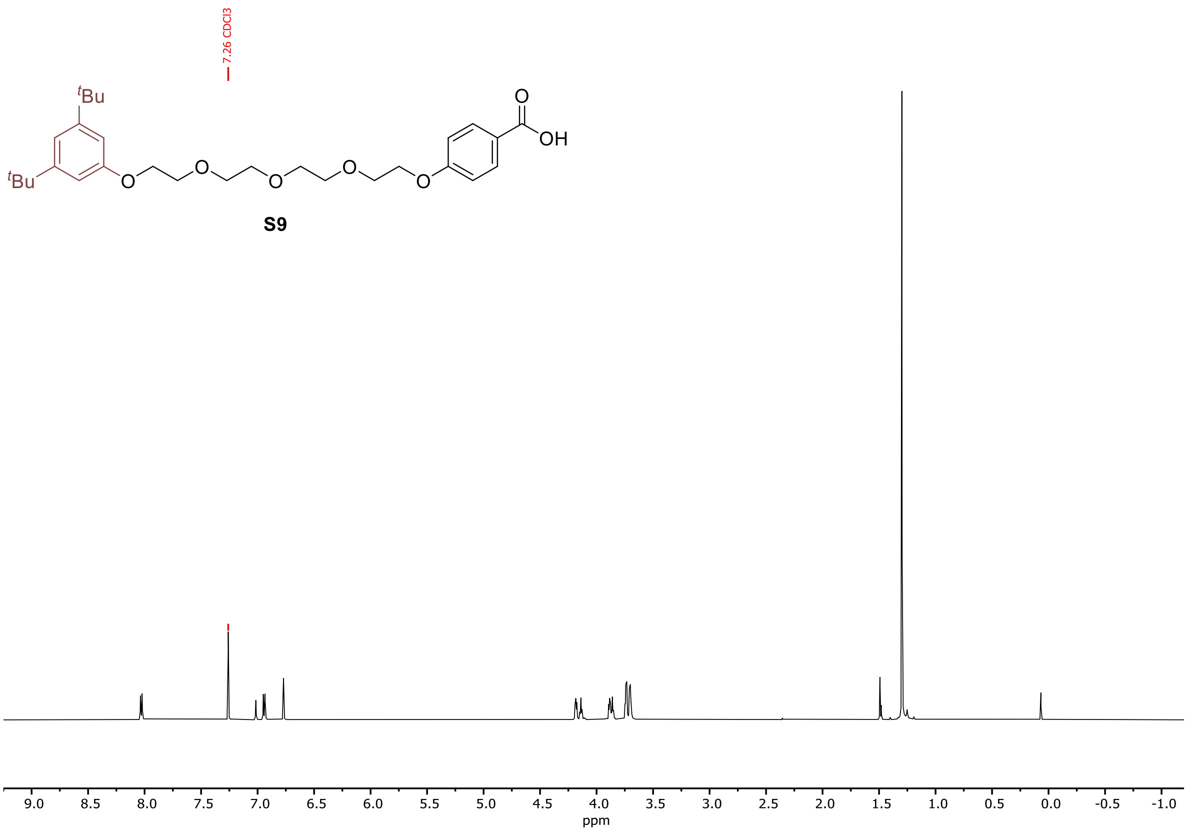


**Spectrum S31**: $^{1}H$ NMR ($CDCl_3$, 600 MHz) of **S9**.

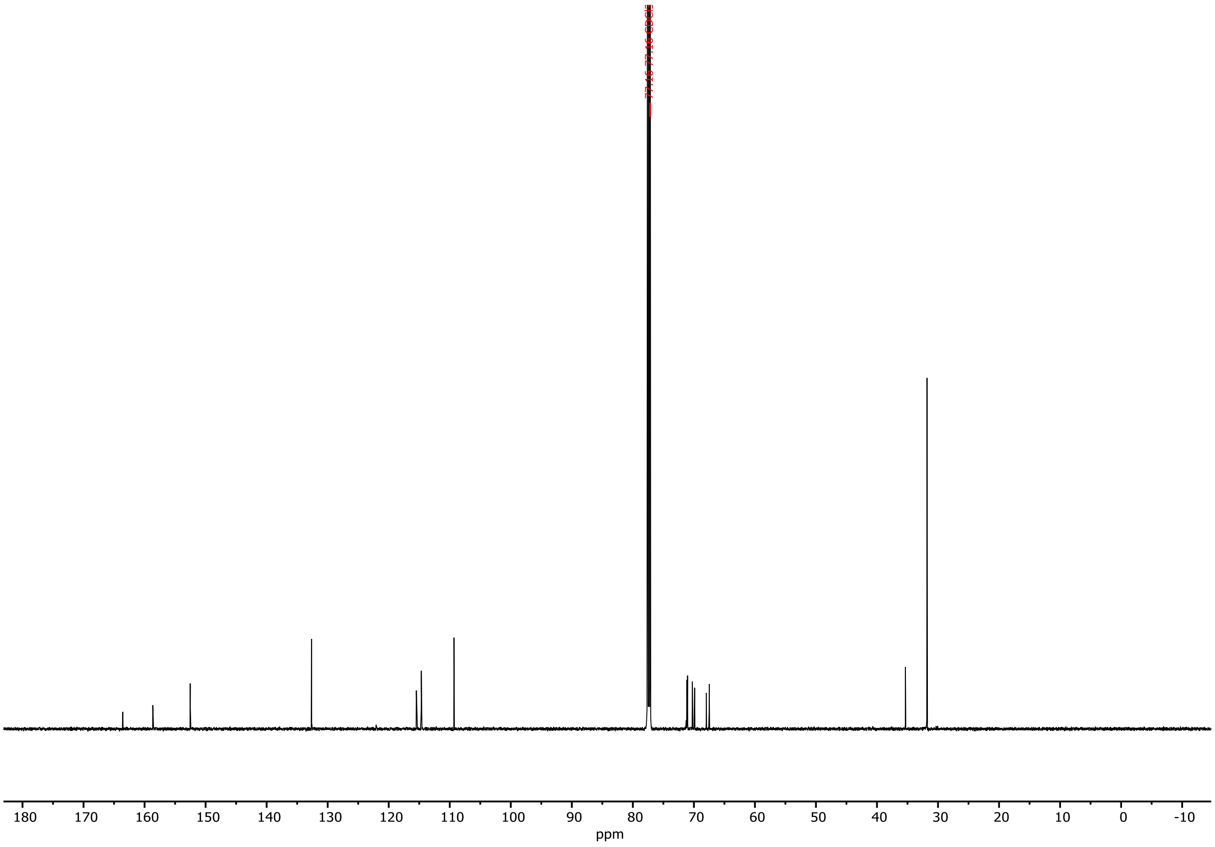


**Spectrum S32**: $^{13}C$ NMR ($CDCl_3$, 151 MHz) of **S9**.

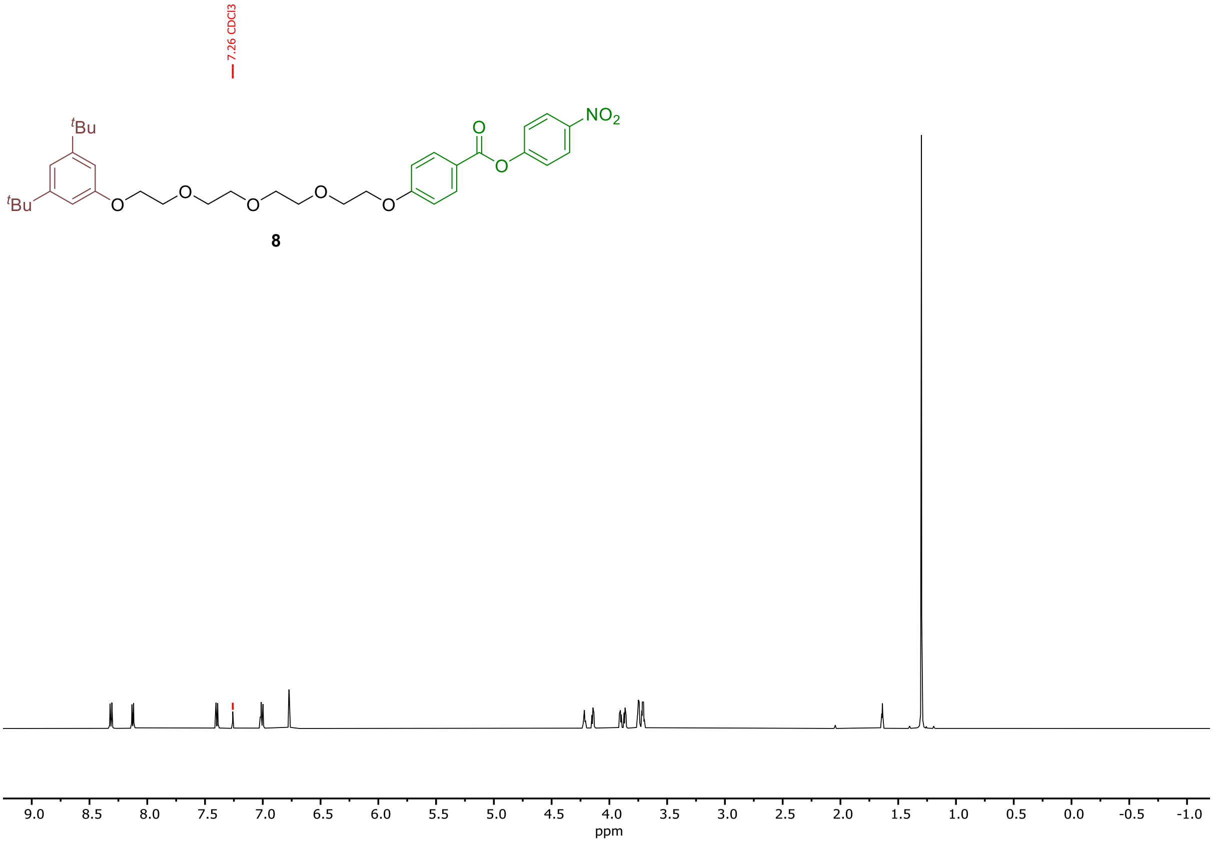


**Spectrum S33**: $^{1}H$ NMR ($CDCl_3$, 600 MHz) of **8**.

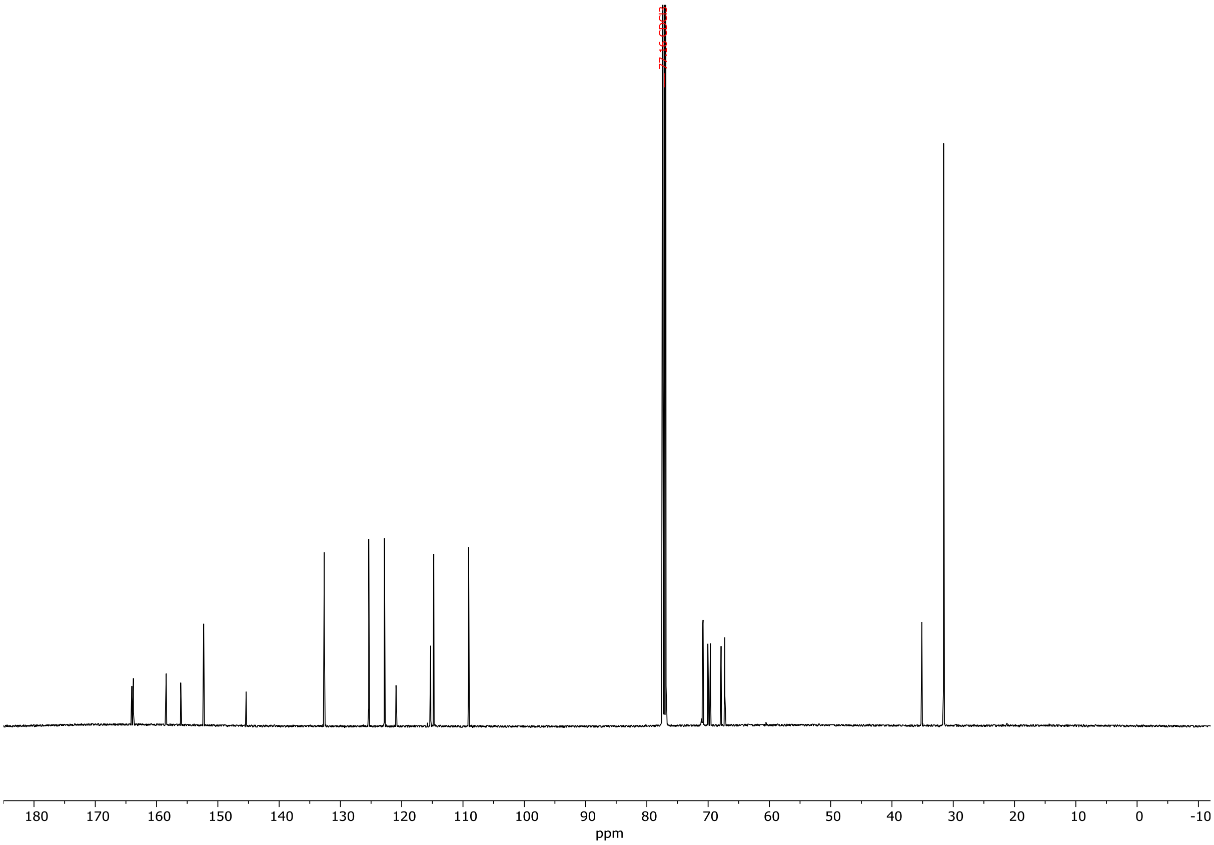


**Spectrum S34**: $^{13}C$ NMR ($CDCl_3$, 126 MHz) of **8**.

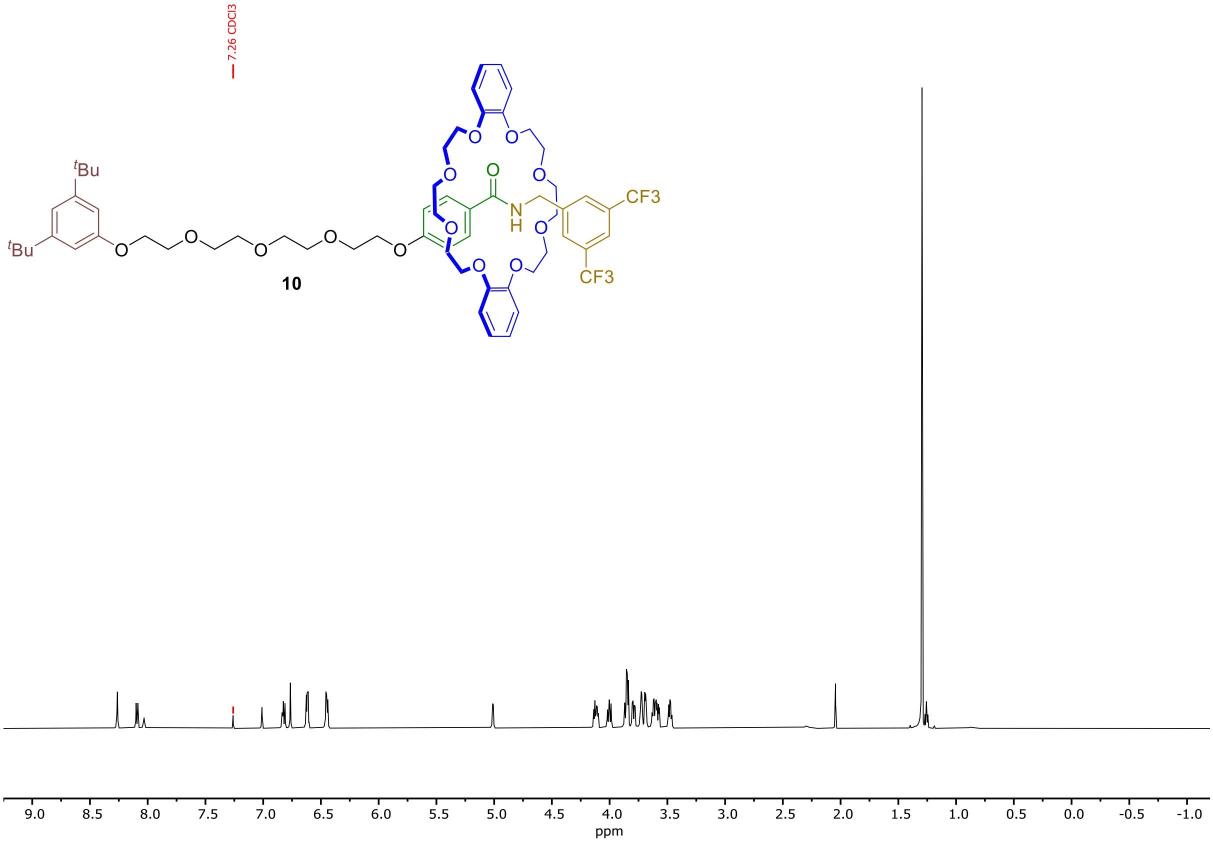


**Spectrum S35**: $^{1}H$ NMR ($CDCl_3$, 600 MHz) of **10**.

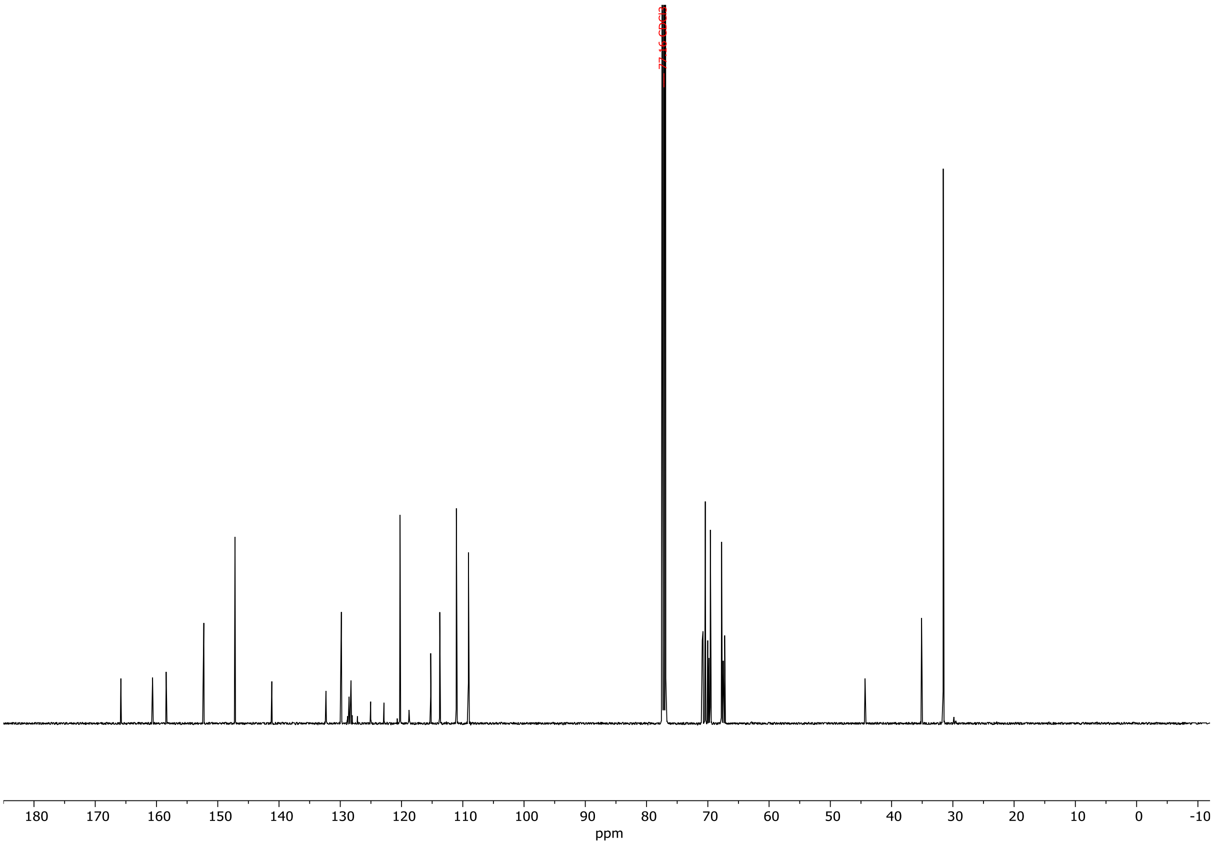


**Spectrum S36**: $^{13}C$ NMR ($CDCl_3$, 126 MHz) of **10**.

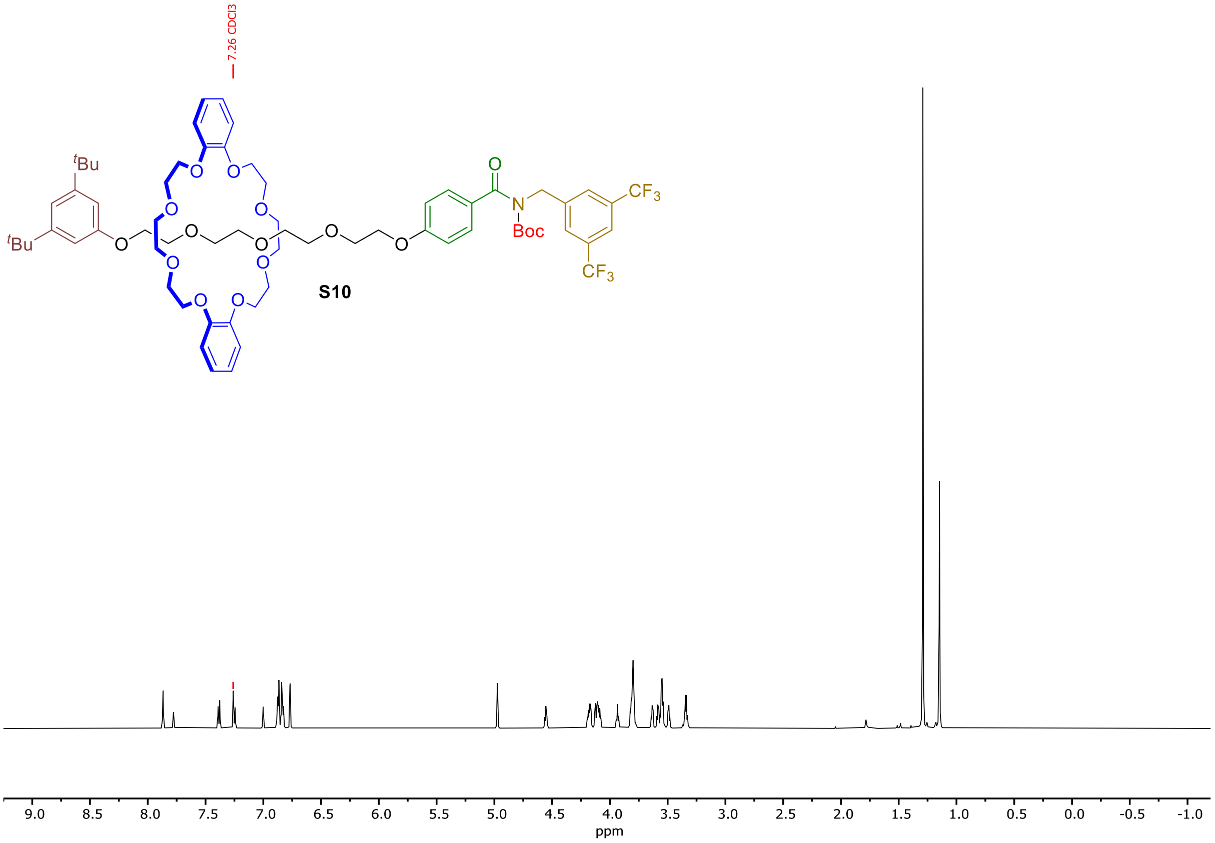


**Spectrum S37**: $^{1}H$ NMR ($CDCl_3$, 600 MHz) of **S10**.

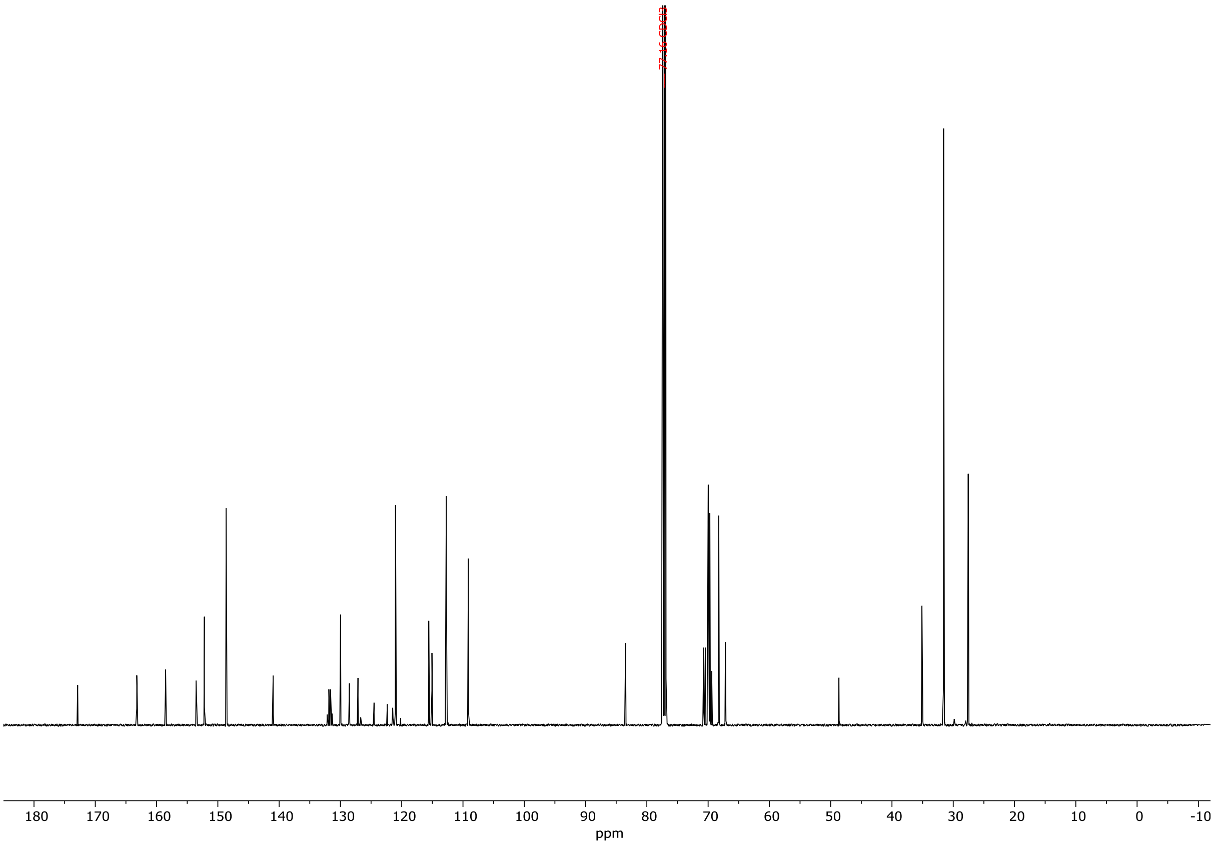


**Spectrum S38**: $^{13}C$ NMR ($CDCl_3$, 126 MHz) of **S10.**

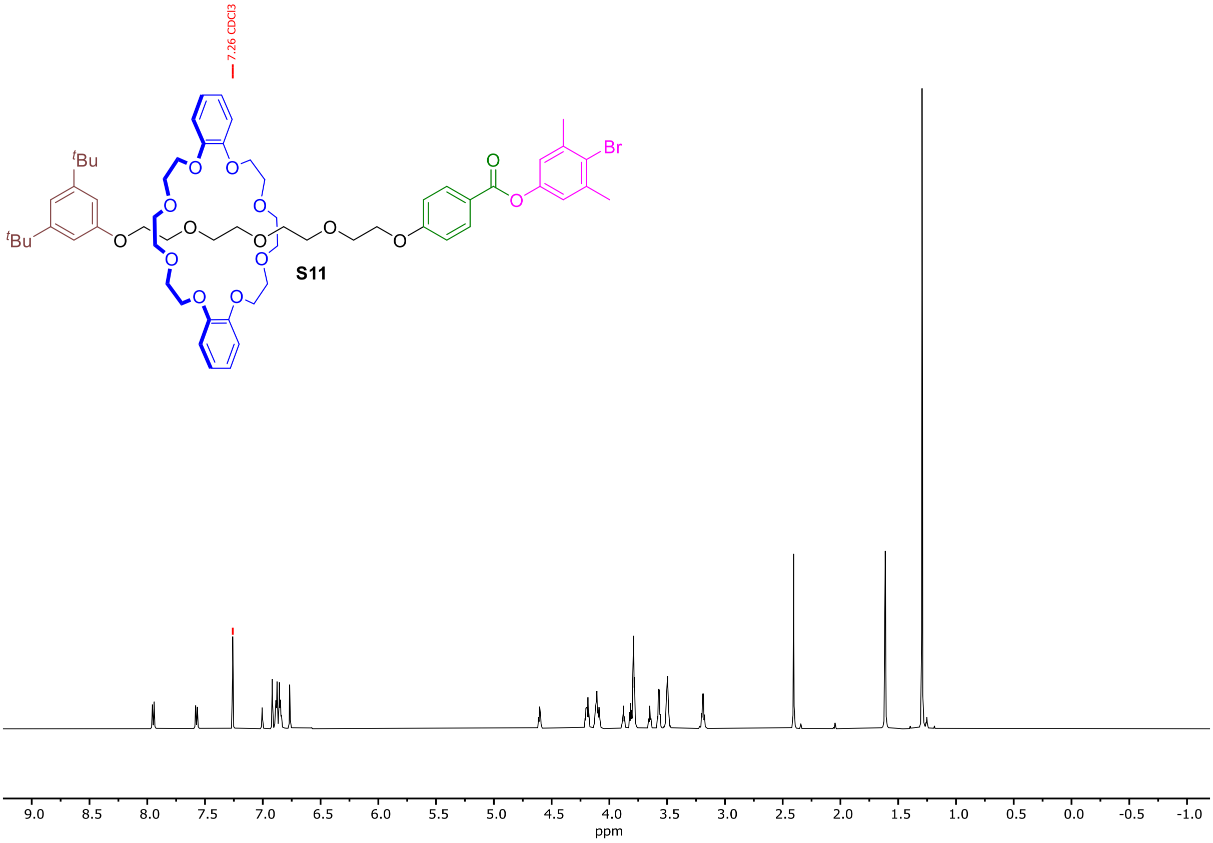


**Spectrum S39**: $^{1}H$ NMR ($CDCl_3$, 600 MHz) of **S11**.

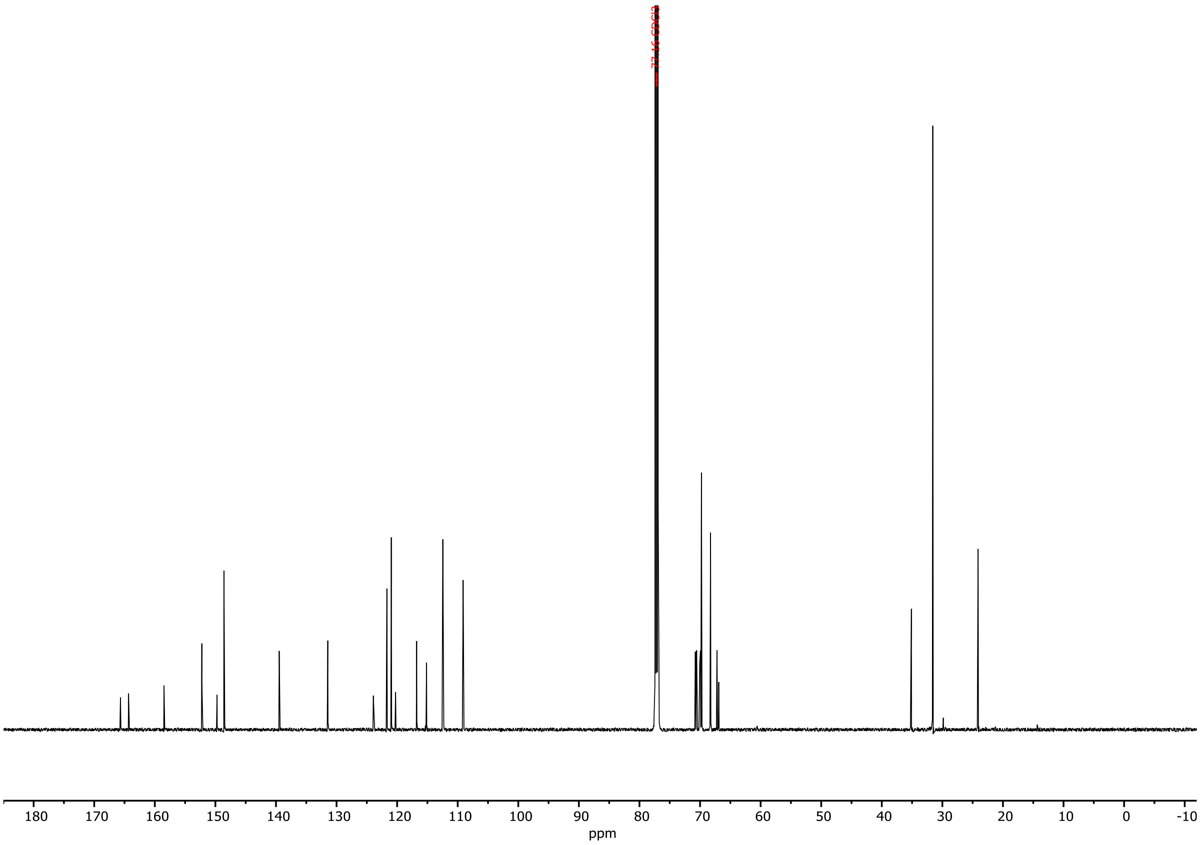


**Spectrum S40**: $^{13}C$ NMR ($CDCl_3$, 151 MHz) of **S11**.

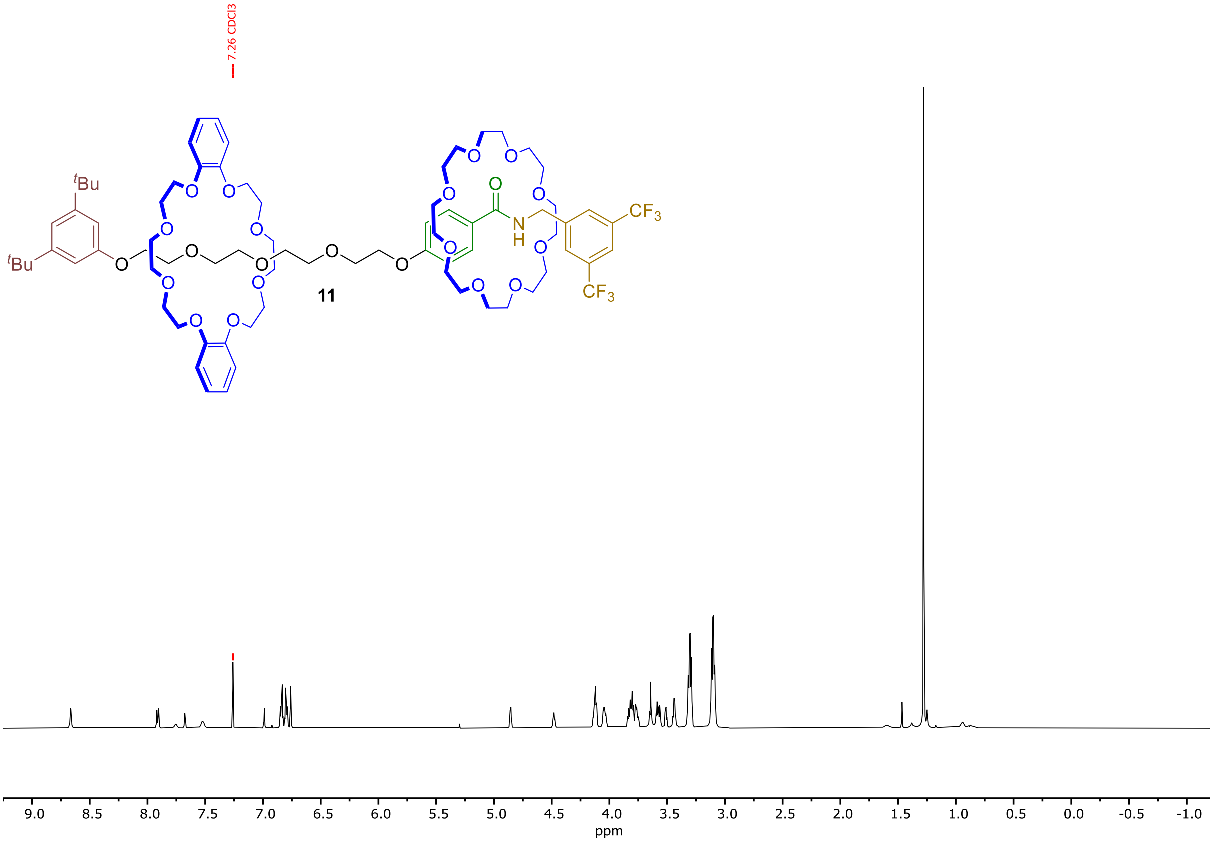


**Spectrum S41**: $^{1}H$ NMR ($CDCl_3$, 600 MHz) of **11**.

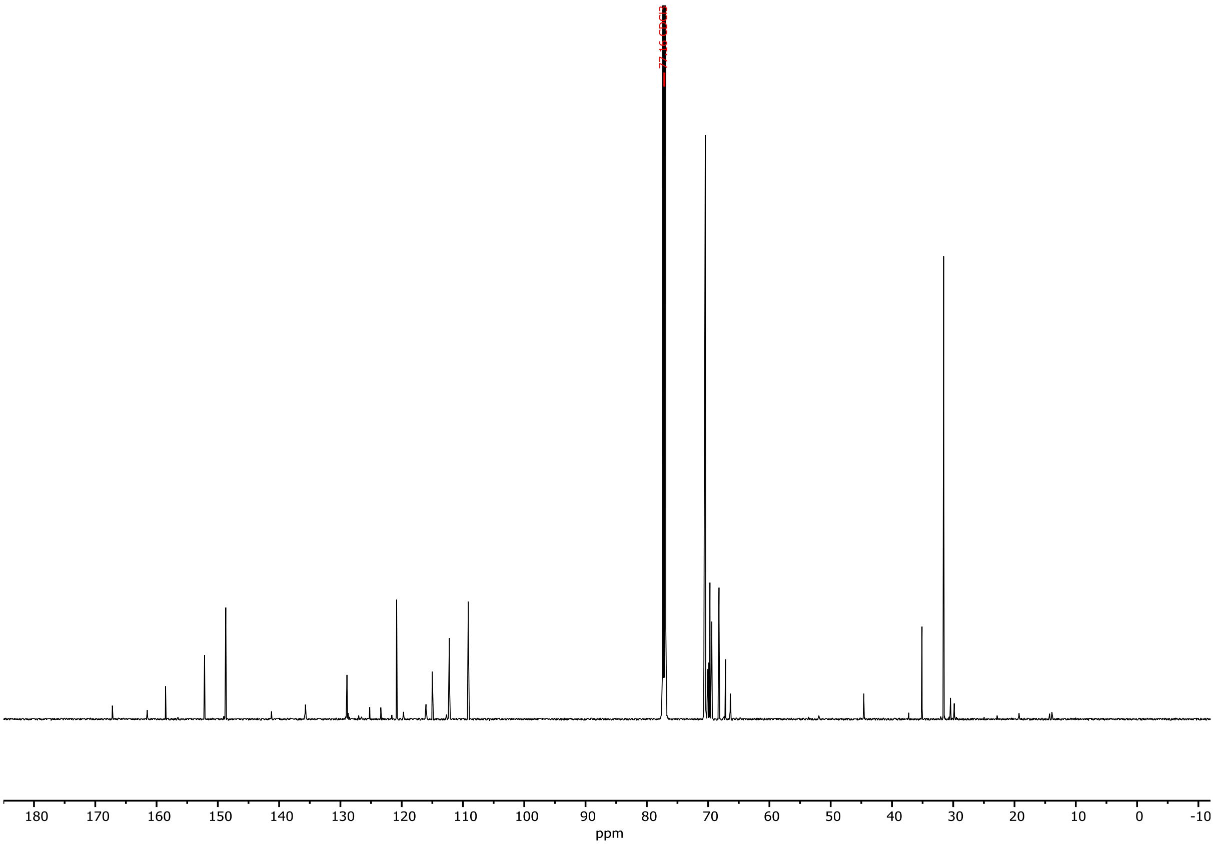


**Spectrum S42**: $^{13}C$ NMR ($CDCl_3$, 151 MHz) of **11**.

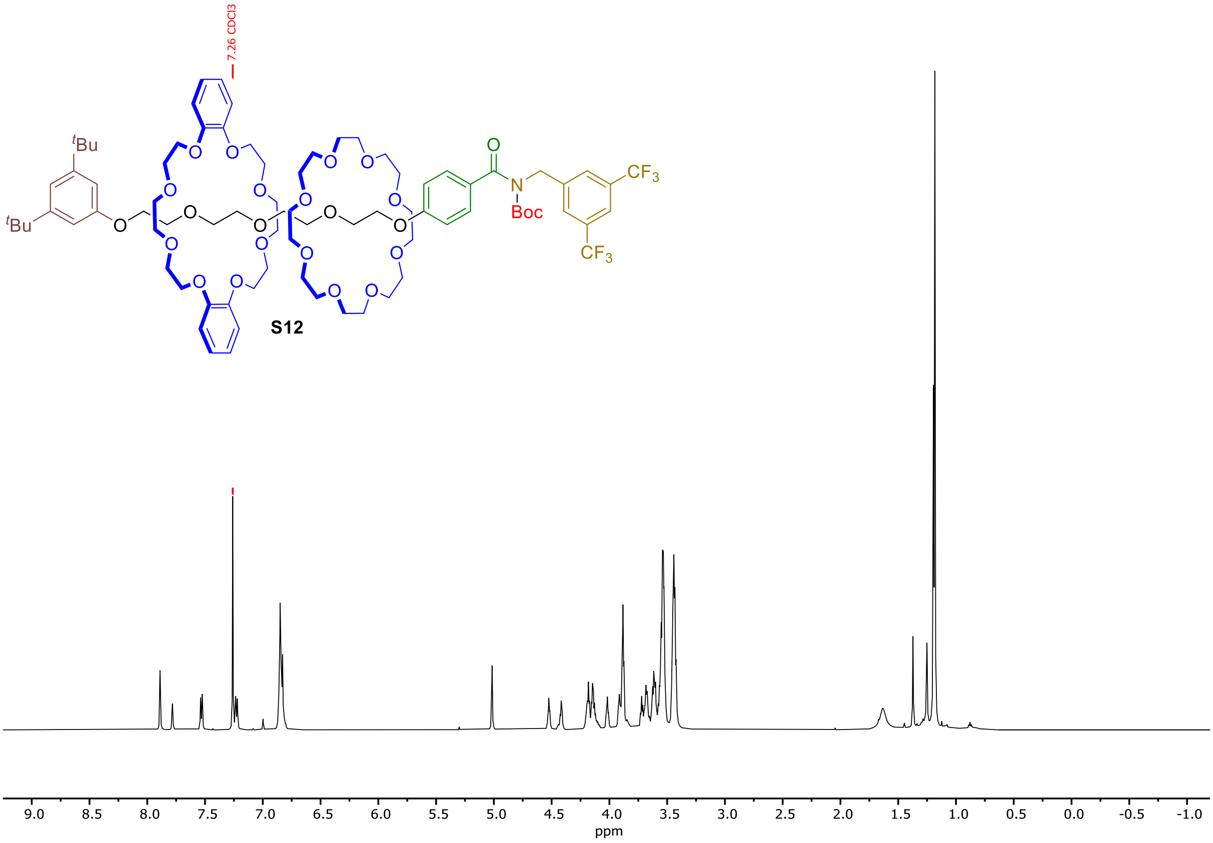


**Spectrum S43**: $^{1}H$ NMR ($CDCl_3$, 600 MHz) of **S12**.

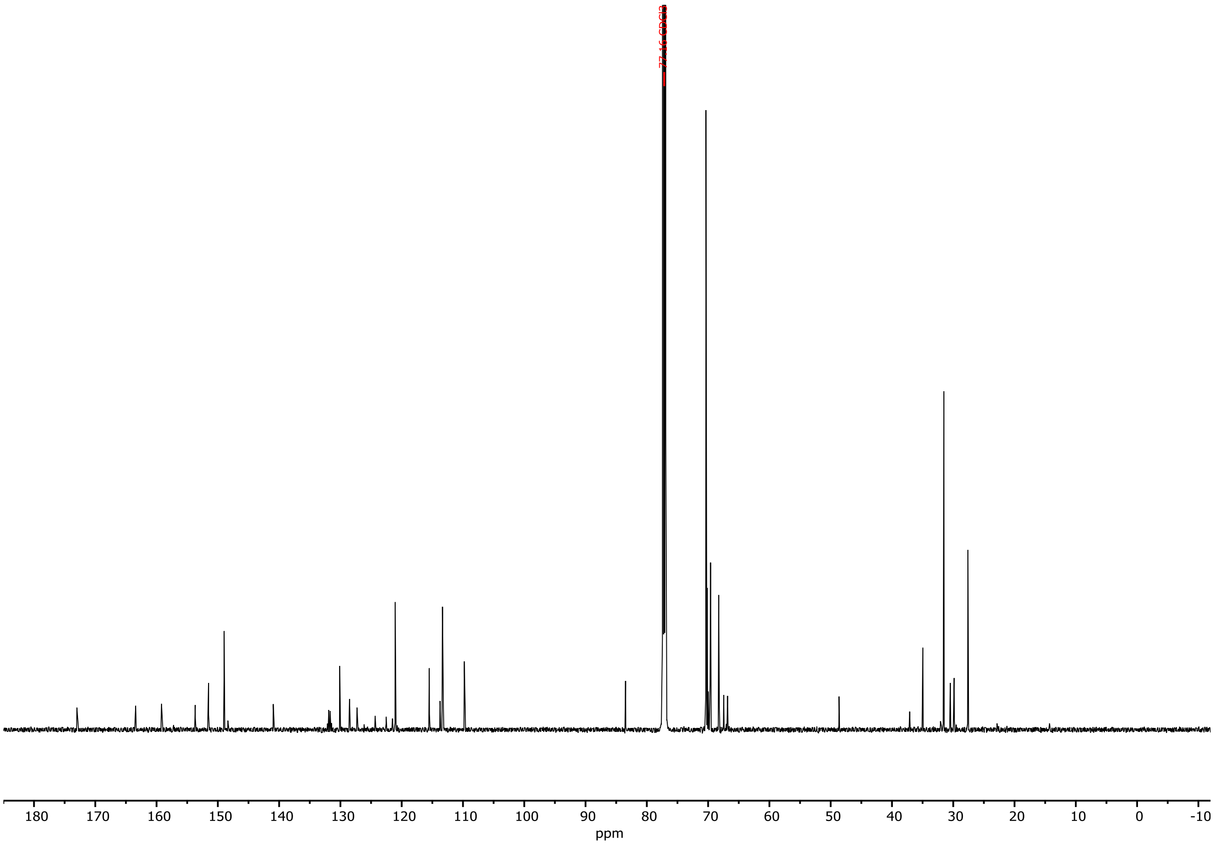


**Spectrum S44**: $^{13}C$ NMR ($CDCl_3$, 151 MHz) of **S12**.

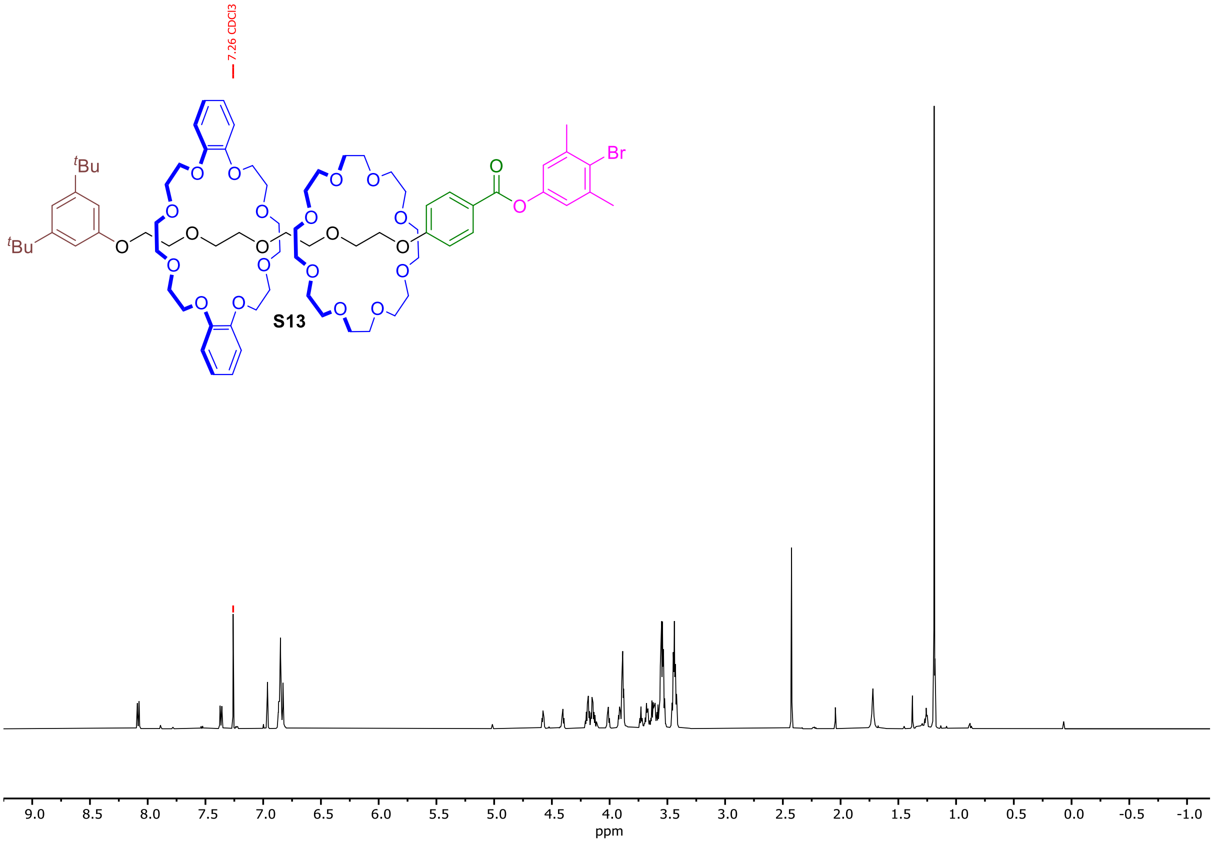


**Spectrum S45**: $^{1}H$ NMR ($CDCl_3$, 600 MHz) of **S13**.

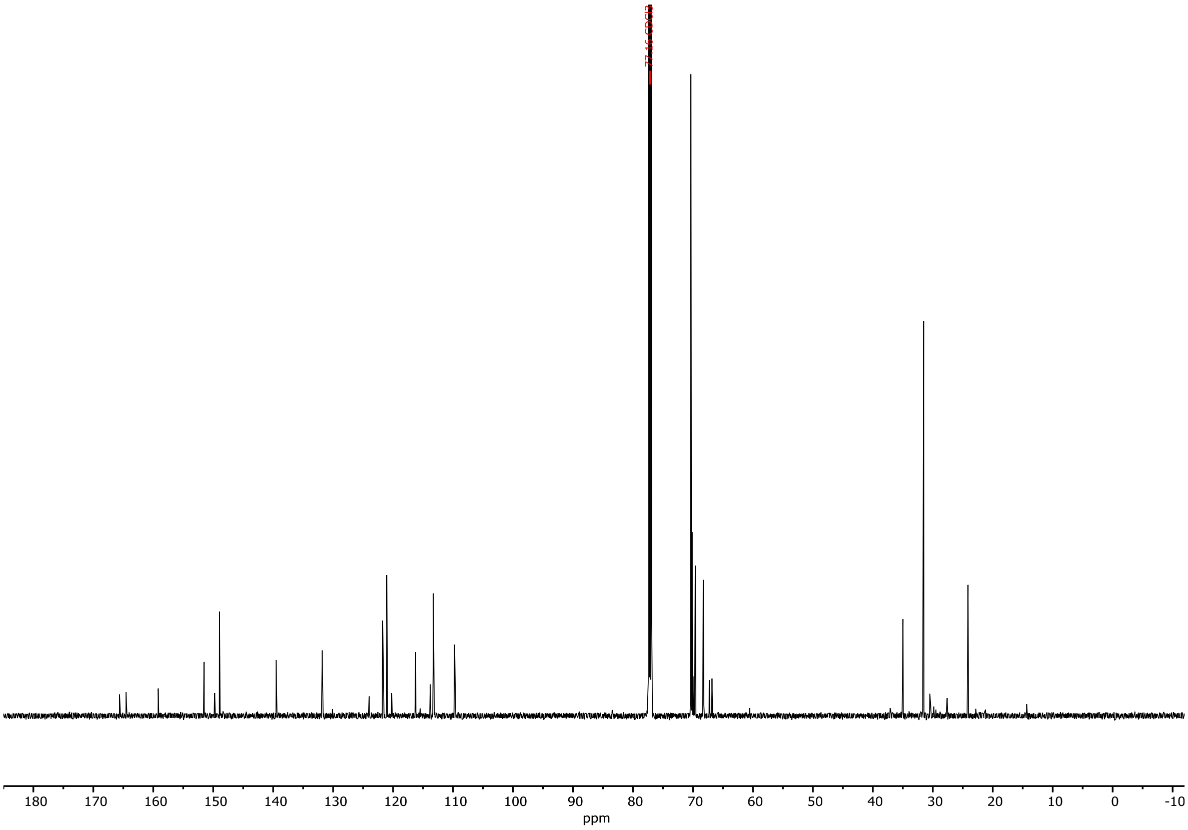


**Spectrum S46**: $^{13}C$ NMR ($CDCl_3$, 151 MHz) of **S13**.

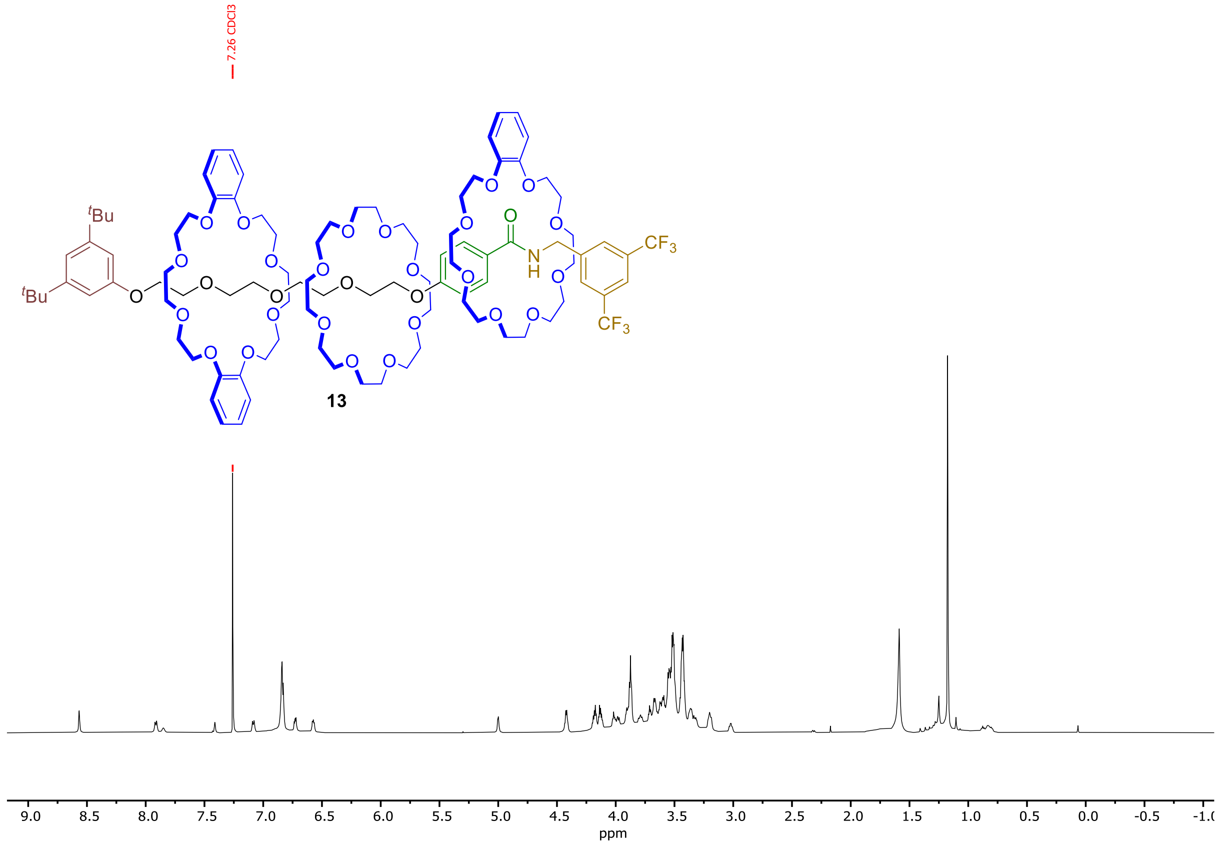


**Spectrum S47**: $^{1}H$ NMR ($CDCl_3$, 600 MHz) of **13**.

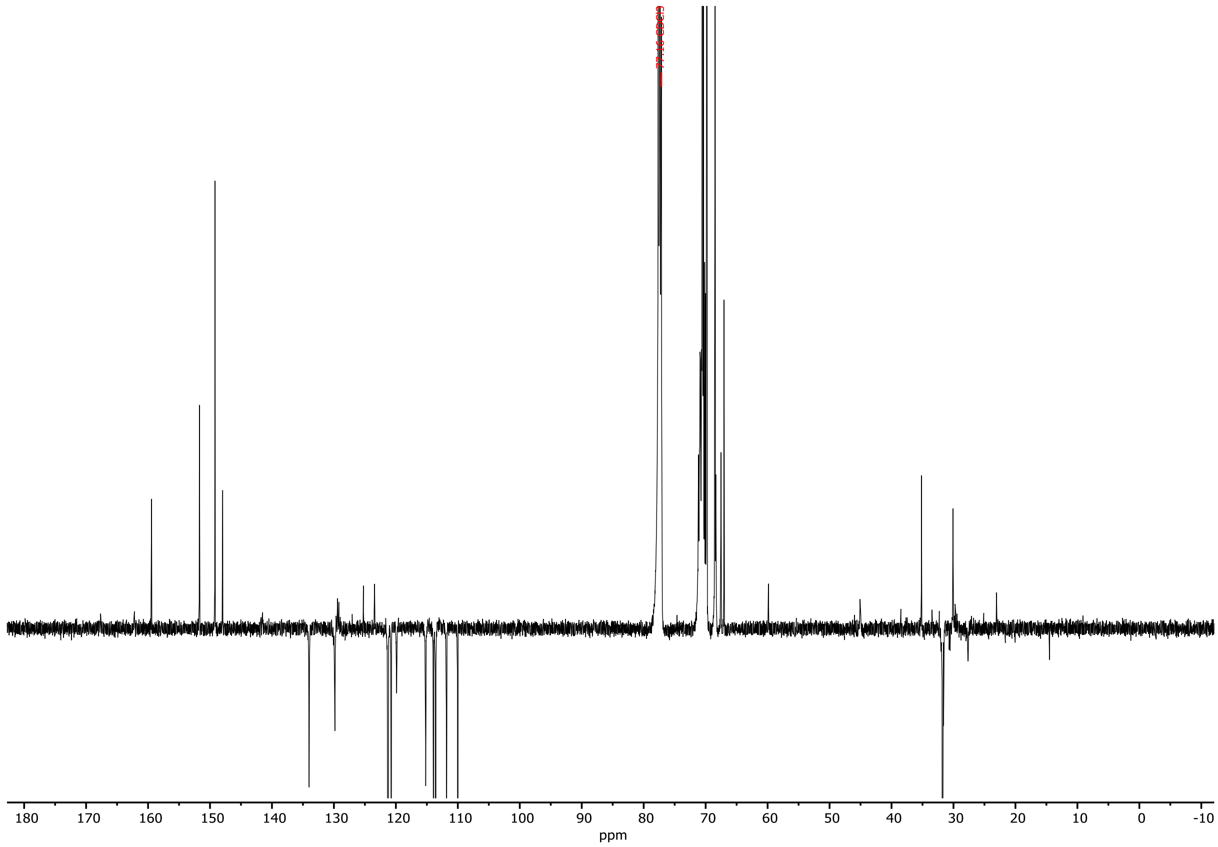


**Spectrum S48**: $^{13}C$ DEPTQ NMR ($CDCl_3$, 151 MHz) of **13.**

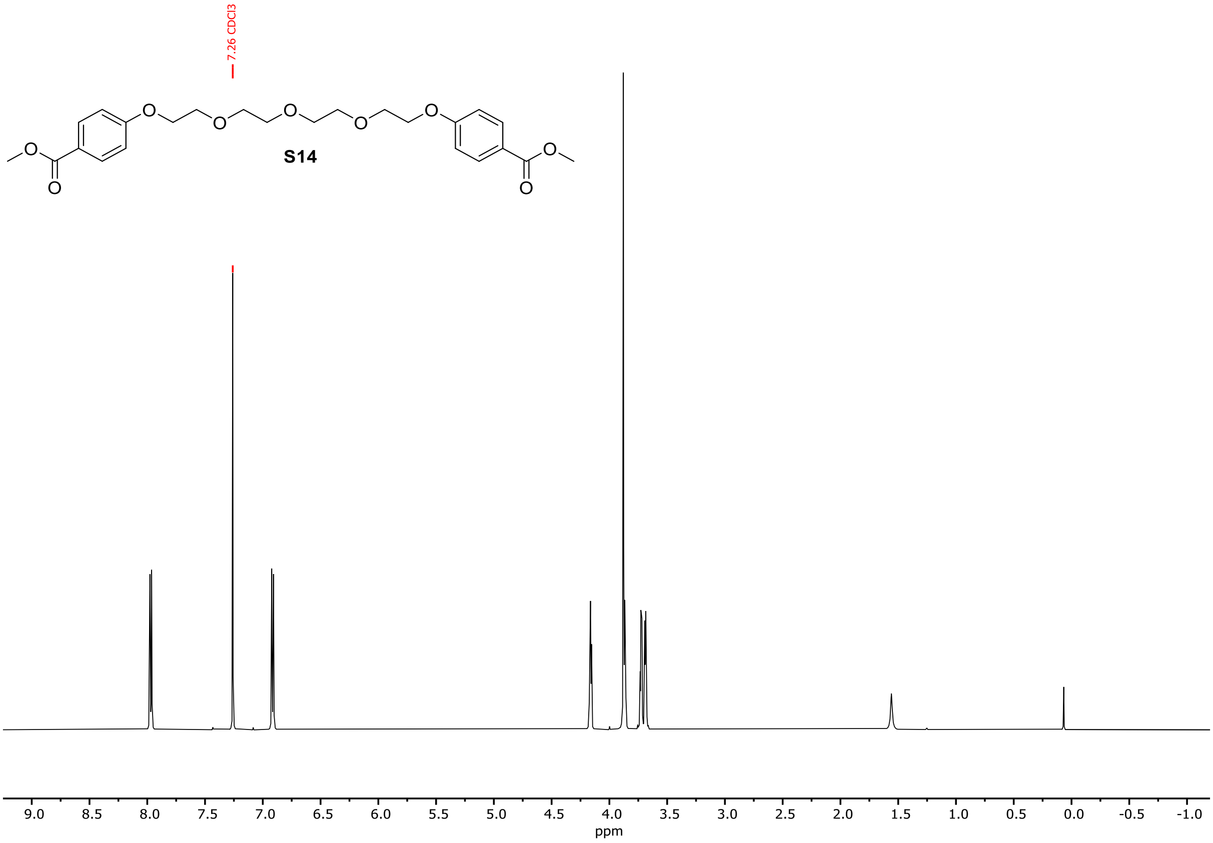


**Spectrum S49**: $^{1}H$ NMR ($CDCl_3$, 600 MHz) of **S14**.

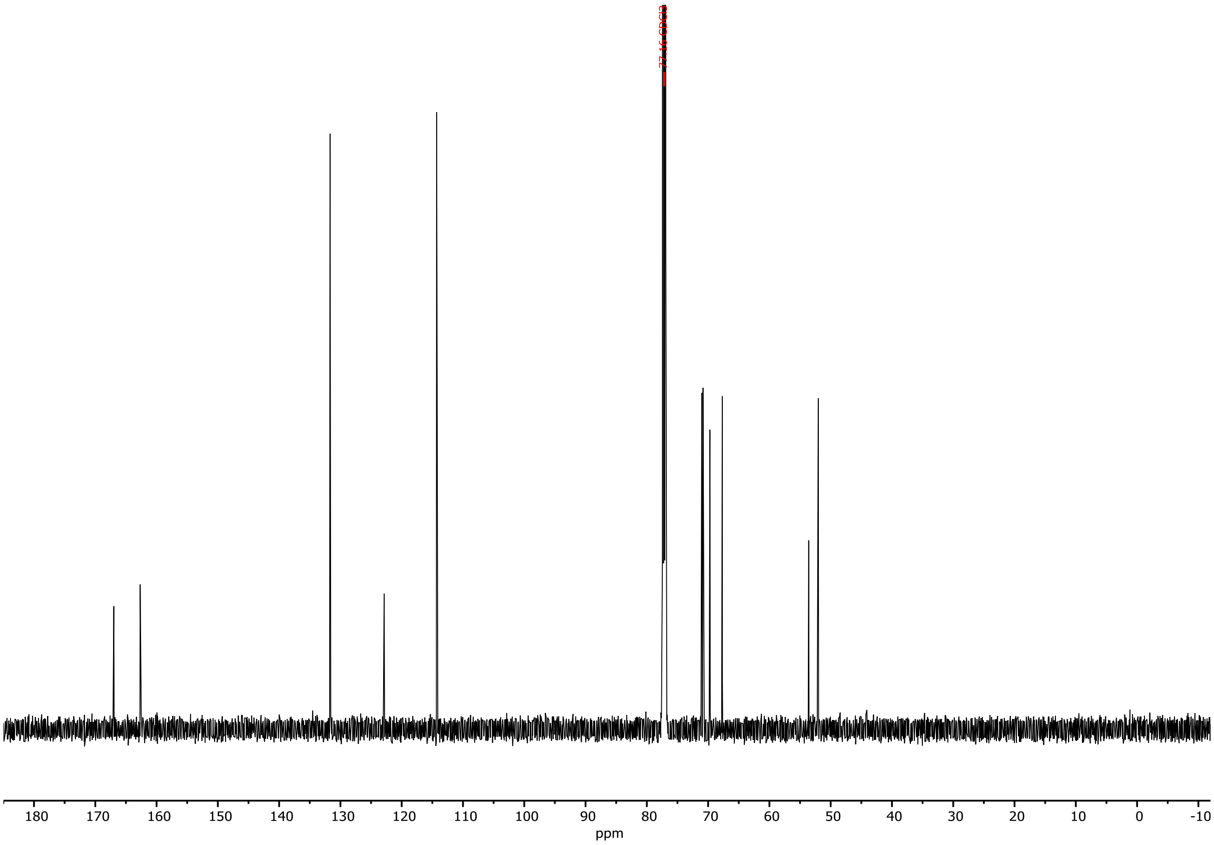


**Spectrum S50**: $^{13}C$ NMR ($CDCl_3$, 151 MHz) of **S14**.

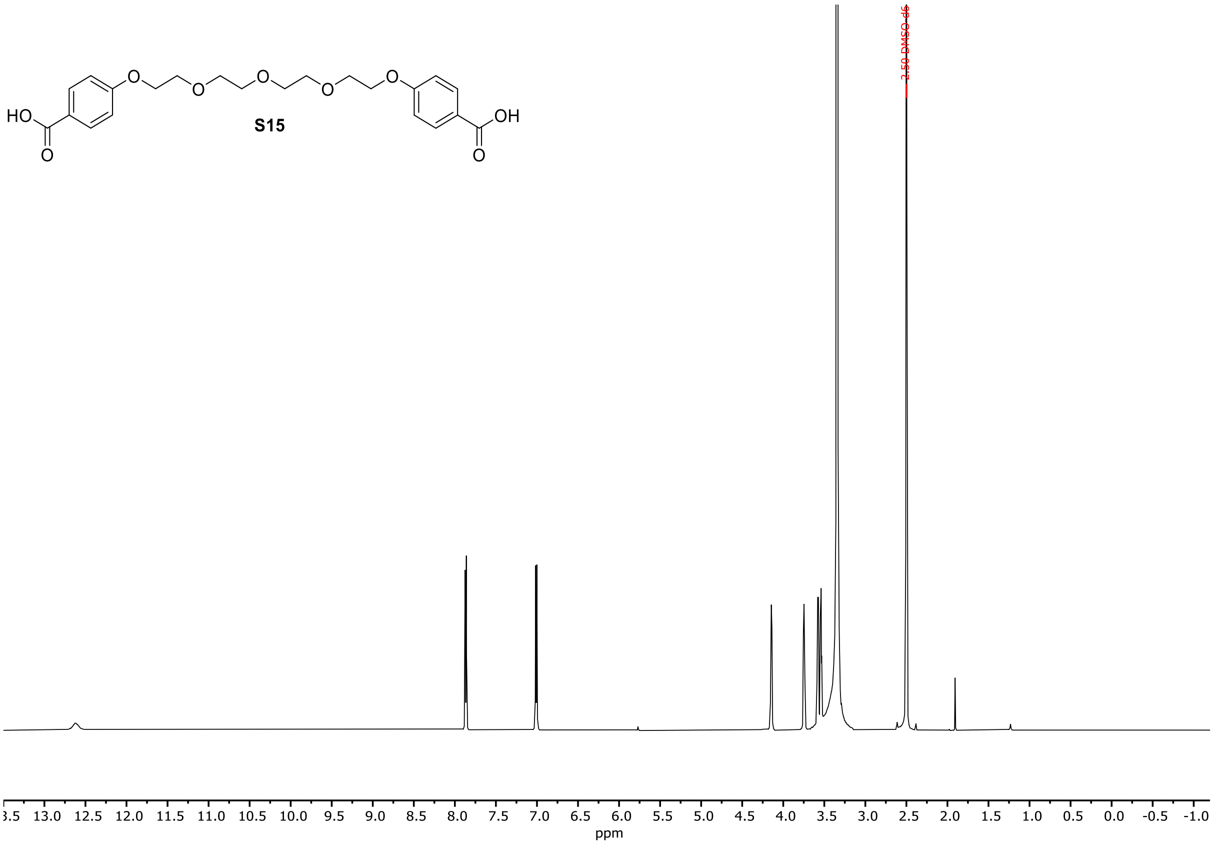


**Spectrum S51**: $^{1}H$ NMR ($(CD_3)_2SO$, 600 MHz) of **S15**.

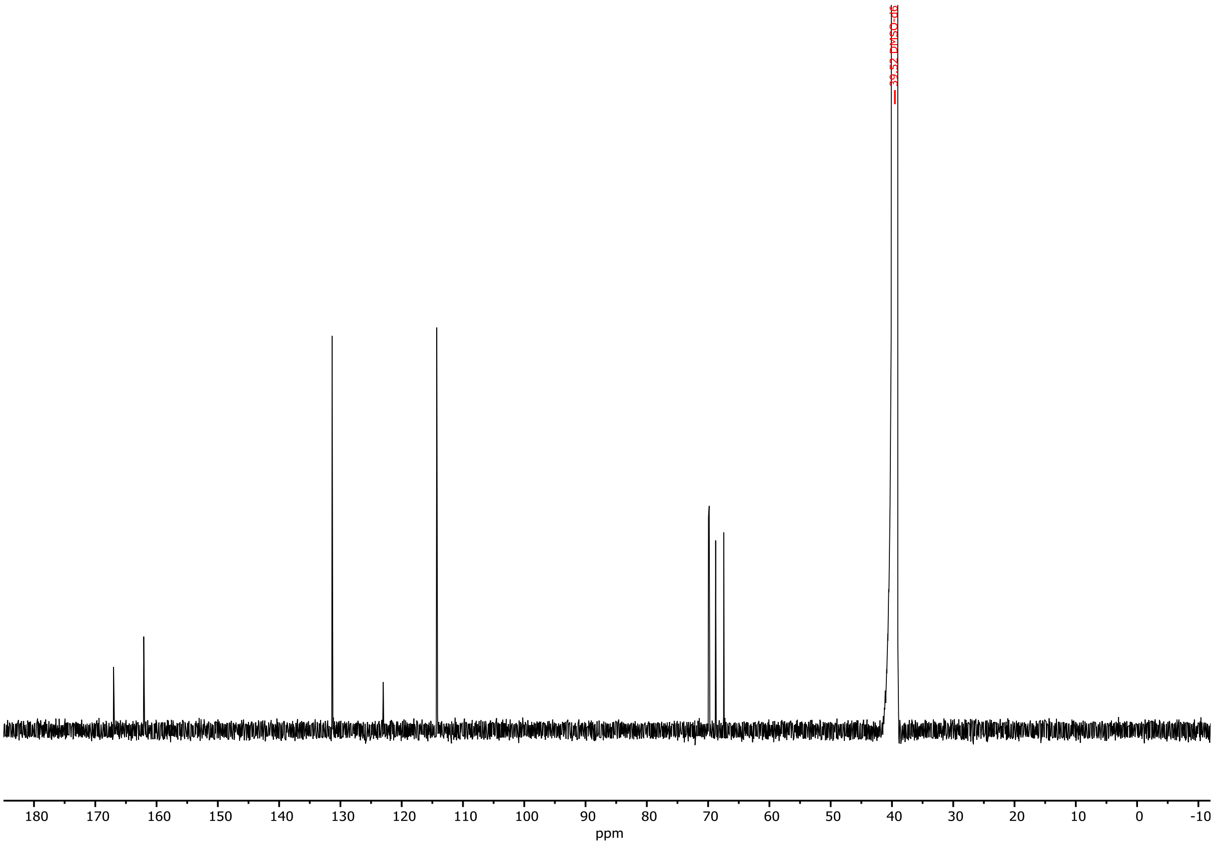


**Spectrum S52**: $^{13}C$ NMR ($(CD_3)_2SO$, 151 MHz) of **S15**.

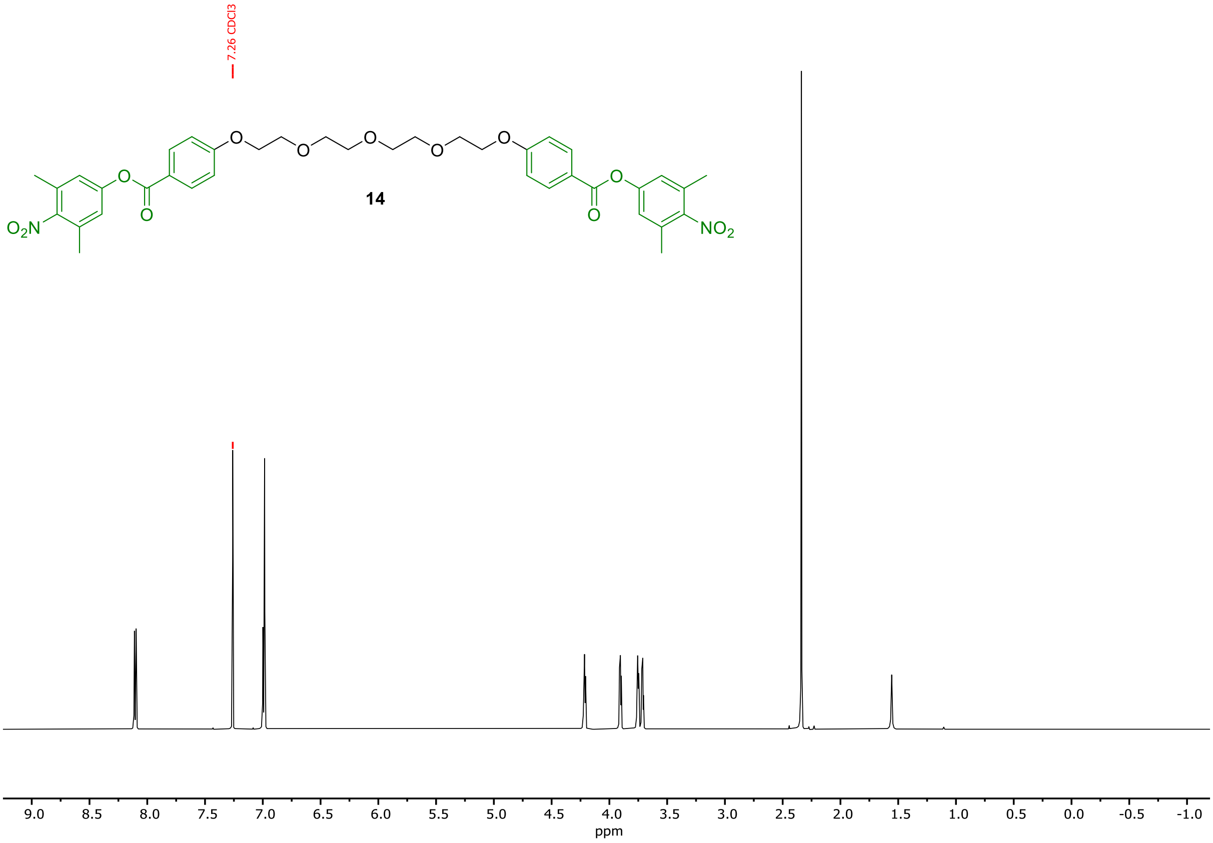


**Spectrum S53**: $^{1}H$ NMR ($CDCl_3$, 600 MHz) of **14**.

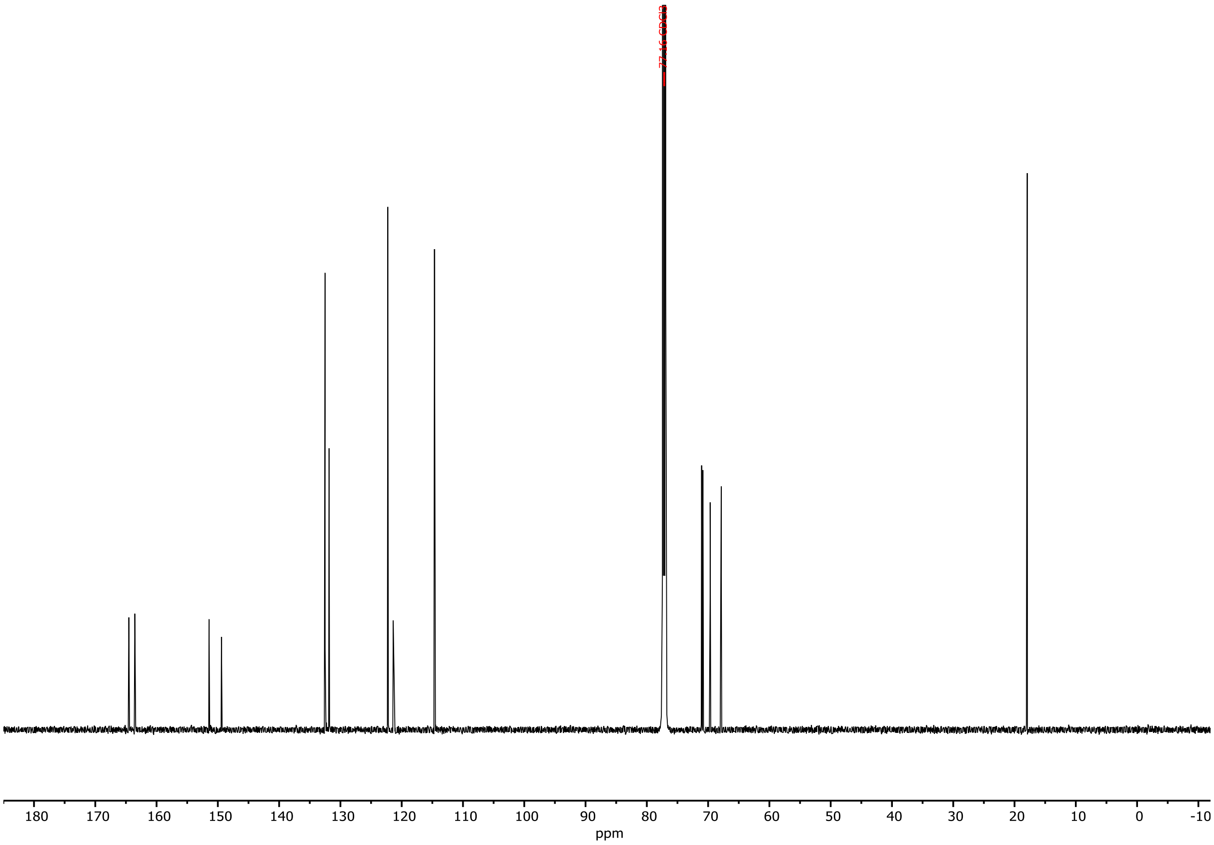


**Spectrum S54**: $^{13}C$ NMR ($CDCl_3$, 151 MHz) of **14**.

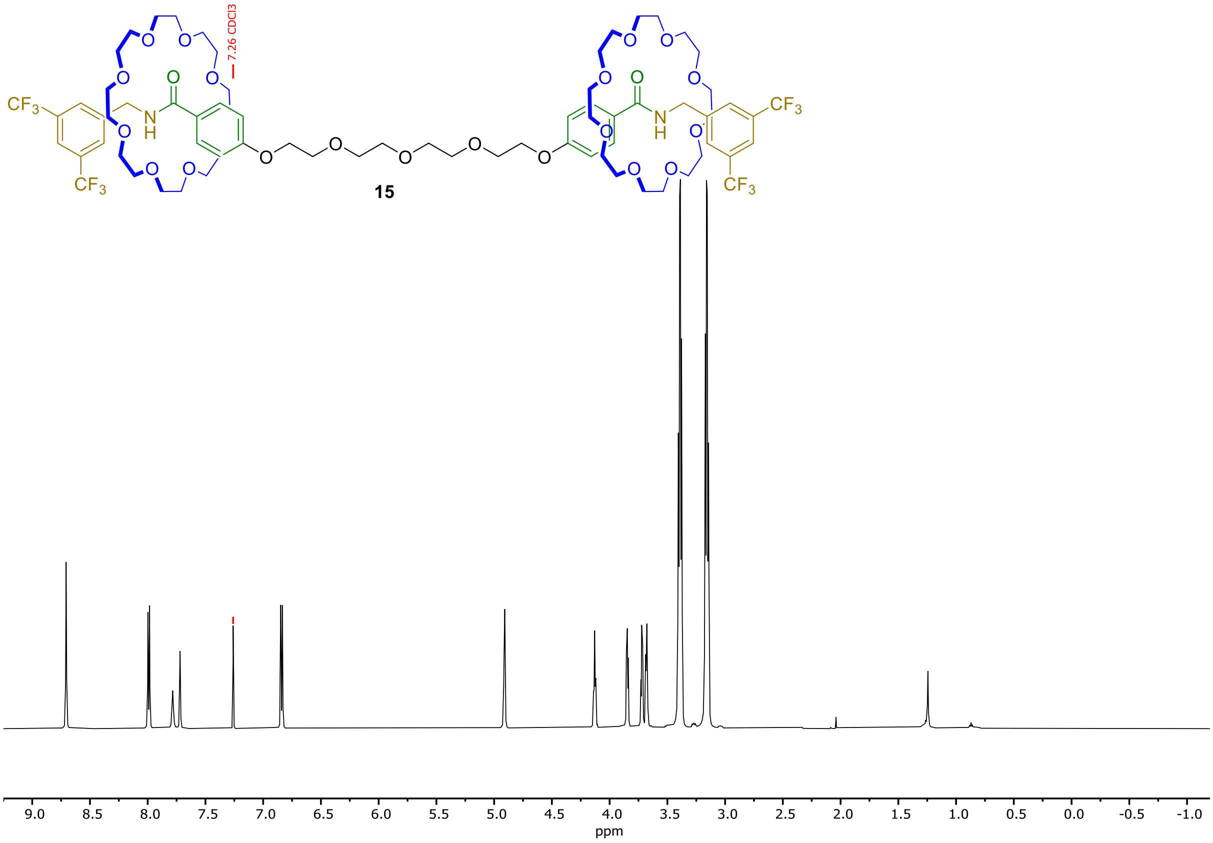


**Spectrum S55**: $^{1}$H NMR ($CDCl_3$, 600 MHz) of **15**.

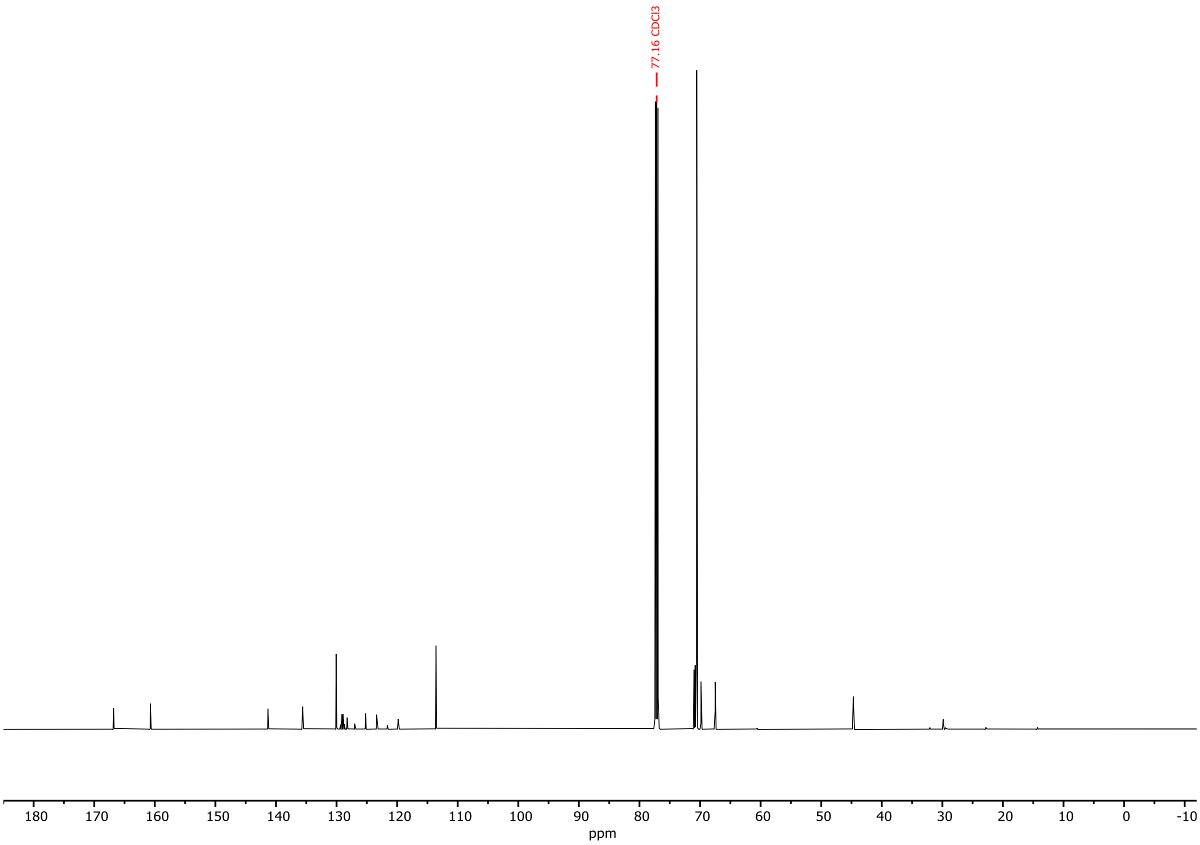


**Spectrum S56**: $^{13}$C NMR ($CDCl_3$, 151 MHz) of **15**.

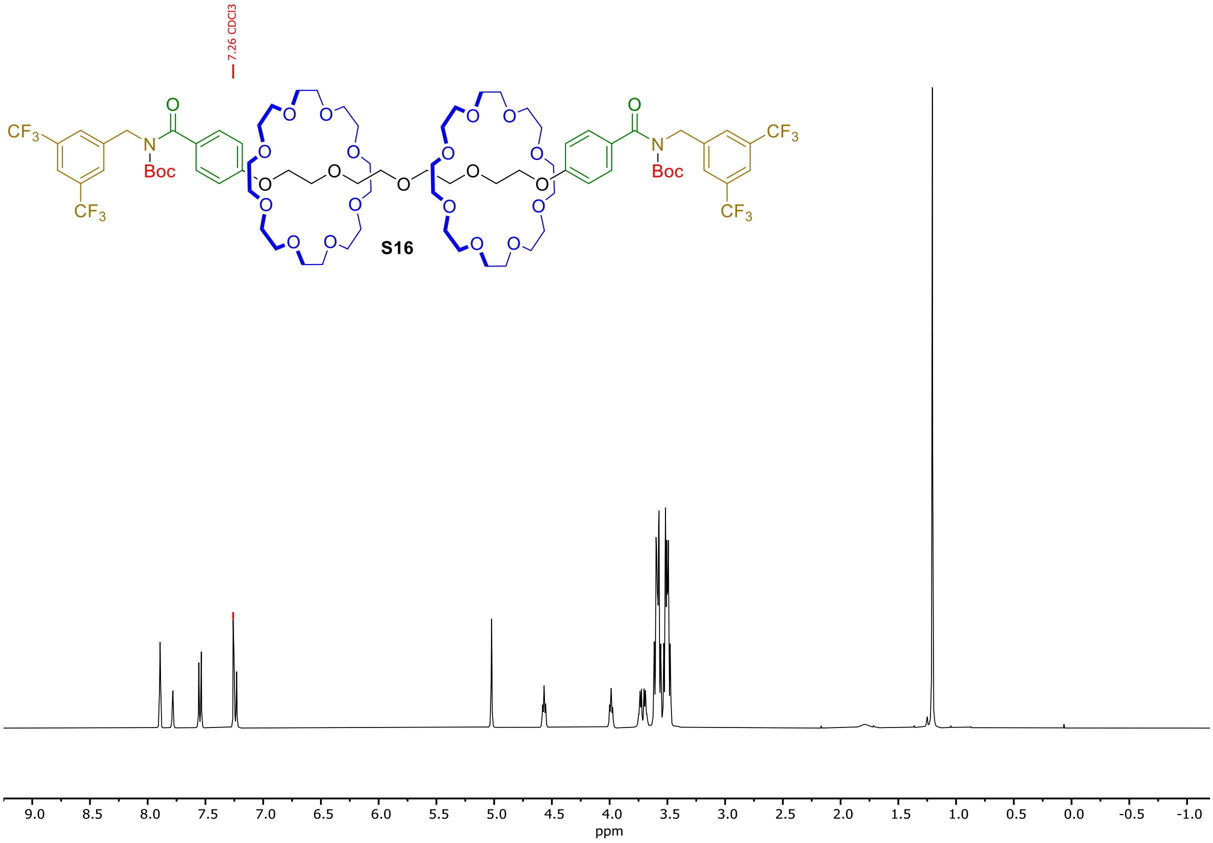


**Spectrum S57**: $^{1}H$ NMR ($CDCl_3$, 400 MHz) of **S16**.

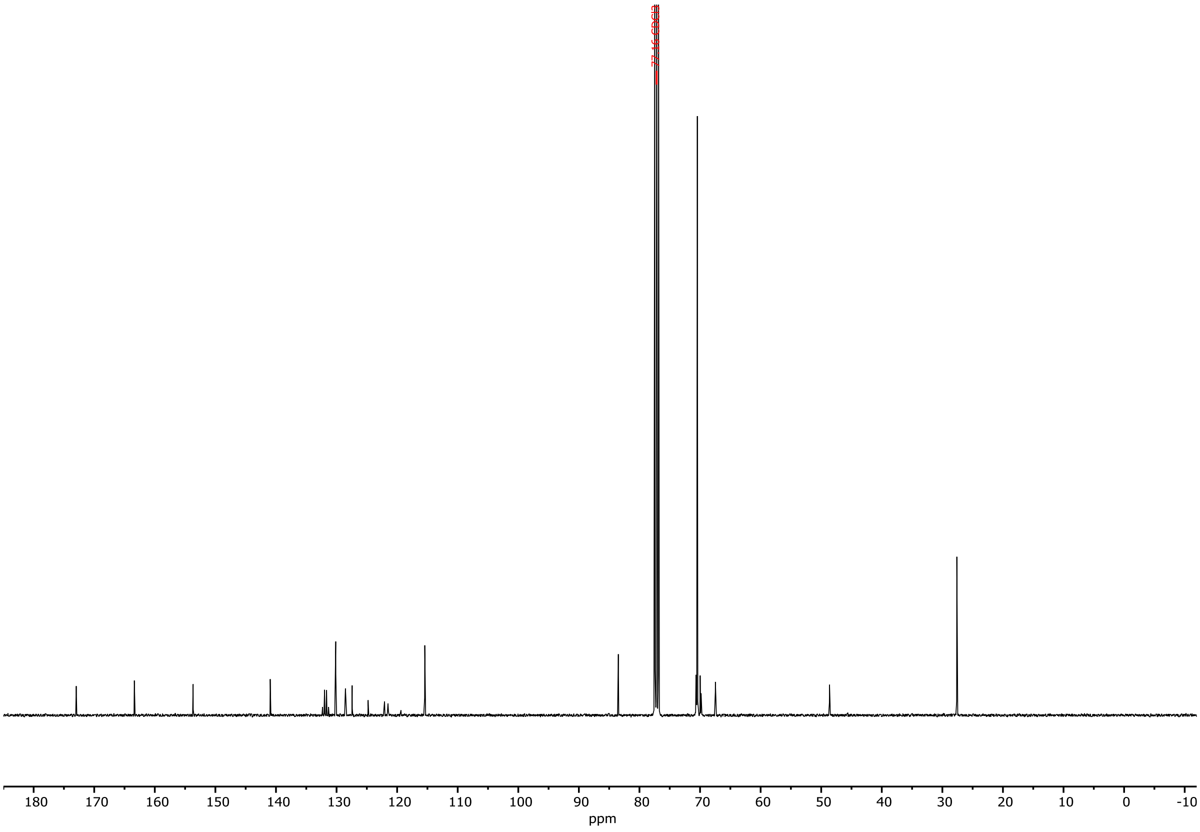


**Spectrum S58**: $^{13}C$ NMR ($CDCl_3$, 101 MHz) of **S16**.

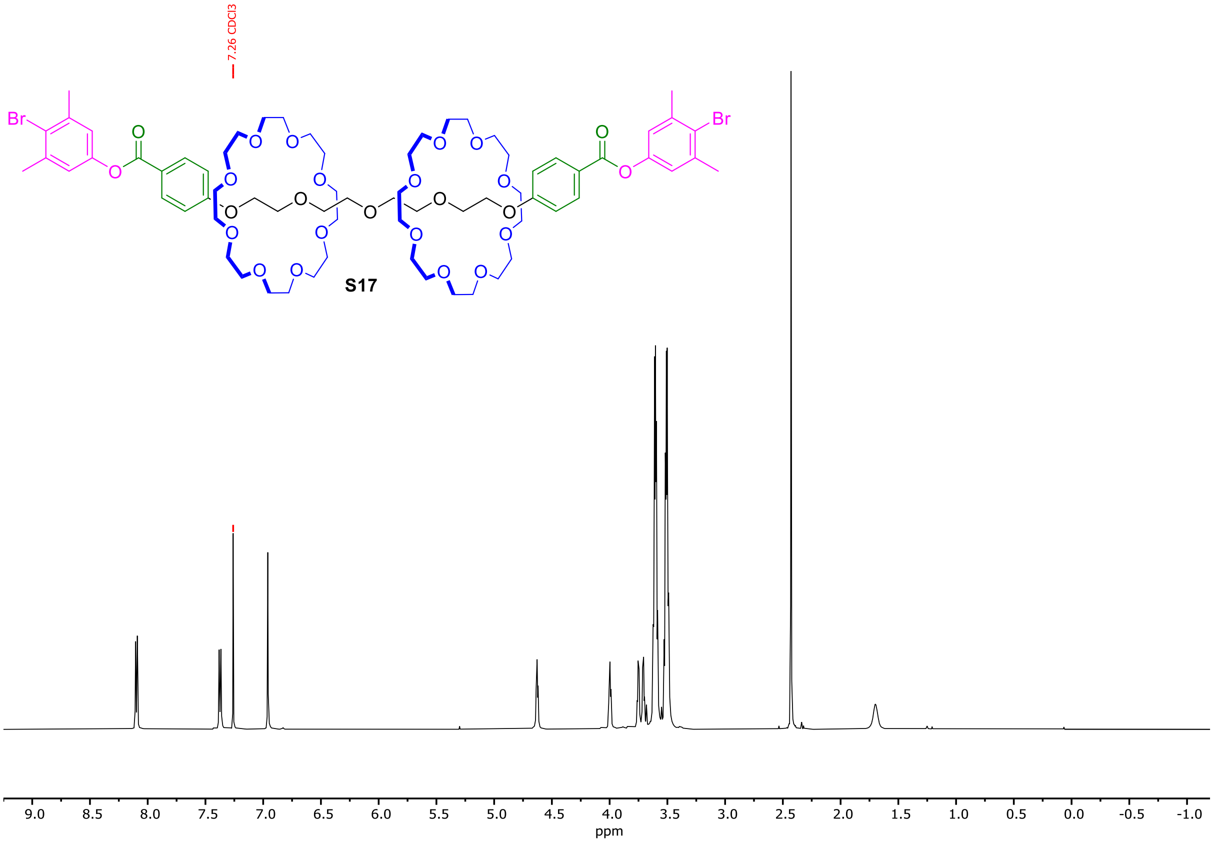


**Spectrum S59**: $^{1}$H NMR ($CDCl_3$, 600 MHz) of **S17**.

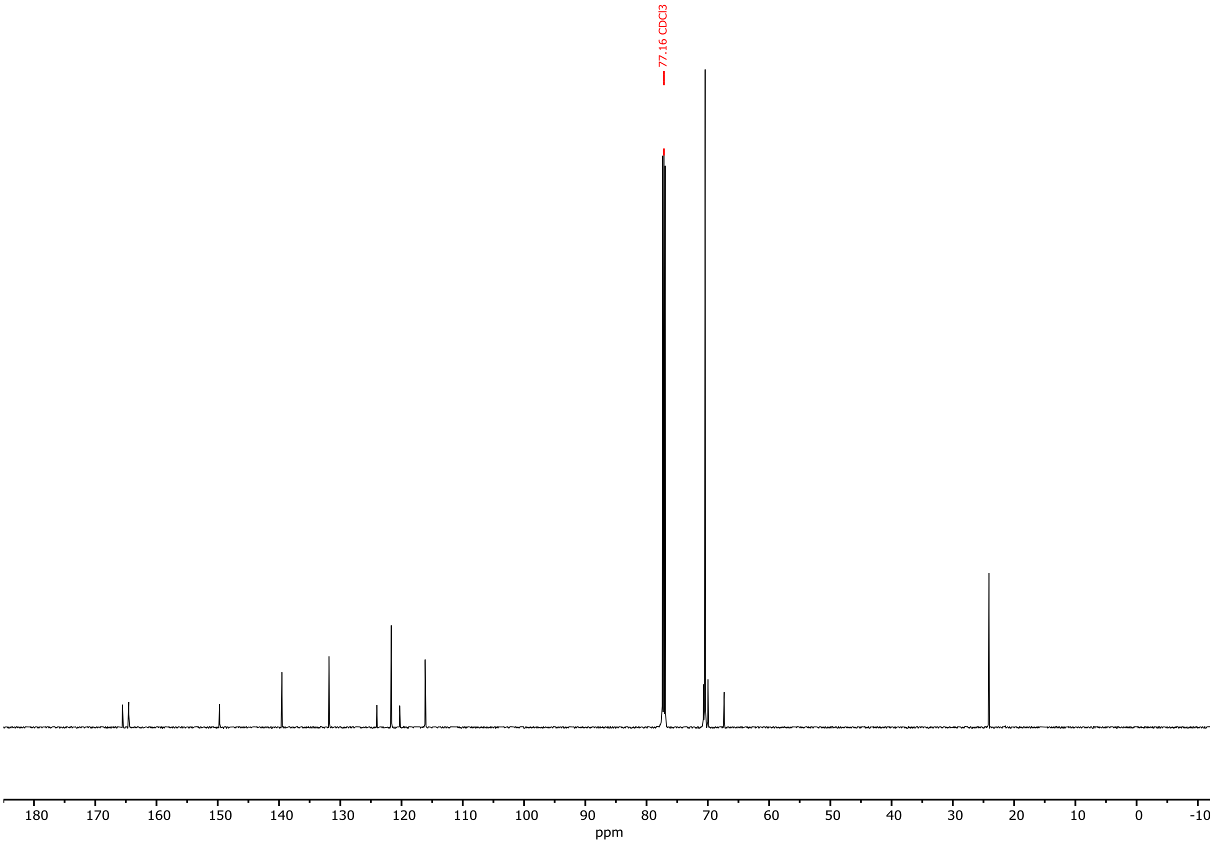


**Spectrum S60**: $^{13}$C NMR ($CDCl_3$, 151 MHz) of **S17**.

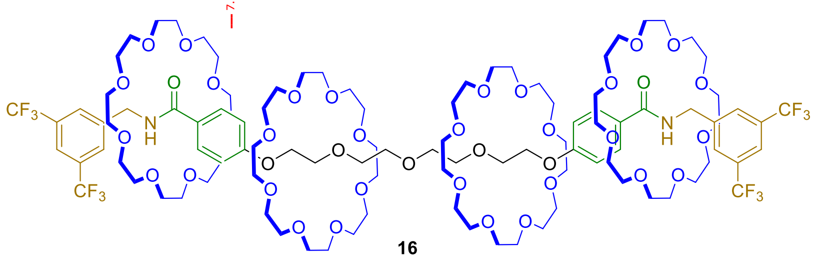

**16**

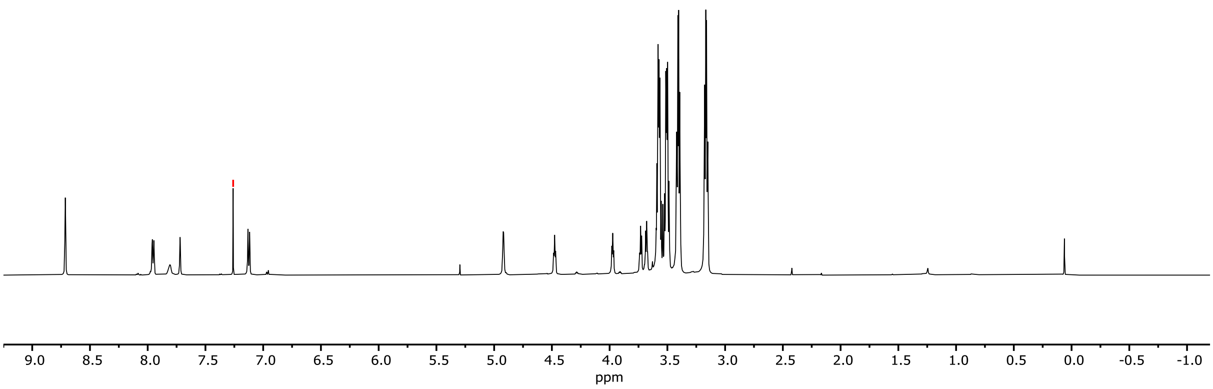


**Spectrum S61**: $^{1}$H NMR ($CDCl_3$, 600 MHz) of **16**.

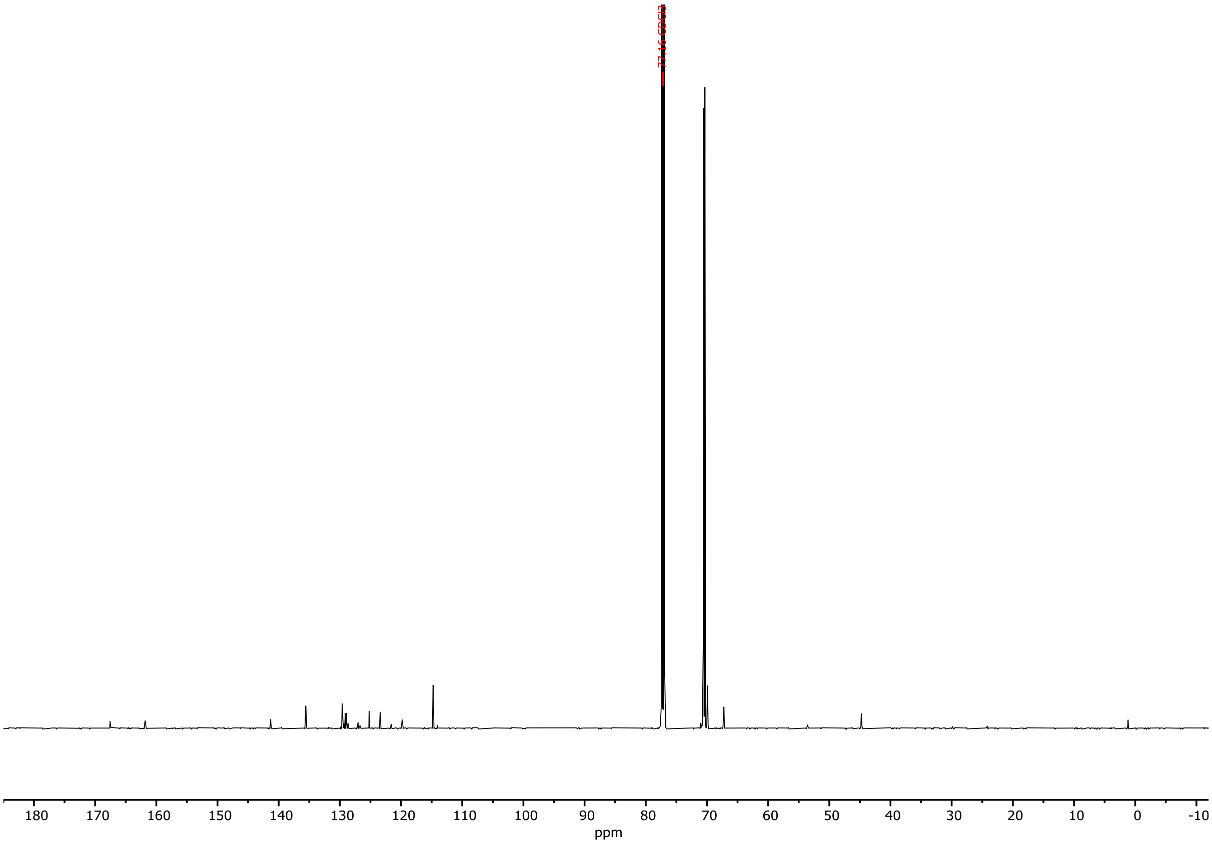


**Spectrum S62**: $^{13}$C NMR ($CDCl_3$, 151 MHz) of **16**.

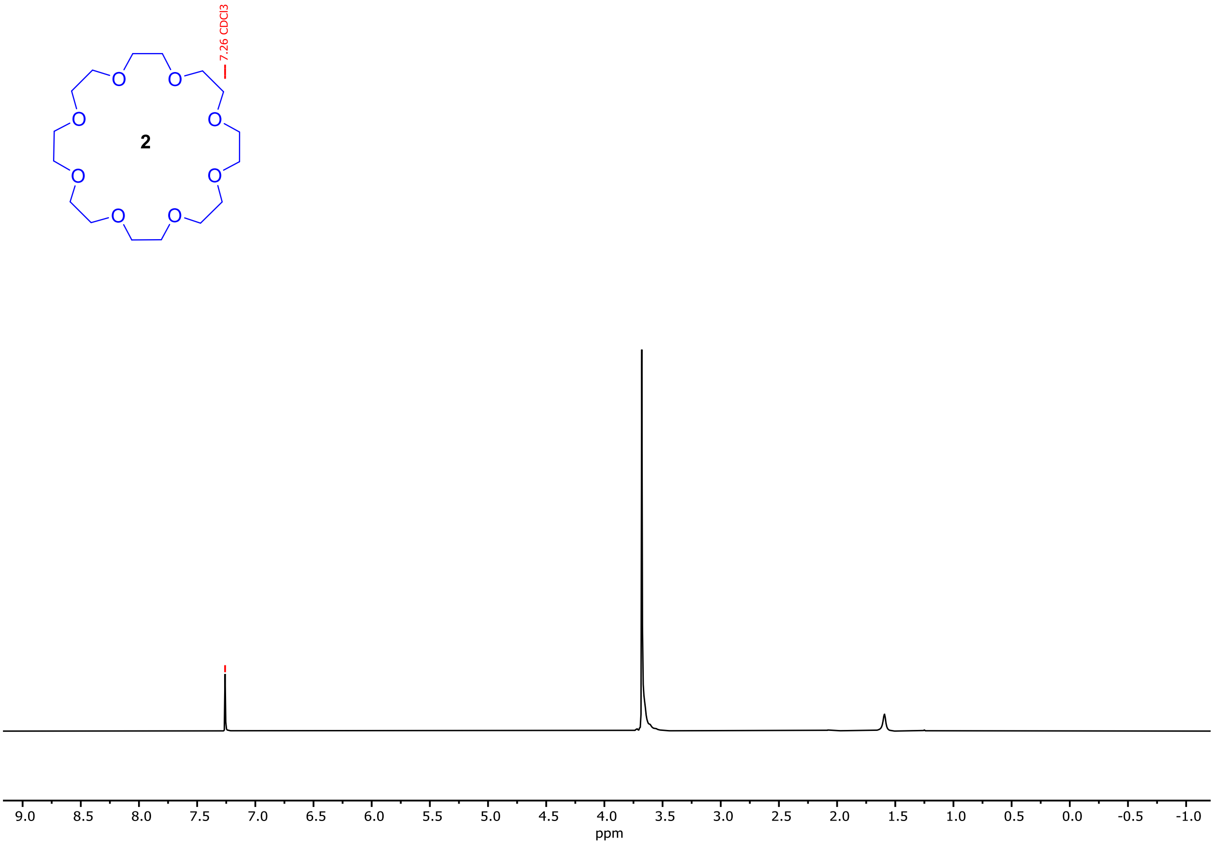


**Spectrum S63**: $^1$H NMR ($CDCl_3$, 600 MHz) of **2**.

# 10 References

(S1) Nowell, H.; Barnett, S. A.; Christensen, K.E.; Teat, S. J.; Allan, D. R. I19, the small-molecule single-crystal diffraction beamline at Diamond Light Source. *J. Synchrotron Radiat.* **2012**, *19*, 435-441.

(S2) a) Sheldrick, G. M. Crystal structure refinement with SHELXL. *Acta Crystallogr. Sect. C Struct. Chem.* **2015**, *71*, 3–8. b) Dolomanov, O. V.; Bourhis, L. J.; Gildea, R. J.; Howard, J. A. K.; Puschmann, H. OLEX2: a complete structure solution, refinement and analysis program. *J. Appl. Crystallogr*. **2009**, *42*, 339–341.

(S3) 2Fo-Fc electron density maps were obtained using Phenix software. Initially, the reflections file (.mtz) obtained form CrysAlisPro was edited to include the map coefficients. Then, the maps were calculated from the edited reflections file and the model (pdb file) obtained from structure solution software (OLEX 2) using phenix.maps software.
